# Secretome analysis of patient-derived GBM tumor spheres identifies midkine as a potent therapeutic target

**DOI:** 10.1038/s12276-019-0351-y

**Published:** 2019-12-06

**Authors:** Suji Han, Hyemi Shin, Jin-Ku Lee, Zhaoqi Liu, Raul Rabadan, Jeongwu Lee, Jihye Shin, Cheolju Lee, Heekyoung Yang, Donggeon Kim, Sung Heon Kim, Jooyeon Kim, Jeong-Woo Oh, Doo-Sik Kong, Jung-Il Lee, Ho Jun Seol, Jung Won Choi, Hyun Ju Kang, Do-Hyun Nam

**Affiliations:** 1 Institute for Refractory Cancer Research, Research Institute for Future Medicine, Sungkyunkwan University, Seoul, Korea; 2 Department of Health Sciences and Technology, Samsung Advanced Institute for Health Science & Technology (SAIHST), Sungkyunkwan University, Seoul, Korea; 3 Department of Biochemistry and Molecular Biology, Ajou University School of Medicine, Suwon, Korea; 4 Department of Systems Biology, Columbia University, New York, NY USA; 5 Department of Biomedical Informatics, Columbia University, New York, NY USA; 6 Department of Cancer Biology, Lerner Research Institute, Cleveland Clinic, Cleveland, OH USA; 7 Center for Theragnosis, BRI, Korea Institute of Science and Technology, Seoul, Korea; 8 Department of Biomolecular Science, University of Science and Technology, Daejeon, Korea; 9 Department of Anatomy and Cell Biology, Sungkyunkwan University, Seoul, Korea; 10 Department of Neurosurgery, Samsung Medical Center, Sungkyunkwan University, Seoul, Korea

**Keywords:** CNS cancer, Oncogenes, CNS cancer, Oncogenes, CNS cancer

## Abstract

Glioblastoma (GBM) is the most lethal primary brain tumor with few treatment options. The survival of glioma-initiating cells (GICs) is one of the major factors contributing to treatment failure. GICs frequently produce and respond to their own growth factors that support cell proliferation and survival. In this study, we aimed to identify critical autocrine factors mediating GIC survival and to evaluate the anti-GBM effect of antagonizing these factors. Proteomic analysis was performed using conditioned media from two different patient-derived GBM tumor spheres under a growth factor-depleted status. Then, the antitumor effects of inhibiting an identified autocrine factor were evaluated by bioinformatic analysis and molecular validation. Proteins secreted by sphere-forming GICs promote cell proliferation/survival and detoxify reactive oxygen species (ROS). Among these proteins, we focused on midkine (MDK) as a clinically significant and pathologically relevant autocrine factor. Antagonizing MDK reduced the survival of GBM tumor spheres through the promotion of cell cycle arrest and the consequent apoptotic cell death caused by oxidative stress-induced DNA damage. We also identified PCBP4, a novel molecular predictor of resistance to anti-MDK treatment. Collectively, our results indicate that MDK inhibition is an important therapeutic option by suppressing GIC survival through the induction of ROS-mediated cell cycle arrest and apoptosis.

## Brain cancer: a key growth factor

Targeting the growth factor midkine (MDK) may offer improved treatment for glioblastoma, the most lethal brain cancer. In glioblastoma, the tumor-initiating cells produce their own growth factors, encouraging themselves to keep multiplying, and making glioblastoma very difficult to treat. Do-Hyun Nam and Hyun Ju Kang at the Samsung Medical Center, Seoul, South Korea and coworkers screened the growth factors produced by two glioblastoma samples, then focused on a single cancer-associated factor, MDK. Antagonizing MDK slowed tumor cell growth, and further investigation showed that suppressing MDK restored the susceptibility of tumor cells to DNA damage and the mechanisms that weed out damaged cells. Noting that treatment efficacy varied between tumor samples, the researchers identified a biomarker for sensitivity to MDK treatment. These results point the way to new treatments for this particularly deadly cancer.

## Introduction

Glioblastoma (GBM) is the most lethal cancer in the adult brain with a dismal prognosis^1^. Despite intensive treatment that includes maximal surgery and chemoradiotherapy using temozolomide, the median survival time of GBM patients is only 15 months. Clinical approaches to target genetic alterations have shown limited clinical responses, which emphasizes the need for the identification of biologically relevant molecular targets that might perform pivotal functions in mediating GBM cell proliferation and/or malignancy^2^.

Glioma-initiating cells (GICs), an undifferentiated stem-like cell subpopulation, frequently resemble classical neurospheres (termed GBM tumor spheres herein) and show self-renewal and oncogenic transforming properties, which are crucially important in therapeutic resistance and tumor recurrence after treatment^3^. Although intensive studies have revealed the molecular mechanisms underlying the survival of GICs, few molecules have been identified as effective therapeutic targets to abolish this subpopulation^4^. Cancer cells frequently produce and respond to their own growth factors (autocrine factors) in order to enhance cell proliferation by both activating growth signaling pathways and inhibiting apoptosis-associated signaling cascades^5,6^. Antagonizing these autocrine factors secreted by GICs may be a therapeutic option via the interruption of GIC maintenance.

Here, through a liquid chromatography-mass spectrometry (LC-MS)-based secretome analysis, we identified midkine (MDK) as an autocrine factor in patient-derived GBM tumor spheres. MDK is a heparin-binding growth factor that affects various biological processes, including fibrinolysis, apoptosis inhibition, mitogenesis, angiogenesis, neural lineage commitment regulation, and tumorigenesis^7,8^. MDK has also been identified as a potential predictive and diagnostic marker to predict the clinical efficacies of chemotherapies in various malignancies^9,10^. Recent studies revealed that MDK contributes to tumor metastasis via the lymphatic vessels through systemic induction of neo-lymphangiogenesis^11^. Moreover, MDK is frequently overexpressed in various malignant tumors, including GBM, and confers resistance to chemotherapy-induced cell death by protecting cells from apoptosis-associated cellular cascades^12^. However, the molecular mechanisms of such actions have not been fully clarified.

This study showed the therapeutic efficacy of antagonizing MDK in GBM tumor spheres. We also revealed a novel mechanism by which MDK inhibition induces cell cycle arrest and consequent apoptosis-associated cell death by enhancing reactive oxygen species (ROS) stress-mediated DNA damage. In addition, we identified PCBP4 as a potential molecular predictor for resistance and a candidate for combination with MDK-antagonizing therapies.

## Materials and methods

### Patient-derived GBM specimens and primary tumor sphere culture

All surgical specimens were acquired from GBM patients at the Samsung Medical Center (Seoul, Korea) in accordance with the valid Institutional Review Board policies. Tumor specimens were dissociated into single cells and cultured under serum-free conditions^13^.

### Secretome and protein array analyses

Proteolytic digestion of proteins, fractionation of peptides and mass spectrometric analysis were performed as described previously^14^. Biological functions were analyzed using the DAVID functional classification tool (https://david.ncifcrf.gov/) and the ClueGO Cytoscape plugin^15^. Protein array data were obtained using a Phospho Explorer Antibody Array (# PEX100, Full Moon Biosystems, CA, USA).

### Cell viability and sphere formation assay

Cell viability was analyzed using an adenosine triphosphate (ATP) monitoring system based on firefly luciferase (ATPLite1step, PerkinElmer, MA, USA), and luminescence was measured using an EnVision multilabel plate reader (PerkinElmer, MA, USA)^16^. An EdU fluorescence assay was carried out using a BCK-EdU594 kit (Sigma-Aldrich, MO, USA). Tumor spheres were imaged and analyzed using the Operetta/Harmony High Content Imaging System (PerkinElmer, MA, USA). A limiting dilution assay (LDA) was also performed in 96-well plates, and cells were seeded in a range of 1–200 cells per well.

### Lentivirus production and transduction

MDK and PCBP4 knockdown shRNA lentiviral clones were purchased from Sigma-Aldrich, and the pLenti-PCBP4 expression vector was obtained from abm. Lentiviruses were produced in 293FT cells with a packaging mix (ViraPower Lentiviral Expression Systems, Thermo Fisher, MA, USA). Stable transfectants were selected by incubation with puromycin (1−2 ng/ml).

### Orthotopic GBM xenograft models

All animal experiments were approved by the Institutional Review Board of the SMC and performed according to the guidelines of the Animal Use and Care Committees. Cells (1 × 10^4^ per mouse) were resuspended in a volume of 5 μl and were then stereotactically injected into the brains of BALB/c nude mice (Orient Bio Inc., Korea).

### RNA sequencing and bioinformatic analysis

A sequencing library was prepared using an Illumina TruSeq RNA Library Preparation Kit v2 in four samples. The genome index was generated using a file containing the annotated human genome (GRCh37), version 19 (Ensembl 74), from GENCODE. The featureCounts function from the “Subread” package was adopted to calculate Reads Per Kilobase per Million mapped reads (RPKM) values. The detailed analysis methods are supplied in the supplementary materials.

### DNA damage analysis

P-γH2AX, a marker of DNA double-strand breaks (DSBs), was detected using an OxiSelect DNA DSB staining kit (# STA-321, Cell Biolabs, CA, USA) according to the manufacturer’s instructions. An alkaline single-cell gel electrophoresis assay to detect DNA damage was performed with an OxiSelect Comet Assay Kit (# STA-355, Cell Biolabs, CA, USA)^17^.

### Cell cycle analysis

To analyze the cell cycle, single cells dissociated from GBM tumor spheres were fixed with 100% ethanol and incubated at 4 °C overnight. Cells were stained with propidium iodide (PI, Sigma-Aldrich, MO, USA) and analyzed using flow cytometry (FACS Aria, BD Biosciences, CA, USA).

### Apoptosis assay

Single cells were resuspended in Annexin V Binding Buffer (BD Pharmingen, CA, USA) and were then stained with Annexin V-APC solution (eBioscience, CA, USA). Caspase 3/7 activity was measured using CellEvent™ Caspase-3/7 Green Detection Reagent (Thermo Fisher, MA, USA). Apoptosis array analysis was performed using a Proteome Profiler Human Apoptosis Array Kit (ARY009, R&D Biosystems, MN, USA).

### Statistical analysis

Data are presented as the means ± standard deviations (SDs). *p* values were obtained using a two-tailed, unpaired *t* test (GraphPad Prism v.5.03). Statistical significance is displayed as *p < 0.05, **p < 0.01, and ***p < 0.001.

Method details and related references are available in the online supplementary materials.

## Results

### Secretome analysis of patient-derived GBM tumor spheres identifies MDK

**Fig. 1:**
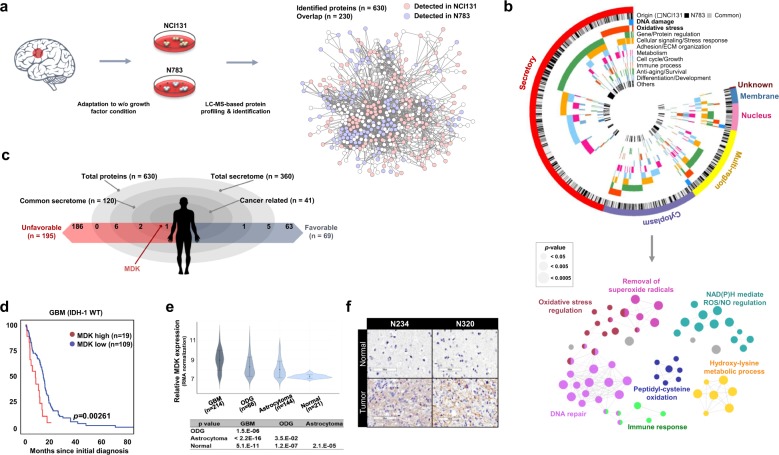
Identification of autocrine factors through the secretome analysis. **a** A schematic diagram describing the experimental procedures. **b** A Circos plot showing the subcellular localization, source and functional classification based on Gene Ontology (GO) analysis using the DAVID web tool (top). ClueGO analysis for functionally grouped networks of enriched categories for representative biological processes. The node size indicates the significance of the term *p* value corrected via the Bonferroni step down approach (bottom). **c** A schematic demonstrating the stratification of identified proteins. The association with cancer and the prognostic influence were determined using the DAVID web tool (GAD disease class, cancer) and the Human Protein Atlas web tool, respectively. **d** Kaplan−Meier analysis of survival in a dataset of IDH-1 wild-type (WT) GBM patients from The Cancer Genome Atlas (TCGA) according to their MDK level. **e** MDK mRNA expression level across normal brain and glioma specimens with different histological grades in a Rembrandt dataset. **f** Immunohistochemical analyses of MDK expression in GBM specimens. The bar represents 100 µm.

To identify the autocrine factors, we selected two different patient-derived tumor spheres (NCI131 and N783) that were capable of proliferation without growth factor supplementation. In-gel trypsin-digested peptides from conditioned media were then subjected to LC-MS analysis (Fig. 1a). After a search of the UniProtKB database, 630 unique proteins were identified^18^. Of these, 471 and 389 were identified from NCI131 and N783, respectively, and 230 proteins were common to both tumor spheres (Fig. 1a, Supplementary Table 1). In total, ~60% (360/630) of the proteins were predicted to be secreted (via UniProtKB and PantherDB; Supplementary Tables 1, 2, Fig. 1b). Among the secreted proteins, 147 and 93 were identified from NCI131 and N783, respectively, while 120 proteins were common to both tumor spheres.

The gene ontology (GO) biological process (GOBP) algorithm in the DAVID web tool^19^ and ClueGO analysis identified functional networks of the unique proteins (*n* = 630) governing redox regulation-related biological processes, including the terms removal of superoxide radicals, oxidative stress regulation, NADPH-mediated ROS/NO regulation, peptidyl-cysteine oxidation and hydroxylysine metabolic process, and DNA repair (Fig. 1b, Supplementary Fig. 1a, b)^15,20^.

Among the secretome common to both tumor spheres (*n* = 120), we identified 41 proteins associated with cancer development and propagation (via the DAVID web tool Genetic Association Database (GAD); *p* = 6.1e-4 by a modified Fisher’s exact test)^21^. The Human Protein Atlas web tool was used to evaluate the prognostic influence of a particular gene (The Human Protein Atlas: The glioma proteome; https://www.proteinatlas.org/humanproteome/pathology/glioma), and 195 and 68 genes were associated with unfavorable and favorable prognoses, respectively, in glioma patients^22^. Nine unfavorable prognosis-associated and six favorable prognosis-associated proteins were detected in our proteome data, in which MDK (Supplementary Fig. 1c) was the only protein associated with unfavorable prognosis in glioma patients (Fig. 1c). The cumulative survival fraction of MDK-high GBM patients was significantly decreased compared to that of MDK-low patients (cutoff: RNAseq V2 RSEM *z*-score = 0.913; 128 samples from the cBioPortal TCGA Dataset, *p* *=* 0.00261 by the log-rank test; Fig. 1d, Supplementary Table 3). The mRNA expression of MDK was notably higher in GBM than in oligodendroglioma (ODG) or astrocytoma, while it was significantly lower in nontumor brain tissues (Rembrandt Dataset, Affymetrix HG U133 v2.0 Plus; Fig. 1e). We also observed that MDK promoter methylation was significantly decreased but mRNA expression was increased in high-grade tumors compared to low-grade gliomas. However, there were no significant differences in the copy number status between low- and high-grade tumors (TCGA low-grade glioma_glioblastma_provision, Supplementary Fig. 2a). These findings suggest that hypomethylation of the MDK promoter may be a mechanism contributing to the high MDK expression in high-grade tumors. Consistent with this result, intense MDK immunoreactivity was detected in GBM tumors from N234 and N320 patients and in NCI131 xenograft tissues compared to adjacent normal brain tissues (Fig. 1f, Supplementary Fig. 2b). We also observed that 8 of 11 GICs expressed MDK, and 5 of these showed notably high expression (Supplementary Fig. 2c). The MDK expression level in different parts of the same tumor was generally stable, with few fluctuations (Supplementary Fig. 2d)^16^.

### MDK inhibition attenuated the survival and proliferation of GBM tumor spheres

MDK increased the tumor sphere numbers (14 days, *p* < 0.0001, Supplementary Fig. 3a, b) and enhanced the activity of AKT, STAT3 and ERK (Supplementary Fig. 3c)^23^.

**Fig. 2:**
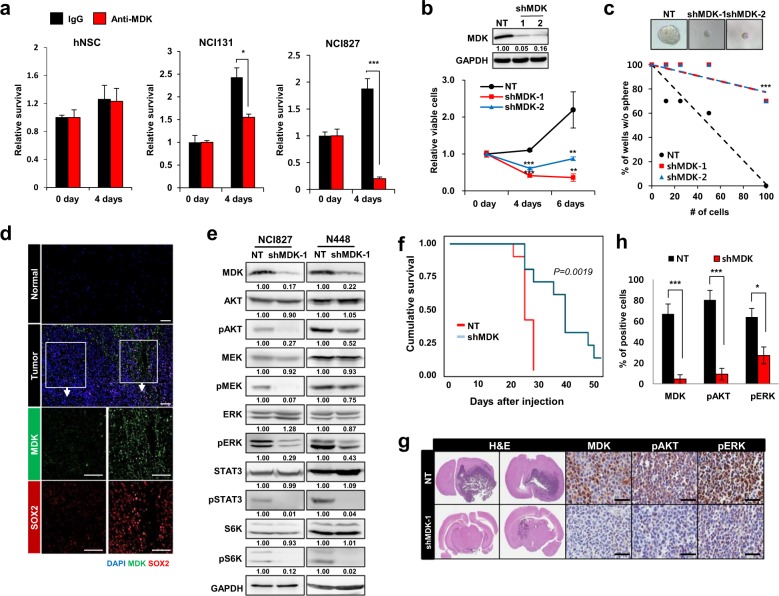
The effect of MDK on the proliferation and survival of GBM tumor spheres. **a** The effect of treatment with control IgG (black bar) or anti-MDK (red bar, 5 µg/ml for 4 days) on the relative survival of hNSCs (left), NCI131 cells (middle) and NCI827 cells (right) is shown in bar graphs. **b** Immunoblots of MDK expression in cells transfected with nontargeting (NT) shRNA or two shMDK constructs are shown (top). The data are representative of three independent experiments. The relative viabilities of NCI827 cells transfected with NT shRNA (black lines) or two different shMDK constructs (red and blue lines) at the indicated time points are shown as a line graph (bottom). **c** Limiting dilution assay (LDA) in NCI827 cells transfected with NT shRNA (black) or two different shMDK constructs (red and blue), and representative sphere images are shown. **d** Immunofluorescence analysis of MDK (green), SOX2 (red) and/or DAPI (blue) on fresh frozen specimens from GBM tumor (G047) and matched normal tissues. The data are representative of three independent experiments. The scale bars indicate 100 µm. **e** Western blot analyses of the indicated proteins in NCI827 (left) and N448 (right) cells transfected with NT shRNA or an shMDK construct. The data are representative of three independent experiments. **f** Kaplan−Meier cumulative survival plot of mice intracranially injected NCI827 cells transfected with NT shRNA (red) or shMDK (blue) (*n* *=* 10 per group). **g** Hematoxylin and eosin (H&E) and immunohistochemical staining analysis for detecting the indicated proteins in tumor-bearing brains injected with NT shRNA- or shMDK-transfected NCI827 cells. The scale bars indicate 50 µm. **h** The percentage of cells positive for the indicated proteins in intracranial tumors derived from NCI827 cells transfected with NT shRNA (black) or shMDK (red), as shown in (**g**), is shown as a bar graph. *p < 0.05, **p < 0.01, and ***p < 0.001.

Antagonizing MDK significantly reduced GBM cell survival (*p* < 0.5 and <0.001 for NCI131 and NCI827, respectively; Fig. 2a, Supplementary Fig. 4a, b) and tumor sphere formation (*p* *<* 0.001, Supplementary Fig. 4c). Knockdown of MDK significantly reduced cell survival (*p* = 0.0019, Fig. 2b). Clonogenic growth was significantly decreased upon MDK knockdown in both NCI131 (*p* *=* 1.55e-07 and 2.06e-03 for nontargeting shRNA vs. shMDK-1 and shMDK-2, respectively; Supplementary Fig. 5) and NCI827 cells (*p* *=* 1.82e-06 and 1.82e-06 for nontargeting shRNA vs. shMDK-1 and shMDK-2, respectively; Fig. 2c, Supplementary Table 4).

Sox2 expression was attenuated by anti-MDK treatment (Supplementary Fig. 6a)^24^. The spatial expression of MDK was closely correlated with that of SOX2 in a GBM tumor specimen (Fig. 2d). Under serum-induced differentiation conditions, the expression level of MDK was notably decreased in three different tumor spheres (Supplementary Fig. 6b). Moreover, the expression of YKL-40 and SSEA-1, enrichment markers of tumor stem cells, was downregulated upon MDK knockdown (Supplementary Fig. 6c)^25^.

We also observed that the activity of AKT, MEK, ERK, STAT3 and S6K was notably decreased following either MDK knockdown (Fig. 2e) or anti-MDK treatment (Supplementary Fig. 7a)^23^. In addition, the activity of several previously reported MDK receptors, including ALK and intracellular NOTCH2, was decreased upon treatment with an anti-MDK antibody (Supplementary Fig. 7b)^26,27^.

The Kaplan−Meier survival curve showed a significant increase in the survival of mice injected with MDK knockdown cells compared that of mice injected with NT cells (*n* = 10, *p* *=* 0.00019 by the log-rank test; Fig. 2f). The tumors in mice injected with MDK-deficient cells were smaller than those in mice injected with NT cells, and the activity of AKT and ERK was significantly decreased in the MDK knockdown group (Fig. 2g, h).

### MDK inhibition downregulated cell cycle- and proliferation-associated genes but upregulated ROS-associated genes

**Fig. 3:**
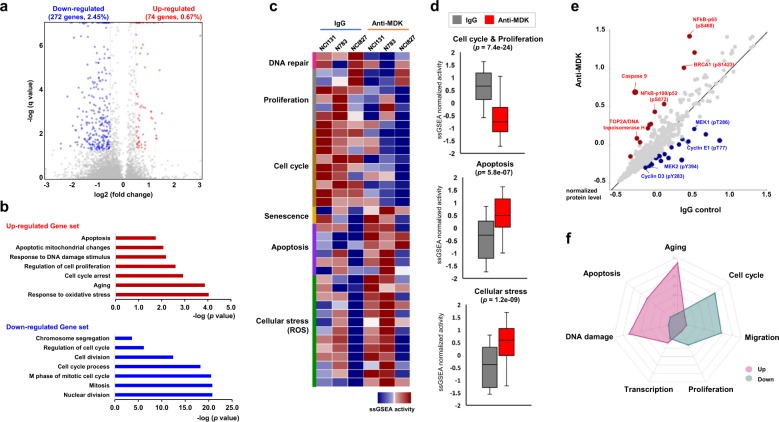
Molecular signatures of antagonizing MDK in GBM tumor spheres. **a** A volcano plot represents differentially expressed genes between anti-MDK antibody- and IgG-treated NCI131, N783 and NCI827 cells. The *x*-axis represents the log2-fold change values, and the *y*-axis represents the log *q*-values. The red or blue dots indicate genes significantly (*q* < 0.05) upregulated (log2-fold change ≥ 0.5) or downregulated (log2-fold change ≤ −0.5) upon anti-MDK treatment (12 h), respectively. **b** The bar charts depict the top ranked pathway analyzed from the functional classification based on the GO biological process using gene expression data from (**a**). The red (top) and blue (bottom) bars represent upregulated and downregulated pathways, respectively, upon anti-MDK treatment compared to IgG treatment. **c** The heatmap shows single-sample gene set enrichment (ssGSEA) enrichment scores analyzed using mRNA expression data from IgG- or anti-MDK-treated (12 h) NCI131, N783 and NCI827 cells, classified according to cellular process. **d** The normalized ssGSEA enrichment scores of three different cells upon treatment with IgG (gray bar) or the anti-MDK antibody (red bar) for the indicated pathways are shown as box plots. **e** A correlation plot of the normalized protein levels in NCI827 cells treated with IgG or anti-MDK (12 h), obtained from a protein array, is shown. The red and blue dots represent proteins upregulated and downregulated, respectively, upon treatment with anti-MDK compared to IgG control. **f** A radar plot of the fold enrichment of the indicated pathways in accordance with their functional classifications (GOBP on DAVID) using protein expression data from (**e**) is shown. The fold change in enrichment increases by five on the radar plot. The pink and green areas represent upregulated and downregulated pathways, respectively, upon treatment with the anti-MDK antibody compared to control IgG.

We observed that of the 11,112 genes, 74 (0.67%) were significantly upregulated and 272 (2.45%) were downregulated after MDK neutralization (*p* *<* 0.05, log2 [fold change] ≥ 0.5 for upregulated genes or ≤−0.5 for downregulated genes vs. IgG control; Fig. 3a, Supplementary Table 5). Functional classification of the gene expression pattern (via the GOBP algorithm in the DAVID tool) identified that cellular processes associated with apoptosis, cell cycle arrest, DNA damage, and oxidative stress responses were upregulated, while the positive regulation of the cell cycle and cell division processes were significantly downregulated after MDK neutralization (Fig. 3b, Supplementary Fig. 8a, b, Supplementary Table 6). The set of genes associated with cell cycle and proliferation signals was significantly enriched in the control group compared to the MDK inhibition group, while the set of genes associated with DNA damage, such as the response to IR, was upregulated in MDK-inhibited cells (Supplementary Fig. 8c). Furthermore, single-sample gene set enrichment analysis (ssGSEA) revealed that gene sets associated with activities of DNA repair, cellular proliferation, and cell cycle processes (*p* *=* 7.4e-24) were enriched in the IgG control group, whereas pathways associated with cellular senescence, apoptosis (*p* *=* 5.8e-07) and ROS-mediated cellular stress (*p* *=* 1.2e-09) were significantly enriched in MDK neutralized tumor spheres (Fig. 3c, d, Supplementary Fig. 9 and Supplementary Table 7).

Analysis of differentially expressed proteins (DEPs) identified that after MDK neutralization, the expression and activity levels of MEK1/2 and Cyclin D3/E1 were decreased, whereas those of NF-kB-p65 and NF-kB-p100/p52, BRCA1, and TOP2A were enhanced (Fig. 3e)^28,29^. Functional classification (via the GOBP algorithm in the DAVID tool) of the DEP analysis results revealed that after MDK inhibition, the activity of proteins involved in apoptosis, cell cycle arrest, aging, and the DNA damage response was upregulated, whereas that of proteins involved with positive regulation of the cell cycle, migration and cellular proliferation were downregulated (Fig. 3e, f, Supplementary Fig. 10a, b).

### MDK inhibition resulted in oxidative stress-induced DNA damage

**Fig. 4:**
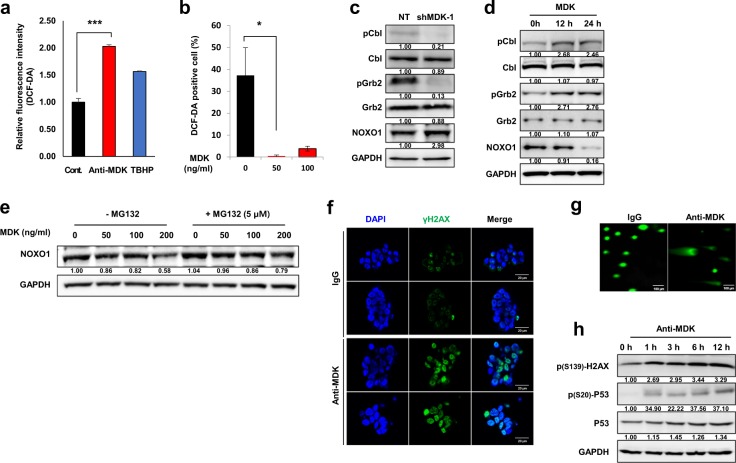
The effect of MDK inhibition on oxidative stress-induced DNA damage. **a** Relative fluorescence intensities upon dichlorofluorescein diacetate (DCF-DA) treatment in the control, anti-MDK (5 µg/ml, 4 h), and tert-butyl hydroperoxide (Sigma, TBHP, 50 µM, 4 h) treated groups are shown as bar graphs. TBHP was used as the positive control for the ROS source. **b** The percentage of DCF-DA-positive cells upon treatment with recombinant MDK at the indicated doses for 6 h is shown in the bar graph. **c** Immunoblots of the indicated proteins in NCI827 cells transfected with nontargeting (NT) shRNA or shMDK or **d** treated with recombinant MDK (50 ng/ml) for 0, 12 or 24 h are shown. The data are representative of three independent experiments. **e** Western blot analyses of NOXO1 in NCI827 tumor spheres upon treatment with recombinant MDK at the indicated doses with or without MG132 (5 µM for 6 h) under the influence of cycloheximide (12.5 µg/ml) are shown. The data are representative of three independent experiments. **f** Immunofluorescence analysis to detect γH2AX (green) and/or DAPI (blue) in NCI827 cells treated with control IgG or the anti-MDK antibody (5 µg/ml each for 5 h) is shown. The scale bars represent 20 µm. The data are representative of three independent experiments. **g** Comet assay in NCI827 cells treated with IgG (left) or the anti-MDK antibody (5 µg/ml each). The scale bars indicate 100 µm. The data are representative of three independent experiments. **h** Western blot analyses of the indicated proteins upon treatment with the anti-MDK antibody (20 µg/ml) for the indicated times are shown. The data are representative of three independent experiments. *p < 0.05, **p < 0.01, and ***p < 0.001.

Consistent with the results of the DEG and DEP analyses, intracellular ROS levels were significantly increased after MDK antagonism (*p* *<* 0.001, Fig. 4a). We also found that treatment with recombinant MDK significantly decreased ROS generation (*p* < 0.05; Fig. 4b, Supplementary Fig. 11b). Previous studies discovered that the activity of NADPH Oxidase 1 (NOX1), one of the major ROS-generating cellular machineries, is regulated by Grb2/Cbl-mediated proteolysis of Nox Organizer 1 (NOXO1)^30^. We observed that knockdown of MDK decreased the activity of Cbl and Grb2, resulting in substantial upregulation of NOXO1 (Fig. 4c). In addition, recombinant MDK treatment increased the activity of Cbl/Grb, resulting in the downregulation of NOXO1 expression in a time-dependent manner (Fig. 4d). We next observed that recombinant MDK treatment decreased the stability of NOXO1 in a dose-dependent manner and that NOXO1 stability was restored by treatment with the proteasome inhibitor MG132 (Fig. 4e). These results indicate that MDK could negatively regulate NOXO1 stability by activating the Cbl/Grb-associated proteolytic axis.

In addition, MDK inhibition increased the activity of γH2AX, a DNA double-strand break (DSB)-sensing molecule (Fig. 4f), and this increase was abolished by treatment with the antioxidant *N*-acetyl cysteine (NAC, Supplementary Fig. 11a)^31^. The comet assay revealed that DNA damage was detected after MDK neutralization (Fig. 4g). Along with the activity of γH2AX, the activity and expression of p53 were increased after treatment with an MDK neutralizing antibody in a time-dependent manner (Fig. 4h)^32^. We also observed that MDK neutralization significantly increased temozolomide (TMZ) sensitivity, likely through the augmentation of ROS-induced DNA damage (Supplementary Fig. 12a–c).

### MDK inhibition led to cell cycle arrest and apoptotic cell death of GBM tumor spheres

**Fig. 5:**
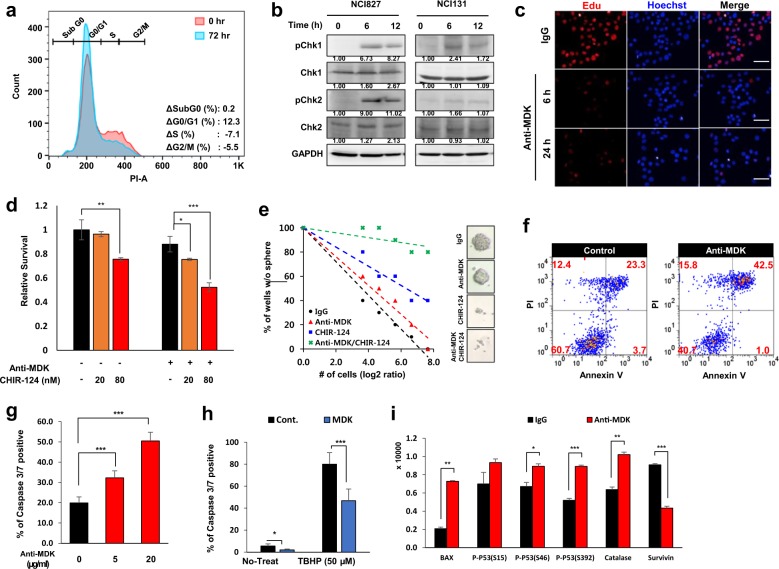
The effect of MDK inhibition on the cell cycle and apoptotic cell death. **a** Flow cytometric cell cycle analyses on control or anti-MDK (1 µg/ml)-treated NCI827 cells at 72 h are shown. The data are representative of three independent experiments. **b** Immunoblots for the indicated proteins upon treatment with the anti-MDK antibody for 0, 6 or 12 h in NCI827 (left) and NCI131 (right) cells are shown. The data are representative of three independent experiments. **c** Immunofluorescence images for EdU (red)- and/or Hoechst (blue)-stained NCI131 cells upon treatment with control IgG or the anti-MDK antibody (5 µg/ml) for 6 or 24 h are shown. The scale bar represents 100 µm. The data are representative of three independent experiments. **d** The relative survival of control cells and cells treated with the anti-MDK antibody (5 µg/ml) with or without CHIR-124 (Selleckchem) at the indicated doses is shown in the bar graphs. **e** An LDA plot and representative images of control spheres and spheres treated with anti-MDK with or without CHIR-124 are shown. **f** Flow cytometric analysis of Annexin V/PI-stained control (left) or anti-MDK (5 µg/ml, right)-treated NCI827 cells is shown. The data are representative of three independent experiments. **g** Quantification of data in immunofluorescence images for detecting caspase 3/7-positive control or anti-MDK antibody-treated NCI827 cells treated at the indicated doses is shown in the bar graph. **h** The percentage of caspase 3/7-positive control (black bar) or recombinant MDK (10 ng/ml, white bar)-treated cells with or without TBHP (50 µM) is shown in the bar graph. **i** The relative intensities of the indicated proteins in the apoptosis array upon treatment with IgG (black) or the anti-MDK antibody (red, 5 µg/ml, 12 h) are shown in the bar graph. *p < 0.05, **p < 0.01, and ***p < 0.001.

We observed a substantial increase in G0/G1 arrest (from 64.7 to 77.0%) upon anti-MDK treatment (Fig. 5a and Supplementary Fig. 13). MDK inhibition also enhanced the activity of Checkpoint kinase (Chk) 1 and 2 (Fig. 5b). Activation of Chk molecules leads to cell cycle arrest via the inactivation of cyclin-dependent kinase (CDK) complexes^33^. The expression of Cyclin A, C and D1 was downregulated after treatment with the anti-MDK antibody (Supplementary Fig. 15a). In addition, DEP analysis showed that the phosphorylation of Cyclin D3 and E1 was decreased after MDK inhibition (Fig. 3e). EdU incorporation, an indicator of S phase entry, was decreased at both 6 and 24 h after treatment with the anti-MDK antibody (Fig. 5c). These results indicate that antagonizing MDK induced cell cycle arrest at G0/G1 phase by activating the Chk1/2-p53 axis, which was followed by downregulation of Cyclin D/E and molecules promoting G1-S phase entry (Figs. 4h, 5a–c)^34^.

Chk1 phosphorylation, induced by MDK inhibition, was abolished by treatment with CHIR-124, a Chk inhibitor (Supplementary Fig. 15b)^35^. The survival of GBM tumor spheres was significantly decreased in a dose-dependent manner after treatment with two independent Chk inhibitors, AZD7762 and CHIR-124 (Fig. 5d, Supplementary Fig. 14a) compared to treatment with MDK neutralization alone^35,36^. Clonogenic growth of GBM cells was also significantly inhibited by combination treatment with Chk inhibitors and an MDK-antagonizing antibody compared to treatment with the anti-MDK antibody alone (Fig. 5e, Supplementary Fig. 14b, Supplementary Table 8).

MDK neutralization increased the proportion of cells (from 23.3 to 42.5%, Fig. 5f) undergoing apoptosis. Furthermore, the sub G0 fraction increased after MDK neutralization at a high dose (20 µg/ml) and increased in a treatment duration-dependent manner (Supplementary Fig. 15c). The percentage of caspase 3/7-positive cells was significantly increased by MDK neutralization in a dose-dependent manner (*p* *<* 0.001; Fig. 5g, Supplementary Fig. 15d). In addition, recombinant MDK significantly decreased the expression of caspase 3/7 in both the control (*p* *<* 0.05, data not shown) and tert-butyl hydroperoxide (TBHP) treatment groups (*p* *<* 0.001; Fig. 5h, Supplementary Fig. 16a). The expression and/or activity of p53 (*p* *<* 0.05 and *p* *<* 0.001 for pS46 and pS392, respectively), the proapoptotic factor Bax (*p* *<* 0.01), and the ROS response molecule catalase (*p* *<* 0.01) were significantly increased, while the expression of a cell survival-associated molecule, survivin (*p* *<* 0.001), was downregulated in the apoptosis protein array after MDK inhibition (Fig. 5i, Supplementary Fig. 16b)^37^.

### PCBP4 expression is associated with sensitivity to anti-MDK treatment

**Fig. 6:**
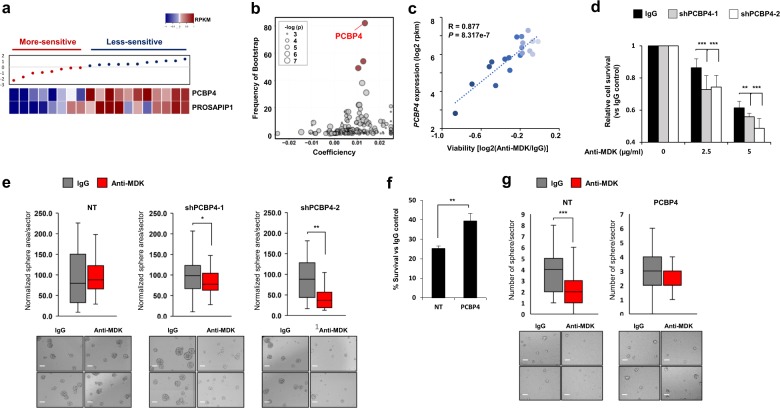
The function of PCBP4 in determining susceptibility to anti-MDK treatment. **a** The relative cell viability upon MDK neutralization (5 µg/ml, 4 days) normalized to the viability of the control IgG-treated group for each cell line, along with the mRNA expression levels of the indicated genes in the corresponding cells, is shown. **b** A volcano plot indicating gene expression features predictive of the anti-MDK antibody response identified by elastic net analysis for their frequency (*y*-axis) and effect size (*x*-axis). The red dots represent genes with a frequency of >40 frequency via the bootstrap method. The node size is proportional to the linear correlation between the drug and the gene expression. **c** Scatter plot showing the linear correlation between *PCBP4* expression (*y*-axis) and viability (*x*-axis). Darker blue dots indicate higher sensitivity to anti-MDK treatment. **d** Relative survival upon anti-MDK treatment at the indicated doses (4 days) normalized to the survival of the IgG control group of N586 cells transfected with NT shRNA or two different shPCBP4 constructs. **e** Sphere areas per sector normalized to those of the IgG control group upon treatment with control IgG or the anti-MDK antibody (5 µg/ml) in N586 cells transfected with NT shRNA and two different shPCBP4 constructs are shown in whisker plots (top). Representative images are presented (bottom). The scale bars represent 100 µm. **f** Percent survival of anti-MDK-treated (5 µg/ml, 4 days) NT shRNA- or ectopic PCBP4-expressing NCI827 cells normalized to that of the corresponding IgG control-treated cells is shown in the bar graph. **g** The number of spheres per sector in the control IgG- and anti-MDK antibody-treated groups of NT shRNA- or ectopic PCBP4-expressing NCI827 cells is shown in whisker plots (top). Representative images of tumor spheres are presented (bottom). The scale bars represent 100 µm. *p < 0.05, **p < 0.01, and ***p < 0.001.

Interestingly, we observed that the sensitivity to MDK neutralization across 19 different GBM tumor spheres was diverse (Supplementary Fig. 17a, b). We identified several genes that were significantly correlated with sensitivity to anti-MDK therapy in GBM tumor spheres using elastic net analysis (Fig. 6a, b). The relative cell viability after MDK neutralization was significantly correlated with the expression of *poly(rC) binding protein 4* (*PCBP4*, *R* *=* 0.877, *p* *=* 8.317e-07, Fig. 6c) among the identified genes. Consistent with this finding, the protein levels of PCBP4 in the group of cells less sensitive to MDK inhibition were higher than those in the “more-sensitive” group (Supplementary Fig. 17a–c).

The relative cell viabilities normalized to those of the vehicle-treated group were significantly decreased in PCBP4-deficient N586 and N446 cells upon MDK neutralization (Supplementary Fig. 17a, b, Fig. 6d). In addition, PCBP4 silencing significantly inhibited tumor sphere formation, while the tumor sphere area of the NT control cells did not decrease upon MDK inhibition (*p* *<* 0.5 and *p* *<* 0.01 for shPCBP4-1 and -2, respectively, Fig. 6e, Supplementary Fig. 18a, b).

The survival fraction upon treatment with anti-MDK was significantly increased in PCBP4-overexpressing GBM cells compared to NT cells (*p* *<* 0.01, Fig. 6f, Supplementary Fig. 18c). Consistent with this finding, the number of tumor spheres was significantly decreased in NT cells but was not attenuated in PCBP4-overexpressing cells after MDK neutralization (*p* *<* 0.001, Fig. 6g).

## Discussion

In this study, we conducted a comprehensive analysis of the cytokine milieu of GICs by performing LC-MS-based proteome analysis using conditioned media from two different GBM tumor spheres with sustained growth under growth factor-free conditions. We found that proteins related to cellular redox homeostasis were significantly enriched in the secretome of GBM tumor spheres^20^. Our data suggest that GICs may protect themselves from ROS by secreting numerous proteins associated with redox homeostasis (Fig. 1).

Among the autocrine proteins, we focused on MDK by stratification according to clinical significance and pathological relevance in GBM malignancy (Fig. 1d, f). Consistent with previous observations, we showed here that MDK inhibition attenuated the growth of both patient-derived GBM models (Fig. 2). A transcriptome and proteome analysis-guided comprehensive evaluation of the molecular signatures revealed that MDK inhibition promoted cellular stress/DNA damage responses, cell cycle arrest and apoptotic cell death, while it attenuated cell proliferation/survival (Fig. 3). These results support the previous observation that MDK inhibition attenuated prostate cancer stem cell growth by inducing cell cycle arrest^38^.

We further proposed several previously unrecognized mechanisms. First, MDK inhibition promotes ROS production by interfering with the Grb/Cbl-dependent proteolytic pathway of NOXO1, a coactivating factor for the NADPH oxidase family, which is a type of cellular machinery for ROS generation (Figs. 3, 4)^30^. Second, intracellular ROS generated by MDK inhibition eventually initiates DNA damage, which sequentially induces cell cycle arrest and/or apoptotic cell death by activating P53 and Chk1/2 while downregulating cyclin D/E and survivin in GBM tumor spheres (Figs. 3, 5). Based on these findings, we suggested and evaluated the synthetic lethal profile of combined treatment with Chk1/2 inhibitors and MDK neutralization, which showed significant enhancement of the anti-MDK therapeutic efficacy in GICs (Fig. 5d, e). Exposing a cell to genotoxic stress, including ROS damage, can result in the formation of DSBs, which destabilize genome integrity. Thus, to repair these breaks, cells activate the cell cycle arrest factors, including Chk1/2 molecules, and recruit DNA repair factors to the break sites^39^. Inhibition of the cell cycle checkpoint molecules results in the accumulation of DSBs in the nucleus without proper repair of the breaks, inducing nuclear condensation and cell death via the caspase 3-dependent apoptosis pathway^40^. Last, we identified low expression of PCBP4 as a biomarker for predicting the therapeutic efficacy of anti-MDK treatments; however, the underlying mechanisms need to be further investigated (Fig. 6). PCBP4, an RNA-binding protein (RBP), plays an important role in various posttranscriptional processes, including mRNA stability, alternative splicing, and translation^41^. Because of the potential risk of spontaneous tumor generation, we also suggest the development of strategies to simultaneously block PCBP4-associated tumorigenic molecules such as ZFP871^41^ in the suppression of PCBP4 in vivo.

Collectively, our results support the potential benefit of anti-MDK therapies, encouraging the development of brain-penetrable MDK-inhibiting molecules for the treatment of GBM patients.

## Supplementary information

Supplementary Information
