# Correction: Secretome analysis of patient-derived GBM tumorspheres identifies midkine as a potent therapeutic target

**DOI:** 10.1038/s12276-020-00517-3

**Published:** 2020-11-04

**Authors:** Suji Han, Hyemi Shin, Jin-Ku Lee, Zhaoqi Liu, Raul Rabadan, Jeongwu Lee, Jihye Shin, Cheolju Lee, Heekyoung Yang, Donggeon Kim, Sung Heon Kim, Jooyeon Kim, Jeong-Woo Oh, Doo-Sik Kong, Jung-Il Lee, Ho Jun Seol, Jung Won Choi, Hyun Ju Kang, Do-Hyun Nam

**Affiliations:** 1 Institute for Refractory Cancer Research, Research Institute for Future Medicine, Sungkyunkwan University, Seoul, Korea; 2 Department of Health Sciences and Technology, Samsung Advanced Institute for Health Science & Technology (SAIHST), Sungkyunkwan University, Seoul, Korea; 3 Department of Biochemistry and Molecular Biology, Ajou University School of Medicine, Suwon, Korea; 4 Department of Systems Biology, Columbia University, New York, NY USA; 5 Department of Biomedical Informatics, Columbia University, New York, NY USA; 6 Department of Cancer Biology, Lerner Research Institute, Cleveland Clinic, Cleveland, OH USA; 7 Center for Theragnosis, BRI, Korea Institute of Science and Technology, Seoul, Korea; 8 Department of Biomolecular Science, University of Science and Technology, Daejeon, Korea; 9 Department of Anatomy and Cell Biology, Sungkyunkwan University, Seoul, Korea; 10 Department of Neurosurgery, Samsung Medical Center, Sungkyunkwan University, Seoul, Korea

**Keywords:** Cancer stem cells, Cell biology

Correction to: *Experimental & Molecular Medicine*

10.1038/s12276-019-0351-y published online 06 December 2019

After online publication of this article, the authors noticed an error in the Acknowledgments section.

The correct statement in this article should have read as follows.

“The biospecimens for this study were provided by the Samsung Medical Center BioBank. This research was supported by grants from the Korea Health Technology R&D Project through the Korea Health Industry Development Institute (KHIDI) funded by the Ministry of Health & Welfare, Republic of Korea (HI14C3418); the National Research Foundation funded by the Ministry of Science, ICT and Future Planning (MSIP) of Korea (NRF-2015M3C9A1044522 and NRF-2018R1C1B3001648); and the intramural research fund of Ajou University Medical Center (2018).”

The authors apologize for any inconvenience caused.

