# Supplementary material for: Secretome analysis of patient-derived GBM tumor spheres identifies midkine as a potent therapeutic target
Source: Exp Mol Med. 2019 Dec 6;51(12):147. doi: 10.1038/s12276-019-0351-y (PMC6897967; doi:10.1038/s12276-019-0351-y)
Supplement: Supplementary file 1 — Supplementary Information [file 12276_2019_351_MOESM1_ESM.pdf]

Patient GBM tumor-sphere originated secretome analysis identifies Midkine, as a potent therapeutic target.

Suji Han, Hye-Mi Shin and Jin-Ku Lee *et al.,*

Supplementary Information

Materials and Methods

Patient-derived GBM specimens and primary tumor-sphere culture

All surgical specimens were acquired from the GBM patients at the Samsung Medical Center (Seoul, Korea) in consensus with the valid Institutional Review Boards. Tumor specimens were dissociated into single cells using Liberase TM (Sigma-Aldrich), followed by percoll (Sigma) purification. Tumor cell isolates and human neural stem cells (hNPC, 101Bio) were cultured in neurobasal A media (Thermofisher) with N2 and B27 supplement (0.5X each, Thermofisher), human recombinant basic fibroblast growth factor (bFGF), epidermal growth factor (EGF) (20ng/ml each, Peprotech), Penicillin-Streptomycin and 2mM L-glutamine (Thermofisher).[1]

Secretome analysis

Proteolytic digestion of proteins, fraction of peptides and mass spectrometric analysis were similar to the method described previously.[2] The peptide samples were separated using a reversed phase Magic C18 column (75 µm) on an Agilent 1200 HPLC system with a linear gradient of 10-40% in acetonitrile containing 0.1% formic acid for 90 min (400 nL/min). The HPLC system was coupled to an LTQ-XL mass spectrometer (Thermofisher). The ESI source was operated in the positive ion mode (300-2000 m/z) with spray voltage set at 1.9 kV, capillary voltage at 30 V and the heated capillary temperature was set at 250 °C. Each scan cycle consisted of one full MS scan in profile mode followed by six data dependent

MS/MS scans with the following options: isolation width, 1.5 m/z; normalized collision energy, 25%; dynamic exclusion duration, 180s.

The acquired MS/MS spectra were compared against various databases using SEQUEST in Proteome Discoverer 1.4 (Thermofisher) against the human UniProtKB database (released in April 2014) supplemented with experimentally validated B27 and N2 contaminant sequences. The B27 and N2 contaminant sequences were mammal UniProt sequences identified earlier by LC-MS/MS analysis of B27 and N2 supplements in growth medium.[3] Two trypsin missed cleavages, fixed modification of carbamidomethylation at cysteine (+ 57.02 Da) and variable modification of oxidation at methionine (+ 15.99 Da) were allowed. Mass tolerances for MS/MS and MS were set to ± 0.5 and ± 2 Da, respectively. Peptide and protein assignment and validation (FDR >1%) were performed.

The biological functions of identified proteins were analyzed using DAVID functional classification tool (https://david.ncifcrf.gov/). The Functional networks based on the biological processes were analyzed using the ClueGO Cytoscape plugin.[4]

Immunohistochemistry and immunofluorescence analysis

Immunohistochemical analysis were performed using formalin-fixed, paraffin embedded (FFPE) tissues from GBM patient or tumor bearing xenograft. Sections were incubated with primary antibodies included andi-Midkine (Abcam), anti-pAkt, anti-pERK (Cell Signaling Technology, CST), followed by biotinylated secondary antibodies (Vector Laboratories) and treated SignalStain Boost IHC Detection Reagent (CST). Sections were then stained with 3,3-diaminobenzidine substrate (Vector Laboratories) and counterstained with H&E solution.

For immunofluorescence analysis, tissues or cells were fixed with 4% paraformaldehyde for 20 min. Tissues were further incubated in 30% sucrose overnight and then embedded in optimal cutting temperature (OCT) compound and crysectioning at 10 µm. After blocking and permeabilization with 0.5% triton X-100 and 5% normal goat or donkey serum in PBS, sections were stained with target antibodies in PBS with 0.1% bovine serum albumin (BSA),

followed by incubation with fluorescein-conjugated secondary antibodies were used for visualization. 4′, 6′-diamidine-2′-phenylindole (DAPI) staining was used to control as an intracellular localization. Images were obtained using LSM700 Confocal Laser Scanning Microscope system (Zeiss) or Operetta High Content Imaging System (PerkinElmer).

Cell proliferation assay

For evaluating short-term proliferation, 500 single cells were plated per well in a 384-well plate. Next, cells were treated with an anti-MDK antibody (Santa Cruz Biotechnology) or recombinant MDK (Sigma-Aldrich) and further incubated at 37℃ for 4 days. Cell viability was analyzed using an adenosine triphosphate (ATP) monitoring system based on firefly luciferase (ATPLite™ 1step, PerkinElmer), and luminescence was measured using an EnVIsion Mutilabel plate reader (PerkinElmer).[5] Relative cell viability was obtained by normalization to luminescence values of vehicle or control IgG treated cells.

Anti-EdU fluorescence assay was carried out according to the manufacturer's recommendation (BCK-EdU594, Sigma-Aldrich). At first, EdU solution (50 μM) was added to each well, and cells were incubated at 37℃ for 24 h. Culture medium was removed, and cells were fixed with 4% formaldehyde in PBS. Cells were washed 5 times with 3% BSA in PBS solution and permeabilized with 0.5% triton X-100 in PBS, followed by adding reaction cocktail. The nuclei were counter-stained with Hoechst 33342 solution. Fluorescence images were captured with Operetta High-Content Imaging System (PerkinElmer).

Sphere formation analysis and Limiting dilution assay (LDA)

For sphere formation assay, single cells isolated from primary tumor spheres were seeded (100 cells per well) in a 96-well plate. After 10-14 days, the tumor spheres were captured using Operetta High Content Imaging System (PerkinElmer), and the number of spheres, roundness, sphere area were analyzed using Harmony High Content Imaging and Analysis

Software (PerkinElmer). Cell aggregates of size with 25-75 percentile were selected and those of roundness less than 0.9 were excluded at the analyses. LDA was also fulfilled in 96 well plates and cells were seeded at a range of 1–200 cells per well (12 wells per group). After 10-14 days, wells without spheres were measured and analyzed. Statistically significance was analyzed using ELDA software (Walter+Eliza Hall Bioinformatics).[6]

Lentivirus production and transduction

MDK knockdown shRNA lentiviral clones (TRCN0000331210 and TRCN0000331252 for shMDK-1 and shMDK-2, respectively) and shRNA lentiviral clones for PCBP4 knockdown (TRCN0000232287 and TRCN0000232288) were purchased from Sigma-Aldrich, and a pLenti-PCBP4 expression vector was obtained from Abm. Lentiviruses were produced in 293FT cells with packaging mix (ViraPower Lentiviral Expression Systems, Thermofisher) and concentrated by ultracentrifugation. Viral titers were determined by the serial dilution method. Lentiviral particles were transduced into cells, and stable transfectants were selected by incubation with puromycin (1~2 ng/ml).

Western blot assay

GBM tumor-spheres were washed in cold PBS, followed by being harvested in passive lysis buffer (Promega) and a protease and phosphatase inhibitor cocktail added (Roche). Insoluble materials were removed by centrifugation at 13000 rpm for 5 min, 4°C. The proteins were separated by SDS-PAGE and transferred to PVDF membranes using iBlot 2 Bry Blotting System (Thermofisher). Immunoblotting was performed using antibodies against MDK, pP53 (Abcam), AKT, pAKT (Ser473), ERK, pERK (Thr202/Thy204), STAT3, pSTAT3 (Thr705), MEK, pMEK (Ser217/221), S6K, pS6K (Thr389), Chk1, pChk1 (Ser345), Chk2, pChk2 (Thr68), GAPDH, Cyclin D1, Cbl, pCbl, pγH2AX (Ser139) (all from CST), pGrb2 (Ser159) (Thermofisher), NOXO1 (Lifespan Bioscience), PCBP4 (Sigma-aldrich), beta-actin, Grb2, Cyclin A and Cyclin C (all from Santacruz Biotechnology).

Orthotopic GBM xenograft models

All animal experiments were approved by the Institutional Review Board of the SMC and performed according to the guidelines of the Animal Use and Care Committees. GBM cells were dissociated, $1X10^4$ cells per each mouse were resuspended in 5 μl of Hank's balanced salt solution (HBSS, Thermo Scientific), followed by stereotactically (2 mm left and 1 mm anterior of the bregma, 2 mm deep from the dura) injected into the brains of Balb/c nude mice (6~8-week-old female, Orient Bio Inc.). Kaplan meier survival (GraphPad Prism v5.03) and immunohistochemical analysis were performed under blinded inspection.

RNA sequencing

RNA sequencing libraries were prepared using the Illumina TruSeq RNA Library Preparation Kit v2. The sequenced reads were trimmed and mapped onto hg19 using GSNAP version 2012-12-20.[7] The resulting aligned reads were summarized into BED files using SAMtools and bedTools (bamToBed version 2.16.2).[8] The BED files were used to estimate reads per kilobase of transcript per million reads (RPKM) using the R package DEGseq.[9] Genes with low expression level across the cohort are removed. The criteria for removing genes were 1) (max(log2(rpkm +1)) – min(log2(rpkm + 1)) >= 1; 2) max(log2(rpkm + 1)) >= 1

Bioinformatical analysis

Gene Set Enrichment Analysis (GSEA) was conducted by using the Java with the Molecular Signatures Database (MSigDB; version 5.1) C2 gene set collection.[10] P values about enrichment of genes were estimated by 20,000 gene set permutations. Single sample gene set enrichment analysis (ssGSEA) was used to produce gene set activation scores of each sample using Genepattern (http://software.broadinstitute.org/cancer/software/genepattern). Method for normalizing gene expression data is rank.[11]

For identifying differentially expressed genes from gene expression values, two groups that

were consisted by control and neutralized samples were compared with using package DEGseq of R. P values were estimated by Student's t test for paired samples and accomplish multiple correction tests using the Benjamini-Hochberg method.[12]

A radar plot representing pathway enrichment analysis (GO BP on DAVID) of differentially expressed protein (DEP) data was generated using R package 'radarchart'.

For modelling genetic association of anti-MDK antibody sensitivity, we trained the standard elastic net regression (R package: glmnet_2.0-5), combining drug sensitivities and pre-selected mRNA expression as the input features.[13] Given an anti-MDK antibody and *N* GBM tumor-spheres, we used $y = (y_1, y_2, \dots y_n)$ represents drug sensitivity of *n* cells, and $x_i = (x_{i1}, \dots, x_{ip})$, $(i = 1, \dots, p)$ indicates *p* features of *i*th cell, the elastic net regression is to solve the following optimization problem:

$$min_{\beta_0,\beta}\ [\frac{1}{2N}\sum_{i=1}^{n}\left(y_i - \beta_0 - \sum_{j=1}^{p}\left(x_{ij}\beta_j\right)\right)^2 + \lambda((1-\alpha)\frac{1}{2}||\beta||_{l_2}^2 + \alpha||\beta||_{l_1})]$$

α controls the relative balance of the L1 and L2 penalty terms, while λ controls the overall penalty level of the regularized term. Similar with γ, α was optimized by 10-fold cross validations, but using all features. α was screened using 50 values with $\alpha \in [0,1]$ equally spaced. For each model fitting, we used the function *cv.glmnet*, with its optimized λ value provided from the function.

After parameter optimization, we adopt bootstrapping strategy for 100 times to obtain a robust evaluation of the predictive power of features. During each bootstrapping, we randomly select 80% of GBM tumor-spheres with 80% of the features to fit the elastic net with above optimized α. For each feature, the time of appearances (non-zero fitting coefficient) out of the 100 bootstrappings, together with the average of its non-zero weights were used as its final assessment of predictive ability.

Survival Analysis of GBM patient

We assessed the association between MDK expression and overall survival of IDH1 wild type 129 samples with data from cBioPortal (http://www.cbioportal.org). Kaplan-meier survival analysis is performed using R software version 3.4.2 (http://www.R-project.org) and statistically tested using Log-rank test. Cut-off values for optimal prediction of survival were obtained with the R software package 'maxstat' (REF : Hothorn T, Lausen B (2002) Maximally Selected Rank Statistics in R. R News 2/1: 3–5)

Protein profiling

Protein array data were obtained using Phospho Explorer Antibody Array (# PEX100, Full Moon Biosystems). The antibody array experiment was performed by Full Moon Biosystems, according to their established protocols.[14] In brief, the slides were scanned on an Axon GenePix array scanner, and the images were analysed with GenePix Pro 6.0 (Molecular Devices). The fluorescence signal of each antibody was obtained.

Dichlorodihydrofluorescein diacetate (DCF-DA) assay

DCFDA cellular ROS detection assay kit (Abcam) was used to measure the redox status of a cell as manufacturer's instructions.[15] In brief, cells were stained with DCFDA for 30 min, followed by washing in a phenol red free washing buffer. After incubation with desired agents, the fluorescence (Ex/Em=485/535 nm) was measured using EnVIsion Mutilabel plate reader (PerkinElmer). The fluorescence images were captured and analyzed using Operetta High Content Imaging System (PerkinElmer).

DNA damage analysis

P-γH2AX, a DNA double strand break (DSB) marker, was detected using OxiSelect DNA DSB staining kit (# STA-321, Cell biolabs) as manufacturer's instructions. The alkaline single cell gel electrophoresis to detect DNA damages were performed by OxiSlelect Comet Assay Kit (# STA-355, Cell biolabs).[16] The fluorescence images were obtained using LSM700

Confocal Laser Scanning Microscope system (Zeiss).

Cell cycle analysis

For analyzing cell cycle, single cell dissociates from GBM tumor-spheres were fixed with 100% ethanol and incubated at 4℃ overnight. Cells were stained with propidium iodide (PI, (Sigma-Aldrich) and analyzed using Flow Cytometry (FACS Aria, BD Biosciences). The flow cytometry data were interpreted with Flow Jo software (ver. 7.6, Treestar Inc).

Apoptosis assay

For Annexin V staining, single cells were washed, followed by resuspended in a Annexin V Binding Buffer (BD Pharmingen). Cells were then stained with Annexin V-APC solution (eBioscience). After adding PI (Sigma-Aldrich), Annexin V-PI positive cells were measured using FACS Aria (BD Bioscience) and data were interpreted with Flow Jo software (ver. 7.6, Treestar Inc). Caspase 3/7 activities were measured using ApoTox-Glo Triplex assay (#G6320, Promega) or CellEvent™ Caspase-3/7 Green Detection Reagent (Thermofisher) as manufacturer's instructions. The luminescence or fluorescence intensities were obtained using EnVIsion Mutilabel plate reader (PerkinElmer). The apoptosis array analysis was performed using the Proteome Profiler Human Apoptosis Array kit (ARY009, R & D Biosystems) according to the manufacturer's instruction and array image were analyzed using the Image J software (https://imagej.nih.gov/ij/index.html).

Statistical analysis

Data were presented as means ± standard deviations (SDs). P values were obtained using two-tailed unpaired t-test (GraphPad Prism v.5.03). Statistical significance was displayed where *p <0.05, **p<0.01, and ***p<0.001.

References

1 Lee, J. *et al.* Tumor stem cells derived from glioblastomas cultured in bFGF and EGF more closely mirror the phenotype and genotype of primary tumors than do serum-cultured cell lines. *Cancer Cell* **9**, 391-403, doi:10.1016/j.ccr.2006.03.030 (2006).

2 Shaheen, K. Y., Abdel-Mageed, A. I., Safwat, E. & AlBreedy, A. M. The value of serum midkine level in diagnosis of hepatocellular carcinoma. *Int J Hepatol* **2015**, 146389, doi:10.1155/2015/146389 (2015).

3 Shin, J. *et al.* Use of composite protein database including search result sequences for mass spectrometric analysis of cell secretome. *PLoS One* **10**, e0121692, doi:10.1371/journal.pone.0121692 (2015).

4 Bindea, G. *et al.* ClueGO: a Cytoscape plug-in to decipher functionally grouped gene ontology and pathway annotation networks. *Bioinformatics* **25**, 1091-1093, doi:10.1093/bioinformatics/btp101 (2009).

5 Lee, J. K. *et al.* Spatiotemporal genomic architecture informs precision oncology in glioblastoma. *Nature genetics* **49**, 594-599, doi:10.1038/ng.3806 (2017).

6 Hu, Y. & Smyth, G. K. ELDA: extreme limiting dilution analysis for comparing depleted and enriched populations in stem cell and other assays. *J Immunol Methods* **347**, 70-78, doi:10.1016/j.jim.2009.06.008 (2009).

7 Wu, T. D. & Nacu, S. Fast and SNP-tolerant detection of complex variants and splicing in short reads. *Bioinformatics* **26**, 873-881, doi:10.1093/bioinformatics/btq057 (2010).

8 Quinlan, A. R. & Hall, I. M. BEDTools: a flexible suite of utilities for comparing genomic features. *Bioinformatics* **26**, 841-842, doi:10.1093/bioinformatics/btq033 (2010).

9 Wang, L., Feng, Z., Wang, X., Wang, X. & Zhang, X. DEGseq: an R package for identifying differentially expressed genes from RNA-seq data. *Bioinformatics* **26**, 136-138, doi:10.1093/bioinformatics/btp612 (2010).

10 Subramanian, A. *et al.* Gene set enrichment analysis: a knowledge-based approach for interpreting genome-wide expression profiles. *Proc Natl Acad Sci U S A* **102**, 15545-15550, doi:10.1073/pnas.0506580102 (2005).

11 Verhaak, R. G. *et al.* Prognostically relevant gene signatures of high-grade serous ovarian carcinoma. *J Clin Invest* **123**, 517-525, doi:10.1172/JCI65833 (2013).

12 Benjamini, Y., Drai, D., Elmer, G., Kafkafi, N. & Golani, I. Controlling the false discovery rate in behavior genetics research. *Behav Brain Res* **125**, 279-284 (2001).

13 Wang, J., Kribelbauer, J. & Rabadan, R. Network propagation reveals novel genetic features predicting drug response of cancer cell lines. *Current Bioinformatics* **11**, 8, doi:10.2174/1574893611666160125222144. (2016).

14 Bosma, M. *et al.* FNDC4 acts as an anti-inflammatory factor on macrophages and improves colitis in mice. *Nat Commun* **7**, 11314, doi:10.1038/ncomms11314 (2016).

15 Eruslanov, E. & Kusmartsev, S. Identification of ROS using oxidized DCFDA and flow-cytometry. *Methods Mol Biol* **594**, 57-72, doi:10.1007/978-1-60761-411-1_4 (2010).

16 Tyagi, A. *et al.* Resveratrol selectively induces DNA Damage, independent of Smad4

expression, in its efficacy against human head and neck squamous cell carcinoma. *Clin Cancer Res* **17**, 5402-5411, doi:10.1158/1078-0432.CCR-11-1072 (2011).

Supplementary Figures and Tables

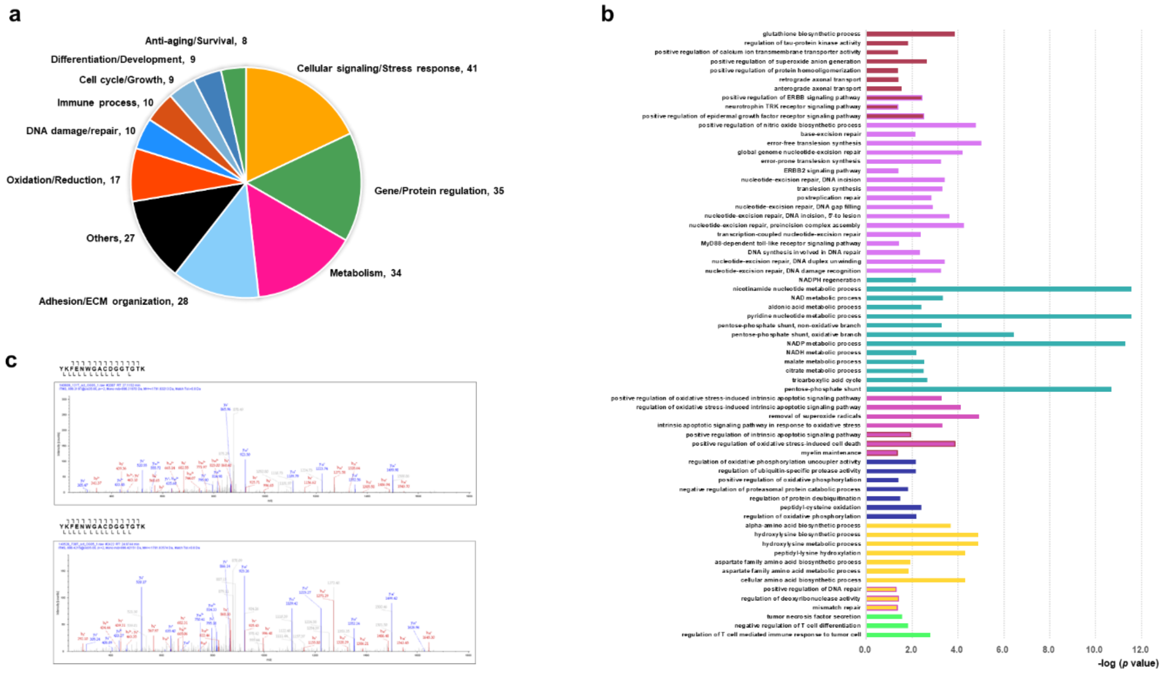

Supplementary Figure 1. (a) A pie chart demonstrated the number of enriched pathways for indicated functional categories, based on Geno Ontology (GO) using DAVID functional classification tool in identified total proteins. **(b)** A bar chart represented enriched key pathways shown on Figure 1c. **(c)** MS/MS peaks of peptides for MDK protein analyzed conditioned media from NCI131 (up) and N783 (bottom) cells.

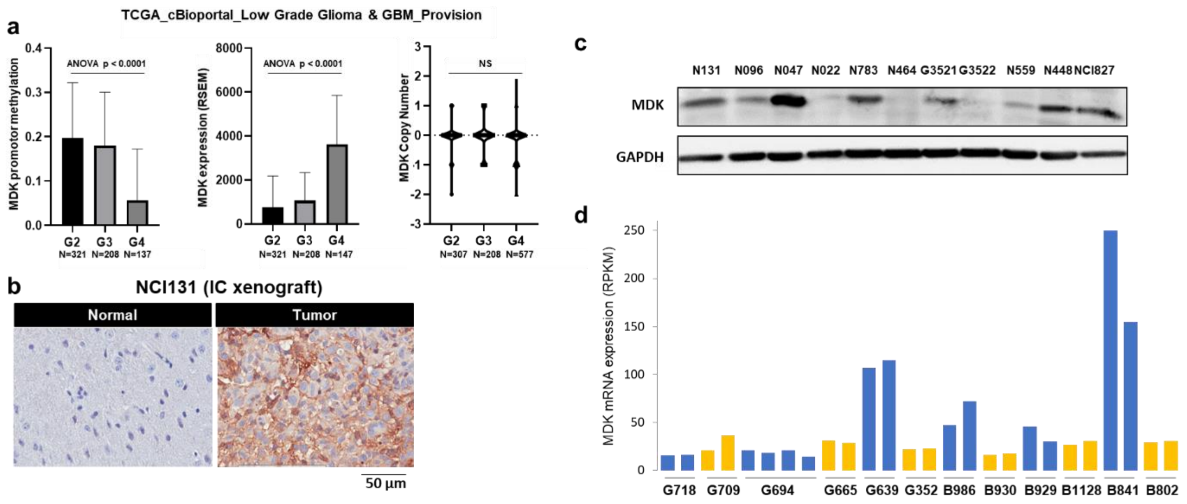

Supplementary Figure 2. (a) The methylation, mRNA expression and copy number status in gliomas (TCGA low grade glioma and glioblastoma_provision dataset). **(b)** Immunohistochemical analyses for MDK expression in paraffin embedded xenograft tissue from NCI131 tumorsphere. Bar represents 50 µm. **(c)** Western blot analysis of MDK expressions in 11 different patient-derived GBM tumorspheres. Data were representatives of three independent experiments. **(d)** MDK mRNA expression levels of multisector GBM samples.

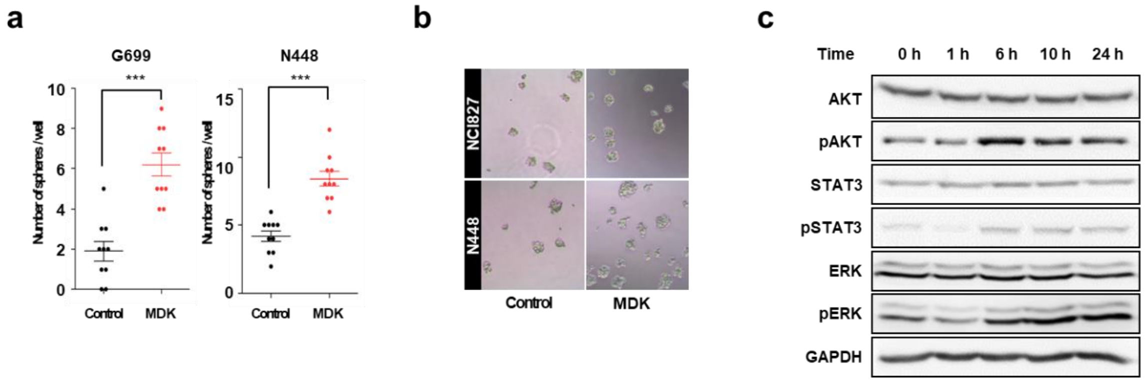

Supplementary Figure 3. (a) GBM tumor-spheres were dissociated into single cells and 100 cells/well were seeded in an 96 well plate. The number of spheres per well on the treatment of vehicle (black) or recombinant MDK (10 ng/ml, 10 days) in G699 (left) and N448 (right) cells were demonstrated as vertical scattered plots (n=10 per group). **(b)** Representative sphere images shown in (a) were presented. **(c)** Immunoblots for indicated proteins on the treatment of recombinant MDK (10 ng/ml) for indicated time points were demonstrated. Data were representatives of three independent experiments. GAPDH was used as a loading control.

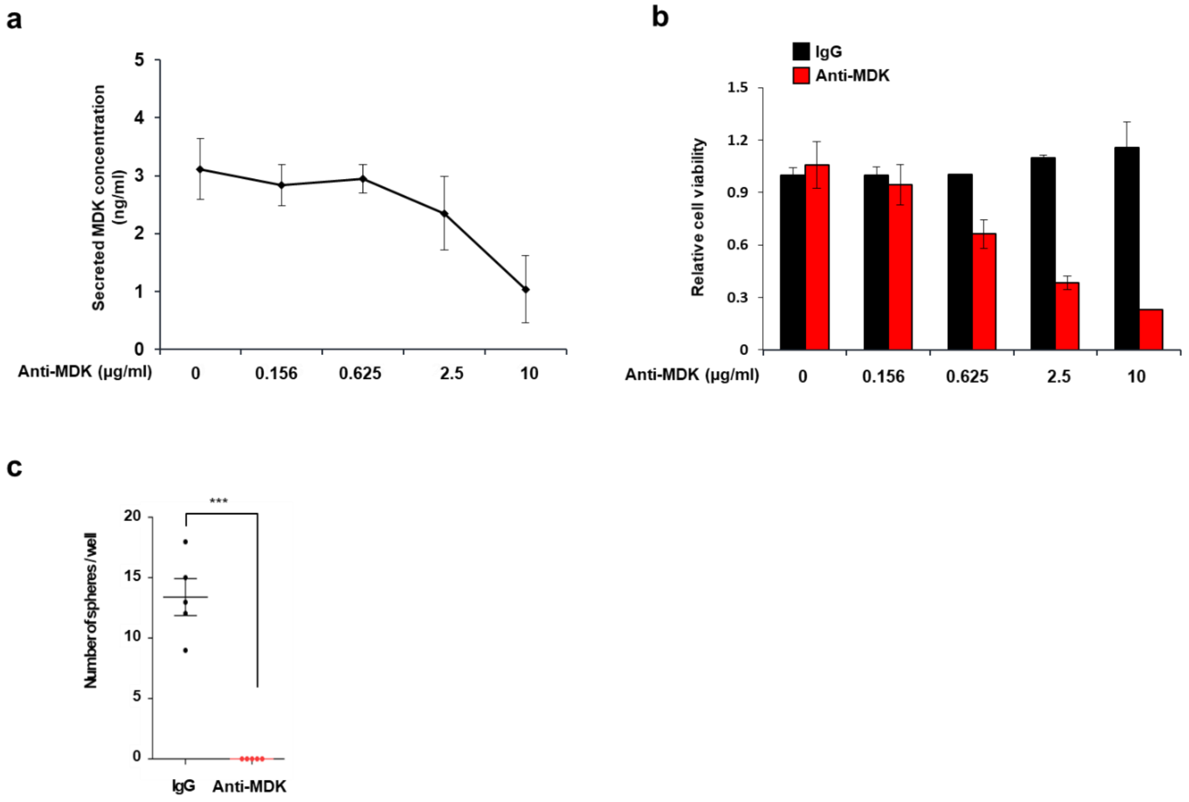

Supplementary Figure 4. (a) The protein concentrations of MDK (ng/ml) in a conditioned medium from NCI827 cell incubated with an anti-MDK antibody at indicated doses for 2 h were measured using ELISA and demonstrated as a line graph. **(b)** The relative cell viability of control IgG (black bars) and an anti-MDK antibody (red bars) treated NCI827 cells at indicated doses for 4 days were measured using EZ-cytox cell viability assay kit and demonstrated as a bar graph. **(c)** Single cells from NCI827 tumor-spheres were plated on a 96 well plate (100 cells/well). Number of spheres per well (n=5 per group) on the treatment of control IgG and an anti-MDK antibody (10 μg/ml for 10 days) were demonstrated as a vertical scattered plot.

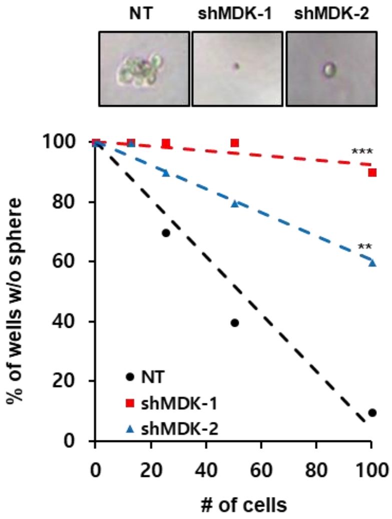

Supplementary Figure 5. Limiting dilution assay (LDA) in NCI131 cells transfected with NT (black) or two different shMDK constructs (red and blue), and representative sphere images were demonstrated. Statistical significance was obtained using ELDA (http://bioinf.wehi.edu.au/software/elda/; see also Supplementary Table 2.

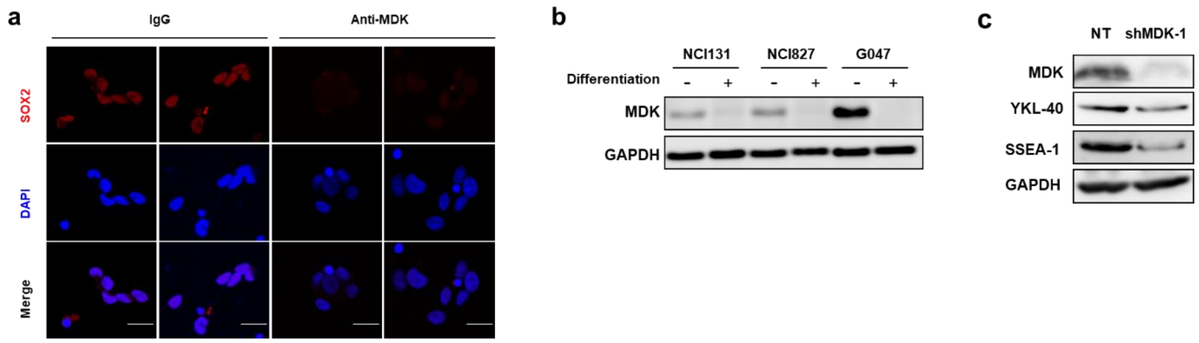

Supplementary Figure 6. (a) Immunofluorescence analysis for MDK (green), SOX2 (red) and/or DAPI (blue) on fresh frozen specimens from GBM tumor (G047) and the matched normal tissue. Scale bars indicate 100 µm. Data were representatives of three independent experiments. **(b)** Immunoblots for MDK expression in indicated GBM tumor-spheres with or without FBS-induced differentiation condition. Data were representatives of three independent experiments. **(c)** Western blot analyses for indicated proteins on the treatment of control NT or an shMDK-1 were demonstrated. Data were representatives of three independent experiments. GAPDH was used as a loading control.

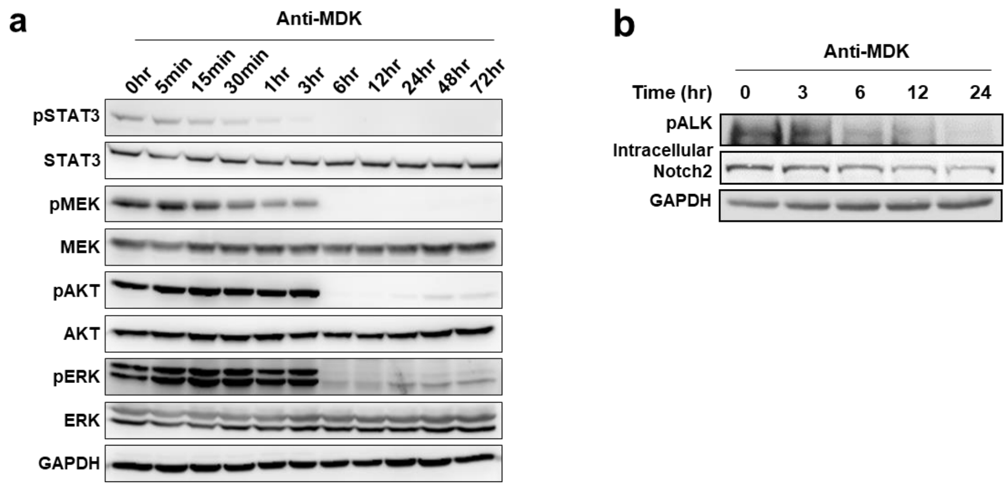

Supplementary Figure 7. (a and **b**) Immunoblots for indicated proteins on the treatment of an anti-MDK antibody at the indicated time points. GAPDH was used as a loading control.

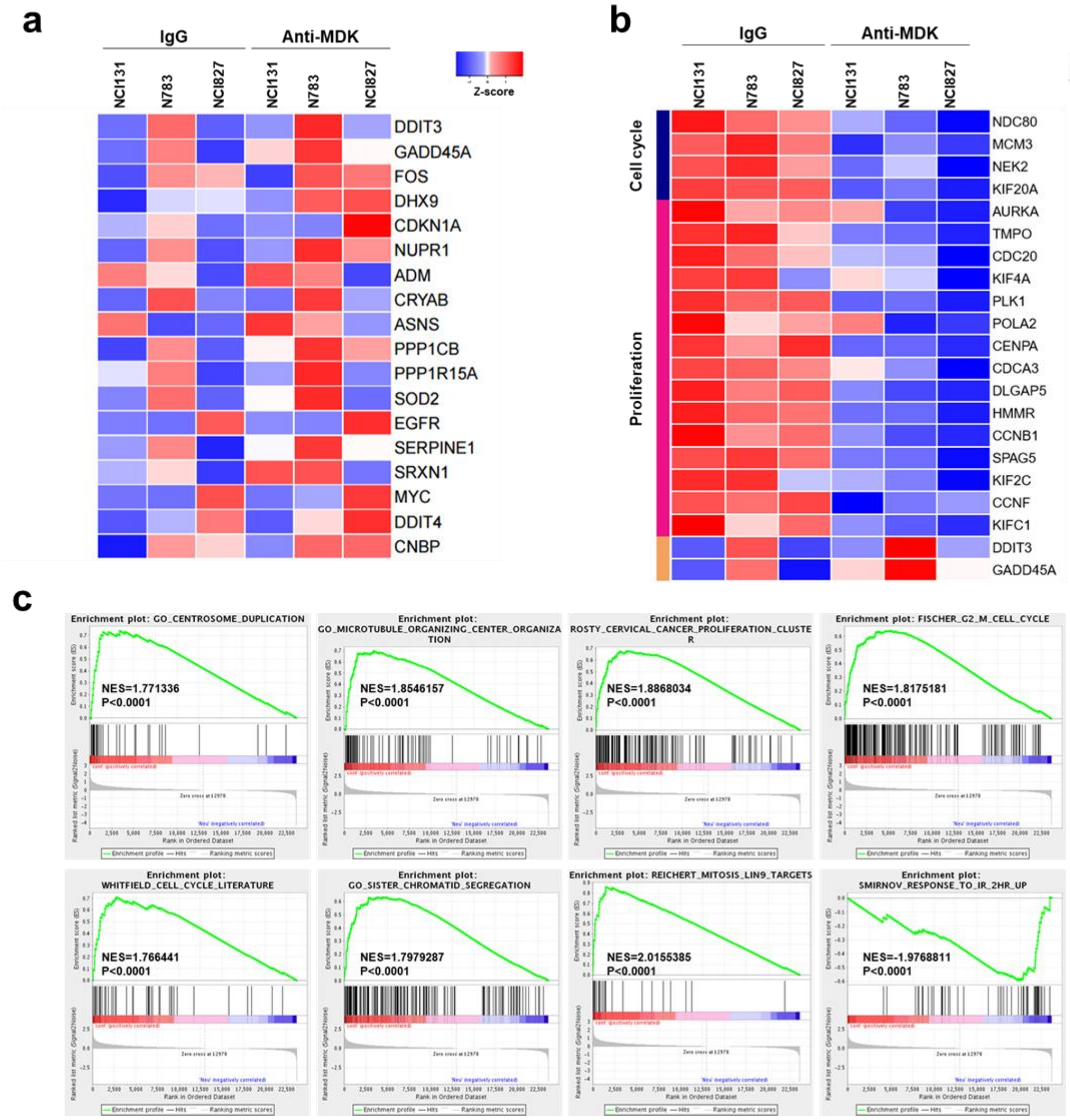

Supplementary Figure 8. Heatmaps for differentially expressed genes (DEG) associated with **(a)** oxidative stress-response or redox signaling, **(b)** cell cycle, proliferation and apoptosis processes between IgG and anti-MDK antibody (20 µg/ml, 12 h) treated NCI131, N783 and NCI827 cells were demonstrated. **(c)** GSEA results of control and MDK inhibition group of cells.

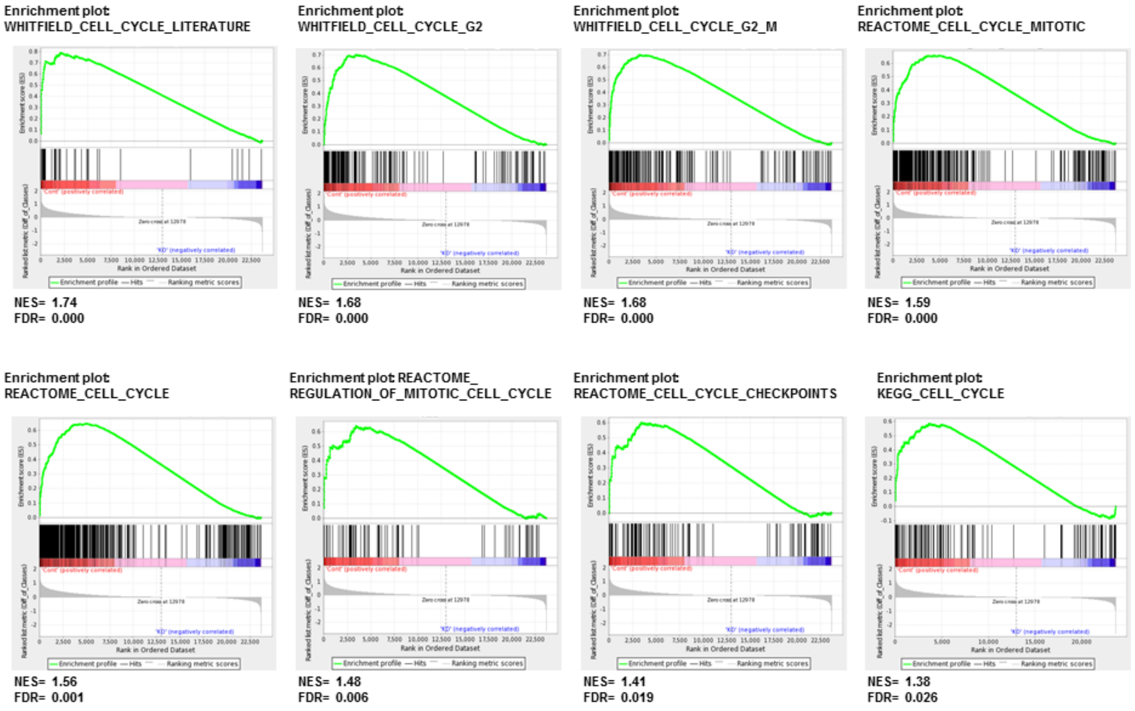

Supplementary Figure 9. Enrichment plots for indicated gene sets on the treatment of an anti-MDK antibody (20 µg/ml, 12 h), compared to IgG control.

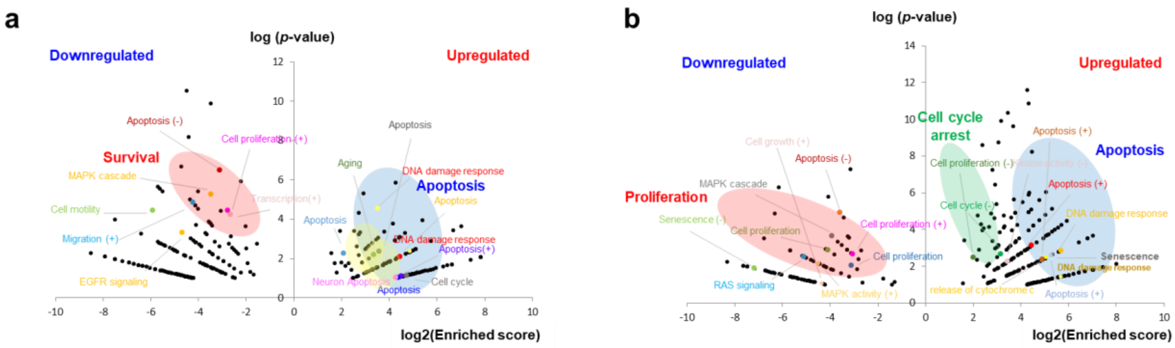

Supplementary Figure 10. Volcano plots represent the enrichment score (x-axis) and significance (y-axis) of enriched pathways (GO BP on DAVID) upregulated (right, red) and downregulated (left, blue) on the treatment of an anti-MDK antibody (20 µg/ml, 12 h) using protein array data in **(a)** NCI827 and **(b)** NCI131 cells.

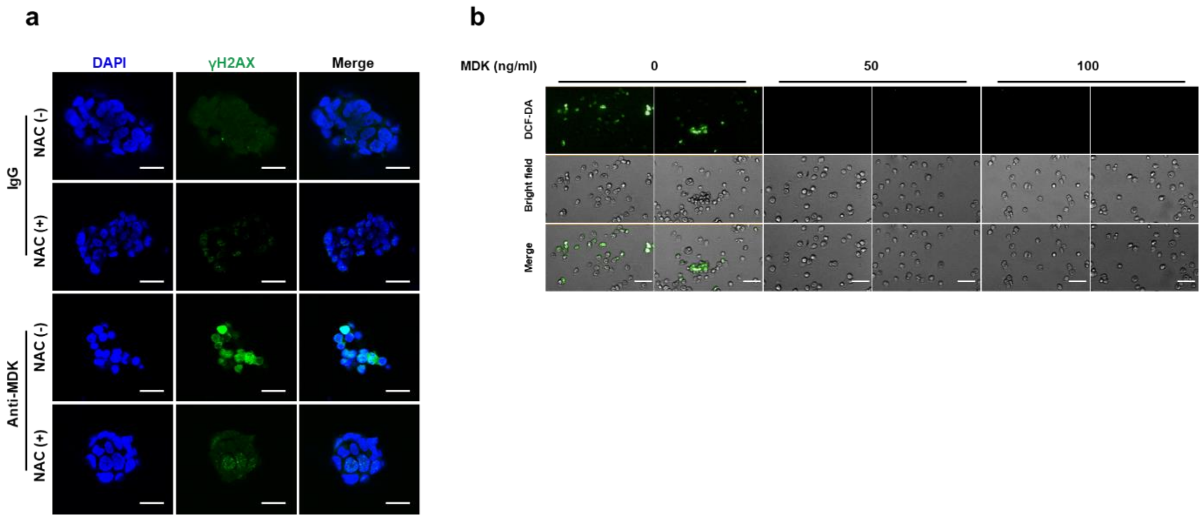

Supplementary Figure 11. (a) Immunofluorescence images for DAPI (blue) and/or phosphor-γ H2AX (green) on the treatment of control IgG and an anti-MDK antibody (5 μg/ml, 5 h) with or without N-acetylcysteine (NAC, 100 μM) in GBM tumor-spheres. Scale bars indicate 20 μm. **(b)** Green fluorescence and/or brightfield images on DCF-DA stained NCI827 cells upon the treatment of recombinant MDK as indicated doses for 6 h were demonstrated. Bars indicate 50 μm.

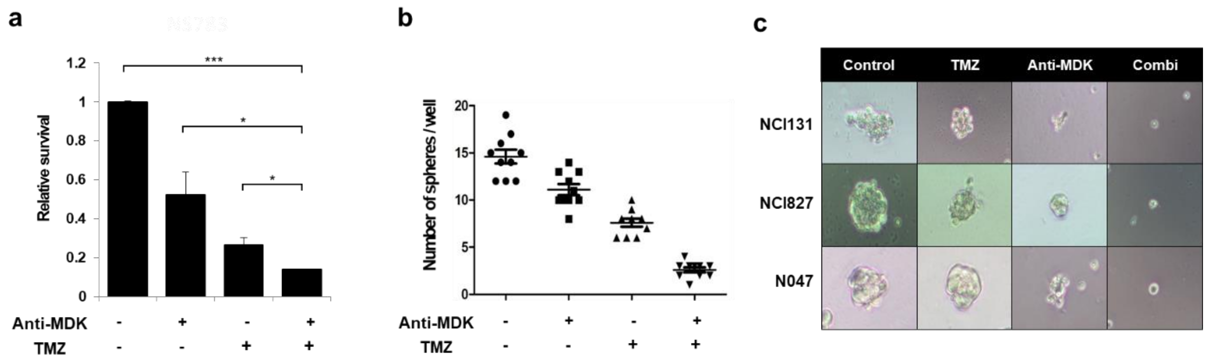

Supplementary Figure 12. Relative cell survival on the treatment of IgG control or an anti-MDK antibody (1 µg/ml) with or without temozolomide (TMZ, 100 µM) in N783 cell was demonstrated as a **(a)** bar graph. Each bar was presented as mean ± SD. P values were obtained using paired t-test (*p<0.05 and ***p<0.001). **(b)** Number of spheres per well (n=10 per group) on the groups shown at (A) was presented as a vertical scattered plot, and the **(c)** representative tumor-sphere images in NCI131, NCI827 and N047 cells were displayed.

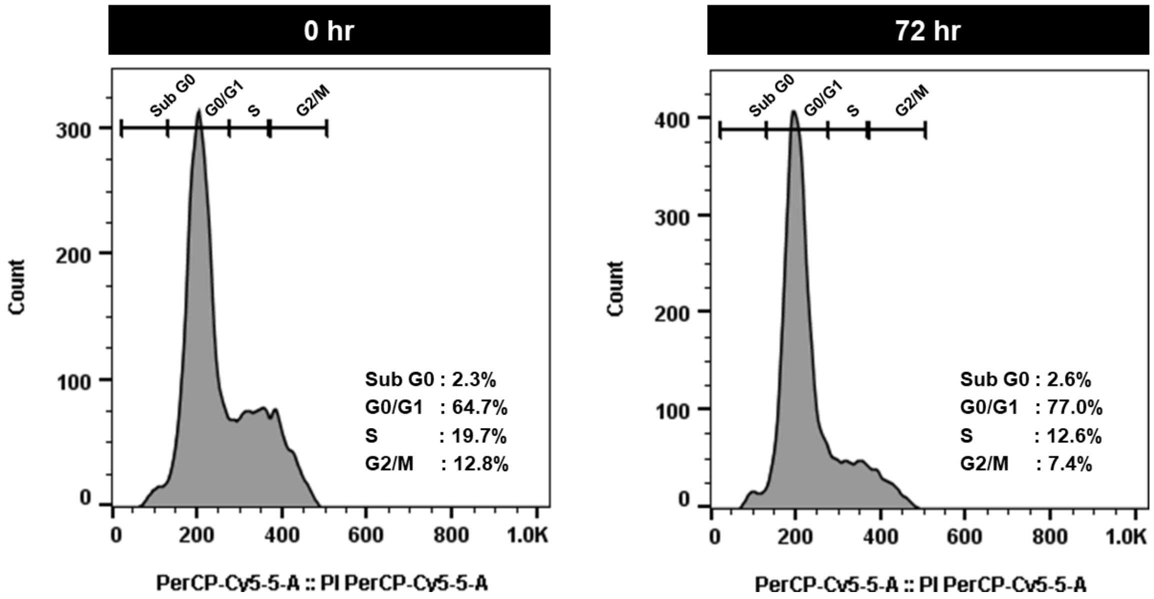

Supplementary Figure 13. Flow cytometric cell cycle analyses on control or anti-MDK (1 μg/ml) treatment NCI131 cells at 72 h were demonstrated.

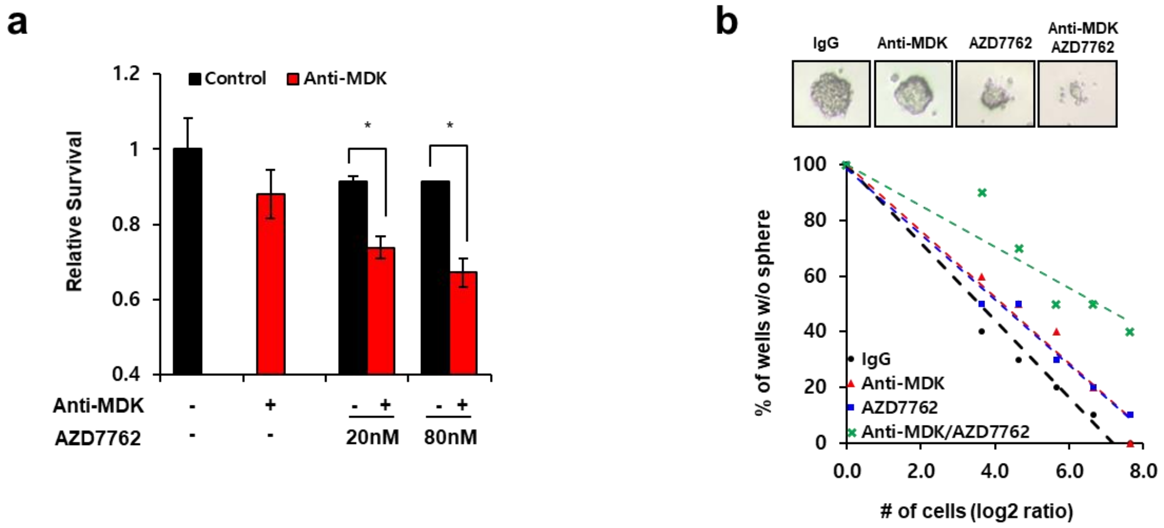

Supplementary Figure 14. (a) The relative survival of control (black bar) and anti-MDK antibody (5 µg/ml, red bar) with or without AZD7762 (Selleckchem) was demonstrated as bar graphs. Statistical significance was calculated using student t-test. (*p<0.5) **(b)** An LDA plot and representative sphere images of control and treatment of anti-MDK with or without AZD7762 were demonstrated.

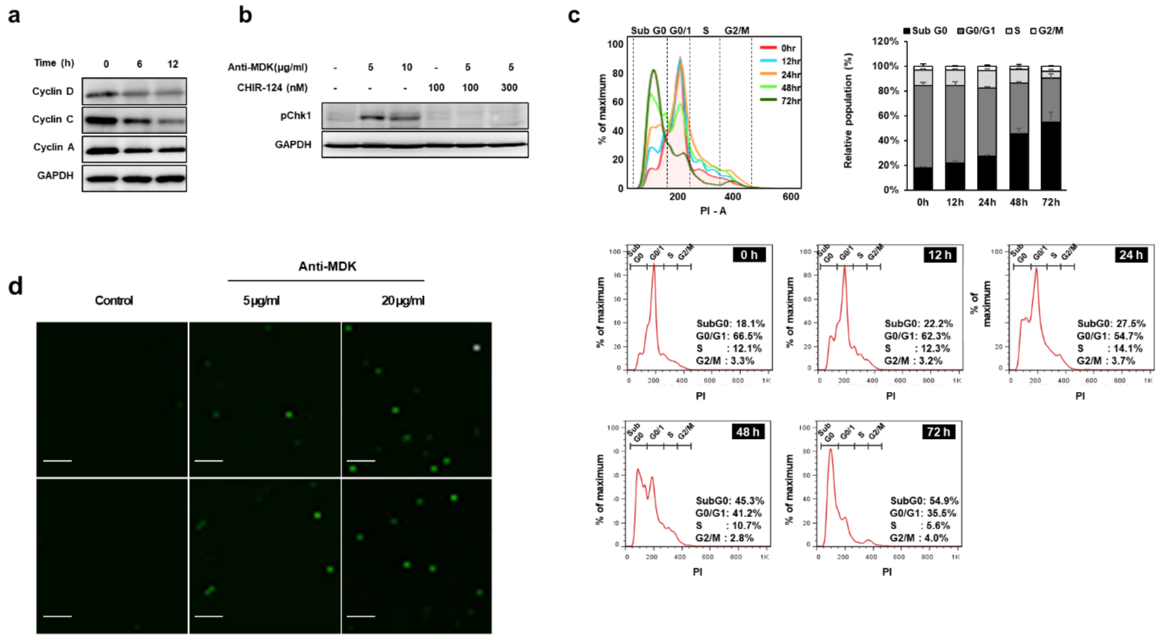

Supplementary Figure 15. (a) Western blot analyses for indicated proteins on anti-MDK treated NCI827 cells for indicated time were demonstrated. **(b)** Western blots for indicated proteins on the treatment of an anti-MDK antibody and/or CHIR-124 as indicated doses at 12 h were displayed. Data were representatives for three independent experiments. GAPDH was used as a loading control. **(c)** Flow cytometry-based cell cycle analysis of NCI827 cells on the treatment of an anti-MDK antibody (20 μg/ml) for indicated time (left and bottom), and their quantification was demonstrated (right). **(d)** Immunofluorescence images for detecting caspase 3/7 positive cells on control or anti-MDK antibody treated NCI827 cells as indicated doses were displayed. Data were representatives for three independent experiments.

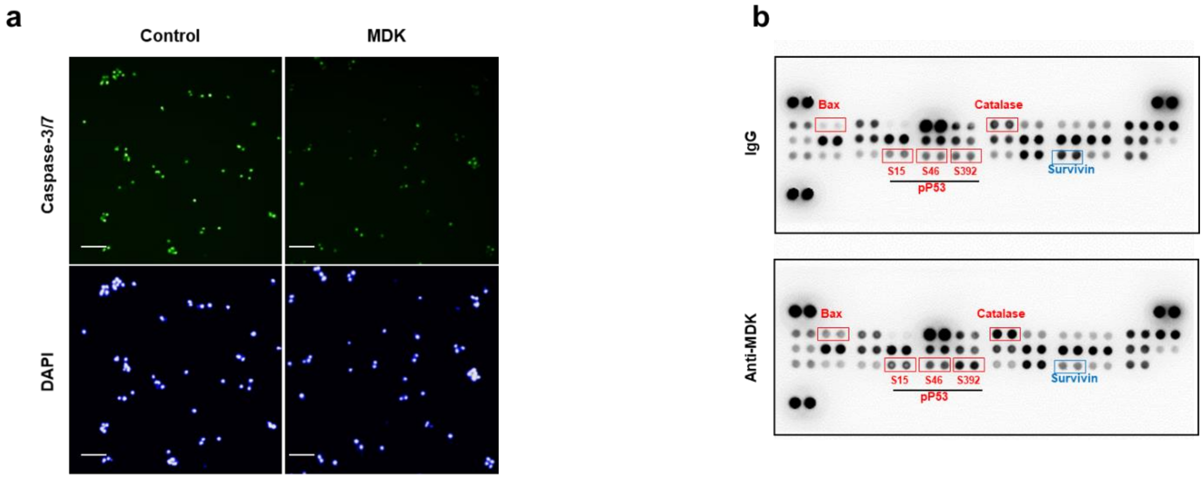

Supplementary Figure 16. (a) Immunofluorescence analyses for caspase 3/7 induced by TBHP (50 µM) on control or recombinant MDK (10 ng/ml) treated cells were demonstrated. Scale bars indicate 50 µm. Data were representatives for three independent experiments. **(b)** Apoptosis array was demonstrated on control IgG or an anti-MDK antibody treated (5 µg/ml, 12h) NCI827 cells.

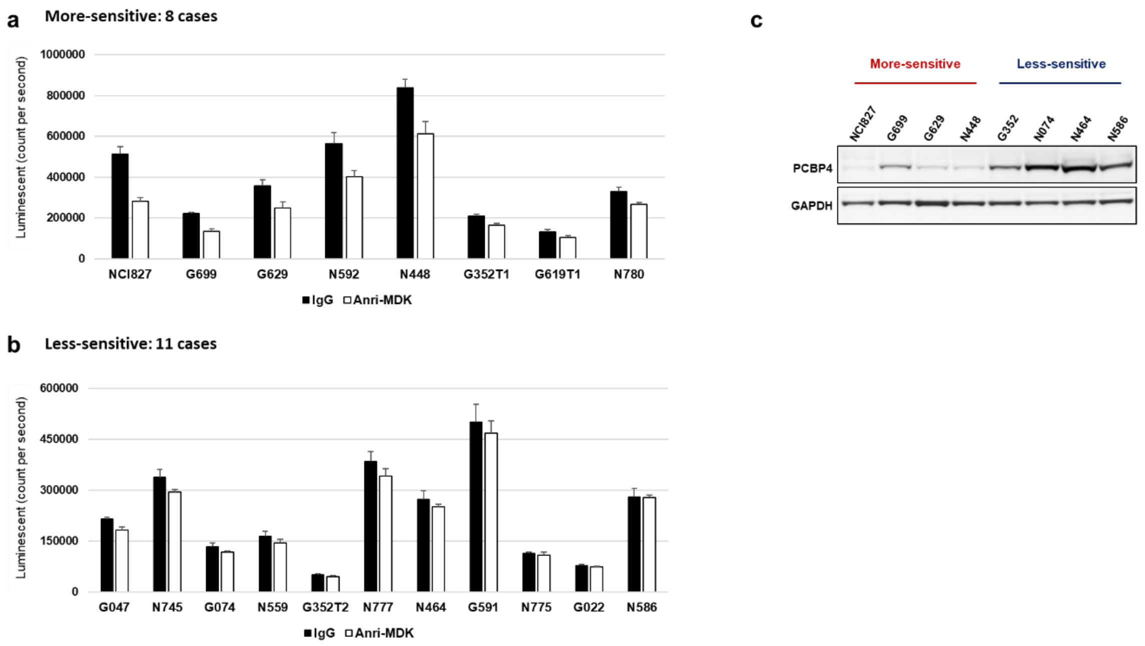

Supplementary Figure 17. The cell viabilities based on cellular ATP (luminescent) level measured by ATPlite on the treatment of control IgG (black bars) or an anti-MDK antibody (5 µg/ml, 4 days, white bars) in more-sensitive (n=8, **a**) and less sensitive (n=11, **b**) cells were demonstrated by bar graphs. **(c)** Immunoblots for PCBP4 expression level in representative cells with "more sensitive" and "less sensitive" to anti-MDK treatment (n=4 for each group). Data were representatives of three independent experiments. GAPDH was used as a loading control.

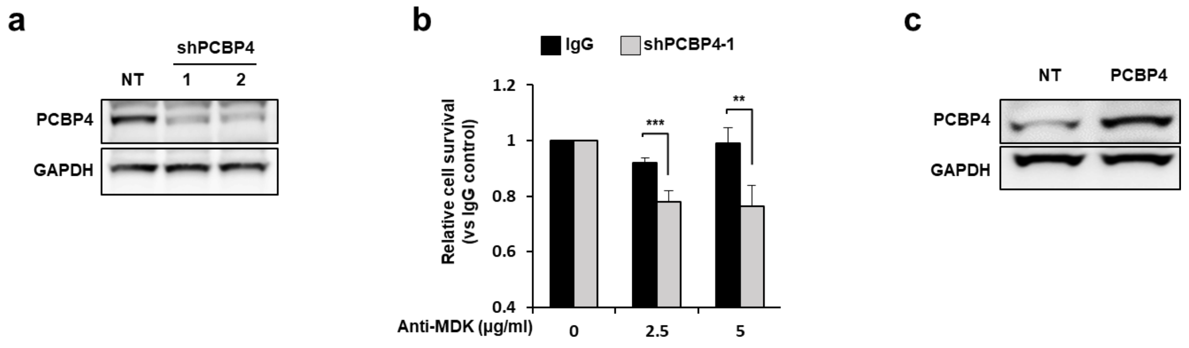

Supplementary Figure 18. Western blot analyses for PCBP4 expressions on cells transfected with **(a)** NT or two shRNAs targeting *PCBP4* gene, and **(c)** ectopic expression of PCBP4 were demonstrated. GAPDH was used as a loading control. Data were representatives of three independent experiments. **(b)** Relative survival on an anti-MDK treatment at indicated doses (4 days), normalized to cell viability of IgG control group in N464 cells transfected with NT or shPCBP4 constructs

Supplementary Table 1. Summary of identified secretome in conditioned media from NCI131 and N783 cells.

Sample ID	Identified protein	Classical secretion[a]	Non-classical secretion[b]	Membrane protein[c]	Others[d]	Percentage of predicted secreted proteins
NCI131	471	125	130	6	210	54.1
N783	389	77	128	3	181	52.7
Total	630	152	194	9	275	54.9

[a]Proteins predicted by the **SignalP** program to be secreted via the classical secretion pathway (SignalP 0.45 D-cutoff for noTM networks or 0.5 D-cutoff for TM networks).
[b]Proteins predicted to be secreted by the nonclassical secretion pathway using **SignalP** and **SecretomeP** (SignalP < D-cutoff and SecretomeP N-N score ≥ 0.50).
[c]Proteins predicted by the **TMHMM** to form integral membrane proteins that were not predicted to be secreted via the classical or nonclassical secretion pathways.
[d]Proteins that could not be classified as classical secreted, nonclassical secreted, integral membrane proteins or unspecified.

Supplementary Table 2. List of identified proteins in secretome analysis

Accession	Names	Length	NCI131	N783	Subcellular localization category	predicted secretion	SignalP	SecretomeP	TMHMM
Q04446	GBE1	702		O	Multiple location	others	N	0.455	N
P61604	HSPE1	102		O	Secretion	nonclassical secretion	N	0.57	N
Q9NRX4	IPT1 PHP14 CGI-202 HSPC1	125	O	O	Cytoplasmic	others	N	0.32	N
P62258	YWHAE	255	O	O	Cytoplasmic	others	N	0.33	N
P61981	YWHAG	247	O	O	Cytoplasmic	others	N	0.29	N
P63104	YWHAZ	245	O	O	Cytoplasmic	others	N	0.252	N
Q16698	DECR1 DECR SDR18C1	335	O		Secretion	nonclassical secretion	N	0.621	N
P55036	PSMD4 MCB1	377		O	Multiple location	others	N	0.22	N
Q16401	PSMD5 KIAA0072	504		O	Secretion	nonclassical secretion	N	0.558	N
O43598	DNPH1 C6orf108 RCL	174		O	Secretion	nonclassical secretion	N	0.582	N
O95861	BPNT1	308	O		Secretion	nonclassical secretion	N	0.682	N
P31937	HIBADH	336	O		Secretion	nonclassical secretion	N	0.61	N
P42765	ACAA2	397	O		Organellar	others	N	0.403	N
P46783	RPS10	165		O	Secretion	nonclassical secretion	N	0.659	N
P62280	RPS11	158		O	Secretion	nonclassical secretion	N	0.754	N
P25398	RPS12	132	O	O	Secretion	nonclassical secretion	N	0.661	N
P62249	RPS16	146	O		Secretion	nonclassical secretion	N	0.735	N
P63220	RPS21	83		O	Secretion	nonclassical secretion	N	0.709	N
P61247	RPS3A FTE1 MFTL	264		O	Secretion	nonclassical secretion	N	0.68	N
P62701	RPS4X CCG2 RPS4 SCAR	263		O	Secretion	nonclassical secretion	N	0.606	N
P46782	RPS5	204		O	Secretion	nonclassical secretion	N	0.78	N
P62241	RPS8 OK/SW-cl.83	208		O	Multiple location	others	N	0.417	N
P08865	RPSA LAMBR LAMR1	295	O	O	Secretion	nonclassical secretion	N	0.62	N
Q9BRK5	SDF4 CAB45 PSEC0034	362	O	O	Secretion	classical secretion	Y	0.591	Y
P08195	SLC3A2 MDU1	630		O	Secretion	nonclassical secretion	N	0.644	Y
P49189	LDH9A1 ALDH4 ALDH7 ALDI	494		O	Cytoplasmic	others	N	0.49	N
P10809	HSPD1 HSP60	573	O	O	Organellar	others	N	0.289	N
P05388	RPLP0	317	O	O	Multiple location	others	N	0.15	N
P05386	RPLP1 RRP1	114	O		Cytoplasmic	others	N	0.406	N
P05387	RPLP2 D11S2243E RPP2	115	O	O	Multiple location	others	N	0.265	N
P62906	RPL10A NEDD6	217		O	Secretion	nonclassical secretion	N	0.637	N
P30050	RPL12	165	O	O	Secretion	nonclassical secretion	N	0.865	N
P84098	RPL19	196		O	Multiple location	others	N	0.301	N
P62829	RPL23	140		O	Secretion	nonclassical secretion	N	0.825	N
P39023	RPL3 OK/SW-cl.32	403		O	Secretion	nonclassical secretion	N	0.537	N
P63173	RPL38	70		O	Secretion	nonclassical secretion	N	0.773	N
P36578	RPL4 RPL1	427		O	Multiple location	others	N	0.24	N
P46777	RPL5 MSTP030	297	O	O	Multiple location	others	N	0.413	N
P62424	RPL7A SURF-3 SURF3	266		O	Multiple location	others	N	0.423	N
P52209	PGD PGDH	483	O	O	Cytoplasmic	others	N	0.426	N
O95336	PGLS	258		O	Secretion	nonclassical secretion	N	0.87	N
P08253	MMP2 CLG4A	660	O	O	Secretion	classical secretion	Y	0.514	N
P11021	HSPA5 GRP78	654	O	O	Secretion	classical secretion	Y	0.745	N
Q9BWD1	ACAT2 ACTL	397	O	O	Secretion	nonclassical secretion	N	0.726	N
Q13510	ASAH1 ASAH HSD-33 HSD3	395	O		Secretion	classical secretion	Y	0.846	N
P39687	32A C15orf1 LANP MAPM PH	249	O	O	Multiple location	others	N	0.098	N
Q9BTT0	ANP32E	268	O	O	Multiple location	others	N	0.128	N
Q99798	ACO2	780	O	O	Organellar	others	N	0.413	N
P60709	ACTB	375	O	O	Cytoplasmic	others	N	0.498	N
O96019	CTL6A BAF53 BAF53A INO8	429	O	O	Nucleus	others	N	0.479	N
P61160	ACTR2 ARP2	394	O		Cytoplasmic	others	N	0.311	N
Q92747	ARPC1A SOP2L	370	O	O	Cytoplasmic	others	N	0.29	N
O15144	ARPC2 ARC34 PRO2446	300	O		Cytoplasmic	others	N	0.36	N
P61158	ACTR3 ARP3	418	O		Cytoplasmic	others	N	0.443	N
Q6VMQ6	ATF7IP MCAF MCAF1	1270		O	Nucleus	others	N	0.344	N
P13798	EH D3F15S2 D3S48E DNF1	732	O		Secretion	nonclassical secretion	N	0.547	N
P07108	DBI	87		O	Secretion	nonclassical secretion	N	0.546	N
P46108	CRK	304		O	Multiple location	others	N	0.467	N
P23526	AHCY SAHH	432	O	O	Secretion	nonclassical secretion	N	0.507	N
P54819	AK2 ADK2	239	O	O	Secretion	nonclassical secretion	N	0.823	N
P30520	ADSS ADSS2	456	O		Cytoplasmic	others	N	0.453	N
Q01518	CAP1 CAP	475	O	O	Membrane	others	N	0.429	N
Q8IUX7	AEBP1 ACLP	1158	O		Secretion	classical secretion	Y	0.118	N
Q9UKK9	NUDT5 NUDIX5 HSPC115	219	O	O	Nucleus	others	N	0.188	N
O43488	AKR7A2 AFAR AFAR1 AKR7	359	O		Secretion	nonclassical secretion	N	0.734	N
O00468	AGRN AGRIN	2067	O	O	Secretion	classical secretion	Y	0.293	N
P14550	AKR1A1 ALDR1 ALR	325	O	O	Multiple location	others	N	0.49	N
P11766	ADH5 ADHX FDH	374	O	O	Cytoplasmic	others	N	0.369	N
P15121	AKR1B1 ALDR1	316	O		Cytoplasmic	others	N	0.395	N
P01023	A2M CPAMD5 FWP007	1474	O	O	Secretion	classical secretion	Y	0.596	N
P12814	ACTN1	892	O	O	Cytoplasmic	others	N	0.431	N
O43707	ACTN4	911	O	O	Multiple location	others	N	0.418	N
O43768	ENSA	121	O		Secretion	nonclassical secretion	N	0.825	N
P06733	ENO1 ENO1L1 MBPB1 MPB	434	O	O	Secretion	nonclassical secretion	N	0.536	N
Q16706	MAN2A1 MANA2	1144	O		Secretion	nonclassical secretion	N	0.661	Y
P54802	NAGLU UFHSD1	743	O		Secretion	classical secretion	Y	0.583	N
Q9H4A4	RNPEP APB	650	O		Secretion	nonclassical secretion	N	0.554	N
P05067	APP A4 AD1	770	O	O	Secretion	classical secretion	Y	0.441	Y
Q06481	APLP2 APPL2	763	O		Secretion	classical secretion	Y	0.507	Y
P01019	AGT SERPINA8	485		O	Secretion	classical secretion	Y	0.751	N
P04083	ANXA1 ANX1 LPC1	346	O	O	Secretion	nonclassical secretion	N	0.511	N
P08758	ANXA5 ANX5 ENX2 PP4	320	O		Secretion	nonclassical secretion	N	0.55	N
P08133	ANXA6 ANX6	673	O		Cytoplasmic	others	N	0.334	N
P02649	APOE	317	O		Secretion	classical secretion	Y	0.88	N
P15289	ARSA	507	O		Secretion	classical secretion	Y	0.831	N
O43776	NARS	548	O	O	Cytoplasmic	others	N	0.406	N
P17174	GOT1	413	O		Cytoplasmic	others	N	0.439	N
P00505	GOT2	430	O		Secretion	nonclassical secretion	N	0.505	N
Q9ULA0	DNPEP ASPEP DAP	475		O	Cytoplasmic	others	N	0.347	N
P06576	ATP5B ATPMB ATPSB	529	O		Secretion	nonclassical secretion	N	0.591	N
P61221	RNASEL1 RNASELI RNS4I (	599		O	Cytoplasmic	others	N	0.296	N
P17858	PFKL	780	O	O	Cytoplasmic	others	N	0.417	N
O75882	ATRN KIAA0548 MGCA	1429	O		Secretion	membrane integral protein	N	0.298	Y
P15291	B4GALT1 GGTB2	398	O		Secretion	nonclassical secretion	N	0.732	Y
O43505	B4GAT1 B3GNT1 B3GNT6	415		O	Secretion	nonclassical secretion	N	0.914	Y
P61769	32M CDABP0092 HDCMA22I	119	O		Secretion	classical secretion	Y	0.907	N
P08236	GUSB	651	O	O	Secretion	classical secretion	Y	0.582	Y
P06865	HEXA	529	O		Secretion	classical secretion	Y	0.701	N

P07686	HEXB HCC7	556	O	O	Secretion	nonclassical secretion	N	0.712	Y
P21810	BGN SLRR1A	368		O	Secretion	classical secretion	Y	0.714	N
P54687	BCAT1 BCT1 ECA39	386	O	O	Secretion	nonclassical secretion	N	0.507	N
Q96GW7	BEHAB CSPG7 UNQ2525/PF	911		O	Secretion	classical secretion	Y	0.522	N
Q9HCU4	2 CDHF10 EGFL2 KIAA0279	2923	O	O	Secretion	classical secretion	Y	0.327	Y
P19022	CDH2 CDHN NCAD	906	O	O	Secretion	classical secretion	Y	0.203	Y
Q9Y2V2	CARHSP1	147		O	Secretion	nonclassical secretion	N	0.83	N
Q9HB71	CYBP S100A6BP SIP PNAS-	228	O	O	Secretion	nonclassical secretion	N	0.69	N
Q05682	CALD1 CAD CDM	793		O	Cytoplasmic	others	N	0.443	N
P62158	ALM2 CAM2 CAMB; CALM3	149	O	O	Secretion	nonclassical secretion	N	0.676	N
Q5T5Y3	CAMSAP1	1602		O	Cytoplasmic	others	N	0.135	N
P20810	CAST	708	O	O	Cytoplasmic	others	N	0.092	N
Q15417	CNN3	329	O		Secretion	nonclassical secretion	N	0.643	N
P27797	CALR CRTC	417	O	O	Secretion	classical secretion	Y	0.366	N
O94985	CLSTN1 CS1 KIAA0911	981	O	O	Secretion	classical secretion	Y	0.436	Y
O43852	CALU	315	O		Secretion	classical secretion	Y	0.753	N
Q14444	I1 GPIAP1 GPIP137 M11S1 I	709	O		Cytoplasmic	others	N	0.099	N
O43570	CA12	354		O	Secretion	classical secretion	Y	0.323	Y
P00918	CA2	260	O		Multiple location	others	N	0.411	N
P16152	CBR1 CBR CRN SDR21C1	277	O	O	Secretion	nonclassical secretion	N	0.633	N
O75828	CBR3	277		O	Secretion	nonclassical secretion	N	0.674	N
P16870	CPE	476	O		Secretion	classical secretion	Y	0.464	N
P68400	CSNK2A1 CK2A1	391	O		Secretion	nonclassical secretion	N	0.735	N
P19784	CSNK2A2 CK2A2	350		O	Secretion	others	N	0.295	N
P07858	CTSB CPSB	339	O	O	Secretion	classical secretion	Y	0.77	N
Q9UBR2	CTSZ	303	O		Secretion	classical secretion	Y	0.861	Y
Q6YHK3	CD109 CPAMD7	1445	O		Secretion	classical secretion	Y	0.6	N
Q5ZPR3	B7H3 PSEC0249 UNQ309/F	534	O		Secretion	classical secretion	Y	0.65	Y
P60033	CD81 TAPA1 TSPAN28	236		O	Secretion	nonclassical secretion	N	0.501	Y
Q8NFZ8	CADM4 IGSF4C NECL4 TSLL	388		O	Secretion	classical secretion	Y	0.66	Y
P36222	CHI3L1	383	O	O	Secretion	classical secretion	Y	0.645	N
O00299	CLIC1 G6 NCC27	241	O	O	Nucleus	others	N	0.395	N
Q9Y696	CLIC4	253	O		Secretion	nonclassical secretion	N	0.612	N
Q13185	CBX3	183	O	O	Secretion	nonclassical secretion	N	0.836	N
Q6ZTR5	7 CHDC2 CXorf22 CXorf30 C	3102	O		Unknown	others	N	0.362	N
Q00610	LTC CLH17 CLTCL2 KIAA00	1675	O	O	Secretion	others	N	0.437	N
Q16630	CPSF6 CFIM68	551	O		Nucleus	others	N	0.221	N
P10909	CLU APOJ CLI KUB1 AAG4	449	O	O	Secretion	classical secretion	Y	0.826	N
P23528	CFL1 CFL	166	O		Secretion	nonclassical secretion	N	0.628	N
Q4VC31	CCDC58	144	O		Secretion	nonclassical secretion	N	0.893	N
Q14011	CIRBP A18HNRNP CIRP	172	O		Secretion	nonclassical secretion	N	0.77	N
P02452	COL1A1	1464	O		Secretion	classical secretion	Y	0.206	N
P12109	COL6A1	1028	O	O	Secretion	classical secretion	Y	0.234	N
Q02388	COL7A1	2944	O		Secretion	classical secretion	Y	0.065	N
P12110	COL6A2	1019	O		Secretion	classical secretion	Y	0.214	N
P25940	COL5A3	1745	O		Secretion	classical secretion	Y	0.047	N
P00736	C1R	705	O	O	Secretion	classical secretion	Y	0.697	N
P0C0L4	C4A CO4 CPAMD2	1744	O	O	Secretion	classical secretion	Y	0.399	N
Q07021	.QBP GC1QBP HABP1 SF2F	282	O	O	Secretion	nonclassical secretion	N	0.622	N
P08603	CFH HF HF1 HF2	1231	O		Secretion	classical secretion	Y	0.399	N
P12277	CKB CKBB	381	O	O	Cytoplasmic	others	N	0.258	N
P46109	CRKL	303	O		Secretion	nonclassical secretion	N	0.624	N
P17812	CTPS1 CTPS	591	O		Cytoplasmic	others	N	0.388	N
Q9UBG0	.EC13E ENDO180 KIAA0709	1479	O	O	Secretion	classical secretion	Y	0.491	Y
Q13616	CUL1	776	O		Nucleus	others	N	0.278	N
P11802	CDK4	303		O	Multiple location	others	N	0.265	N
P04080	CSTB CST6 STFB	98	O	O	Multiple location	others	N	0.333	N
P01034	CST3	146	O	O	Secretion	classical secretion	Y	0.937	Y
P21291	CSRP1 CSRP CYRP	193		O	Nucleus	others	N	0.246	N
P52943	CRIP2 CRP2	208	O	O	Secretion	nonclassical secretion	N	0.872	N
P99999	CYCS CYC	105	O		Organellar	others	N	0.465	N
Q14204	C1 DNCH1 DNCL DNECL DY	4646	O	O	Cytoplasmic	others	N	0.267	N
P28838	LAP3 LAPEP PEPS	519	O	O	Cytoplasmic	others	N	0.463	N
O00154	ACOT7 BACH	380		O	Secretion	nonclassical secretion	N	0.666	N
Q96KP4	DP2 CN2 CPGL HEL-S-13 PI	475	O	O	Cytoplasmic	others	N	0.443	N
O43175	PHGDH PGDH3	533	O		Multiple location	others	N	0.481	N
Q9H773	DCTPP1 XTP3TPA CDA03	170		O	Secretion	nonclassical secretion	N	0.77	N
P09417	QDPR DHPR SDR33C1	244	O	O	Organellar	others	N	0.442	N
Q16555	DPYSL2 CRMP2 ULIP2	572	O	O	Multiple location	others	N	0.411	N
Q14195	YSL3 CRMP4 DRP3 ULIP UL	570	O	O	Cytoplasmic	others	N	0.444	N
O14531	DPYSL4 CRMP3 ULIP4	572		O	Cytoplasmic	others	N	0.442	N
Q12882	DPYD	1025	O		Cytoplasmic	others	N	0.456	N
P53634	CTSC CPPI	463	O		Secretion	classical secretion	Y	0.767	N
Q9NY33	DPP3	737	O	O	Cytoplasmic	others	N	0.408	N
P27487	DPP4 ADCP2 CD26	766		O	Secretion	nonclassical secretion	N	0.719	Y
Q13443	19 KIAA0021 MCMP MDC9 M	819		O	Secretion	classical secretion	Y	0.112	Y
Q16531	DDB1 XAP1	1140	O	O	Secretion	nonclassical secretion	N	0.554	N
O00273	DFFA DFF1 DFF45 H13	331	O	O	Cytoplasmic	others	N	0.449	N
P49736	2 BM28 CCNL1 CDCL1 KIAA	904	O		Nucleus	others	N	0.308	N
P27695	. APE APE1 APEX APX HAP1	318	O	O	Secretion	nonclassical secretion	N	0.655	N
Q9UBS4	ERJ3 HDJ9 PSEC0121 UNQ	358		O	Secretion	classical secretion	Y	0.707	Y
Q13217	DNAJC3 P58IPK PRKRI	504	O		Secretion	classical secretion	Y	0.38	N
Q16643	DBN1 D0S117E	649		O	Cytoplasmic	others	N	0.091	N
Q14118	DAG1	895	O	O	Secretion	classical secretion	Y	0.11	Y
Q7Z6Z7	KIAA0312 KIAA1578 UREB1	4374		O	Multiple location	others	N	0.38	N
Q15075	EEA1 ZFYVE2	1411		O	Cytoplasmic	others	N	0.11	N
Q12805	EFEMP1 FBLN3 FBNL	493		O	Secretion	classical secretion	Y	0.719	N
O43854	EDIL3 DEL1	480	O		Secretion	classical secretion	Y	0.821	N
P68104	EEF1A1 EEF1A EF1A LENG7	462	O	O	Multiple location	others	N	0.155	N
P24534	EEF1B2 EEF1B EF1B	225		O	Secretion	nonclassical secretion	N	0.562	N
P29692	EEF1D EF1D	281	O	O	Secretion	nonclassical secretion	N	0.529	N
P13639	EEF2 EF2	858	O	O	Multiple location	others	N	0.38	N
Q9NZ08	ILS ARTS1 KIAA0525 UNQ5	941	O		Secretion	classical secretion	Y	0.609	N
P14625	HSP90B1 GRP94 TRA1	803		O	Secretion	classical secretion	Y	0.495	N
Q9UNN8	PROCR EPCR	238	O		Secretion	classical secretion	Y	0.916	Y
Q9UHY7	ENOPH1 MASA MSTP145	261	O	O	Secretion	nonclassical secretion	N	0.699	N
P30084	ECHS1	290	O		Secretion	nonclassical secretion	N	0.855	N
P54756	PHA5 BSK EHK1 HEK7 TYR	1037	O		Membrane	others	N	0.401	N
P61916	NPC2 HE1	151	O	O	Secretion	classical secretion	Y	0.931	N
P30042	C21orf33 HES1 KNPI	268	O	O	Secretion	classical secretion	Y	0.877	N
P60842	EIF4A1 DDX2A EIF4A	406		O	Secretion	nonclassical secretion	N	0.631	N
O60739	EIF1B	113		O	Secretion	nonclassical secretion	N	0.712	N
O15371	EIF3D EIF3S7	548	O		Cytoplasmic	others	N	0.203	N
P23588	EIF4B	611	O	O	Secretion	nonclassical secretion	N	0.573	N
P63241	EIF5A	154	O		Multiple location	others	N	0.319	N

Q9GZV4	EIF5A2	153		O	Multiple location	others	N	0.254	N
P43003	LC1A3 EAAT1 GLAST GLAS	542		O	Membrane	membrane integral protein	N	0.226	Y
P15311	EZR VIL2	586	O		Secretion	nonclassical secretion	N	0.563	N
P47755	CAPZA2	286	O	O	Cytoplasmic	others	N	0.454	N
P47756	CAPZB	277	O	O	Secretion	nonclassical secretion	N	0.543	N
P14324	FDPS FPS KIAA1293	419	O	O	Secretion	nonclassical secretion	N	0.639	N
Q16658	FSCN1 FAN1 HSN SNL	493	O	O	Cytoplasmic	others	N	0.385	N
P49327	FASN FAS	2511	O	O	Cytoplasmic	others	N	0.408	N
O15540	FABP7 BLBP FABPB MRG	132	O	O	Cytoplasmic	others	N	0.422	N
Q01469	FABP5	135		O	Cytoplasmic	others	N	0.363	N
Q9BZK7	TBL1XR1 IRA1 TBLR1	514	O		Secretion	nonclassical secretion	N	0.553	N
Q96AC1	ERMT2 KIND2 MIG2 PLEKH(	680		O	Multiple location	others	N	0.267	N
P02792	FTL	175	O		Cytoplasmic	others	N	0.383	N
P35555	FBN1 FBN	2871	O		Secretion	classical secretion	Y	0.391	N
P02751	FN1 FN	2386	O	O	Secretion	classical secretion	Y	0.369	N
Q53RD9	FBLN7 TM14	439	O		Secretion	classical secretion	Y	0.762	N
P21333	FLNA FLN FLN1	2647	O	O	Cytoplasmic	others	N	0.446	N
O75369	FLNB FLN1L FLN3 TABP TAI	2602	O		Cytoplasmic	others	N	0.359	N
Q12841	FSTL1 FRP	308	O		Secretion	classical secretion	Y	0.533	N
Q13642	FHL1 SLIM1	323		O	Secretion	nonclassical secretion	N	0.707	N
P04075	ALDOA ALDA	364	O	O	Cytoplasmic	others	N	0.356	N
P09972	ALDOC ALDC	364		O	Secretion	others	N	0.323	N
P07954	FH	510	O	O	Secretion	nonclassical secretion	N	0.551	N
P16930	FAH	419	O		Secretion	nonclassical secretion	N	0.582	N
P09382	LGALS1	135	O		Secretion	others	N	0.345	N
P17931	LGALS3 MAC2	250	O		Secretion	nonclassical secretion	N	0.77	N
Q08380	LGALS3BP M2BP	585	O	O	Secretion	classical secretion	Y	0.738	N
P09104	ENO2	434	O	O	Secretion	nonclassical secretion	N	0.599	N
Q92820	GGH	318	O		Secretion	classical secretion	Y	0.628	N
O75223	GGCT C7orf24 CRF21	188	O		Secretion	nonclassical secretion	N	0.503	N
P17900	GM2A	193	O		Secretion	classical secretion	Y	0.945	N
Q9H488	POFUT1 FUT12 KIAA0180	388	O		Secretion	classical secretion	Y	0.686	N
Q13630	TSTA3 SDR4E1	321	O	O	Multiple location	others	N	0.473	N
P06396	GSN	782	O	O	Secretion	classical secretion	Y	0.553	N
P07093	SERPINE2 PI7 PN1	398		O	Secretion	classical secretion	Y	0.692	N
P14136	GFAP	432		O	Secretion	nonclassical secretion	N	0.693	N
P46926	GNPDA1 GNPI HLN KIAA006	289		O	Cytoplasmic	others	N	0.333	N
Q8TDQ7	GNPDA2 GNP2	276	O		Secretion	nonclassical secretion	N	0.508	N
P11413	G6PD	515	O	O	Secretion	others	N	0.449	N
P06744	GPI	558	O	O	Secretion	others	N	0.453	N
P00367	GLUD1 GLUD	558	O	O	Organellar	others	N	0.363	N
P15104	GLUL GLNS	373	O	O	Cytoplasmic	others	N	0.394	N
Q16769	QPCT	361	O		Secretion	classical secretion	Y	0.788	N
P00390	GSR GLUR GRD1	522	O	O	Secretion	nonclassical secretion	N	0.54	N
P21266	GSTM3 GST5	225	O		Cytoplasmic	others	N	0.329	N
P78417	GSTO1 GSTTLP28	241		O	Cytoplasmic	others	N	0.435	N
P09211	GSTP1 FAEES3 GST3	210	O	O	Secretion	nonclassical secretion	N	0.545	N
P48637	GSS	474	O		Nucleus	others	N	0.484	N
P04406	H GAPD CDABP0047 OK/SW	335	O	O	Multiple location	others	N	0.467	N
P50440	GATM AGAT	423	O		Secretion	nonclassical secretion	N	0.699	N
P23434	GCSH	173	O		Secretion	classical secretion	Y	0.85	N
P41250	GARS	739	O	O	Secretion	classical secretion	Y	0.483	N
P11216	PYGB	843		O	Cytoplasmic	others	N	0.422	N
P06737	PYGL	847		O	Multiple location	others	N	0.381	N
Q9HC38	LOD4 C17orf25 CGI-150 My0	313	O	O	Secretion	nonclassical secretion	N	0.721	N
P35052	GPC1	558	O	O	Secretion	classical secretion	Y	0.33	N
O75487	GPC4 UNQ474/PRO937	556	O		Secretion	classical secretion	Y	0.687	N
Q8NBJ4	.55 GOLPH2 PSEC0242 UN(	401		O	Secretion	nonclassical secretion	N	0.547	Y
Q8NCC3	2G15 LYPLA3 UNQ341/PRC	412	O		Secretion	classical secretion	Y	0.684	N
P09341	L GRO GRO1 GROA MGSA	107	O		Secretion	classical secretion	Y	0.624	N
P30047	GCHFR GFRP	84	O		Secretion	nonclassical secretion	N	0.93	N
P62826	RAN ARA24 OK/SW-cl.81	216	O		Secretion	nonclassical secretion	N	0.582	N
P08754	GNAI3	354		O	Secretion	nonclassical secretion	N	0.527	N
P0DMV9	HSPA1B	641		O	Cytoplasmic	others	N	0.28	N
P34931	HSPA1L	641	O		Multiple location	others	N	0.37	N
P34932	HSPA4 APG2	840	O	O	Cytoplasmic	others	N	0.226	N
P17066	HSPA6 HSP70B'	643		O	Secretion	others	N	0.269	N
P11142	SPA8 HSC70 HSP73 HSPA1	646	O	O	Multiple location	others	N	0.229	N
O75506	HSBP1 HSF1BP	76	O		Nucleus	others	N	0.421	N
P04792	HSPB1 HSP27 HSP28	205		O	Secretion	nonclassical secretion	N	0.74	N
P07900	P90AA1 HSP90A HSPC1 HSI	732	O	O	Multiple location	others	N	0.173	N
P08238	P90AB1 HSP90B HSPC2 HSI	724	O	O	Cytoplasmic	others	N	0.204	N
Q9UK76	HN1 ARM2	154		O	Nucleus	others	N	0.267	N
Q14CZ8	HEPACAM	416		O	Secretion	classical secretion	Y	0.211	Y
P51858	HDGF HMG1L2	240	O	O	Multiple location	others	N	0.477	N
Q99729	HNRNPAB ABBP1 HNRPAB	332	O	O	Multiple location	others	N	0.147	N
Q32P51	HNRNPA1L2 HNRNPA1L	320	O		Multiple location	others	N	0.135	N
P51991	HNRNPA3 HNRPA3	378		O	Nucleus	others	N	0.112	N
Q14103	HNRNPD AUF1 HNRPD	355	O		Multiple location	others	N	0.107	N
P52597	HNRNPF HNRPF	415	O	O	Nucleus	others	N	0.475	N
P61978	HNRNPK HNRPK	463		O	Multiple location	others	N	0.177	N
O60506	SYNCRIP HNRPQ NSAP1	623		O	Multiple location	others	N	0.081	N
O43390	HNRNPR HNRPR	633	O		Multiple location	others	N	0.077	N
P22626	HNRNPA2B1 HNRPA2B1	353	O	O	Secretion	others	N	0.081	N
P09429	HMGB1 HMG1	215	O		Secretion	others	N	0.068	N
P17096	HMGA1 HMGIY	107	O	O	Nucleus	others	N	0.407	N
P49773	HINT1 HINT PKCI1 PRKCNH	126	O		Secretion	nonclassical secretion	N	0.553	N
Q96KK5	HIST1H2AH HIST1H2AI	128	O	O	Secretion	nonclassical secretion	N	0.563	N
Q8IUE6	HIST2H2AB	130	O		Secretion	nonclassical secretion	N	0.517	N
O60814	HIST1H2BK H2BFT HIRIP1	126	O		Nucleus	others	N	0.305	N
P62805	H4/H H4FH; HIST1H4I H4/M	103	O		Nucleus	others	N	0.408	N
Q9NWY4	HPF1 C4orf27	346	O		Secretion	nonclassical secretion	N	0.733	N
P01892	HLA-A HLAA	365		O	Secretion	classical secretion	Y	0.214	Y
P10314	HLA-A HLAA	365	O		Secretion	classical secretion	Y	0.186	Y
Q9NSC5	HOMER3	361	O		Multiple location	others	N	0.096	N
Q16836	HADH HAD HADHSC SCHAD	314	O		Organellar	others	N	0.492	N
Q16775	HAGH GLO2 HAGH1	308		O	Secretion	nonclassical secretion	N	0.633	N
Q01581	HMGCS1 HMGCS	520		O	Secretion	nonclassical secretion	N	0.56	N
Q9Y4L1	HYOU1 GRP170 ORP150	999	O		Secretion	classical secretion	Y	0.32	Y
P22304	IDS SIDS	550	O		Secretion	classical secretion	Y	0.799	N
Q14974	KPNB1 NTF97	876	O	O	Secretion	nonclassical secretion	N	0.588	N
O95373	IPO7 RANBP7	1038	O	O	Secretion	nonclassical secretion	N	0.532	N
Q13308	PTK7 CCK4	1070	O	O	Secretion	classical secretion	Y	0.391	Y
Q15181	PPA1 IOPPP PP	289		O	Cytoplasmic	others	N	0.433	N
Q9BY32	PA C20orf37 My049 OK/SW-(	194		O	Secretion	nonclassical secretion	N	0.623	N

P12268	IMPDH2 IMPD2	514		O	Multiple location	others	N	0.354	N
P18065	IGFBP2 BP2 IBP2	325	O	O	Secretion	classical secretion	Y	0.886	N
P17936	IGFBP3 IBP3	291	O		Secretion	classical secretion	Y	0.815	N
Q16270	IGFBP7 MAC25 PSF	282	O	O	Secretion	classical secretion	Y	0.536	N
P05556	ITGB1 FNRB MDF2 MSK12	798	O		Secretion	classical secretion	Y	0.474	Y
Q12905	ILF2 NF45 PRO3063	390	O	O	Multiple location	others	N	0.272	N
P10145	CXCL8 IL8	99	O		Secretion	classical secretion	Y	0.618	Y
O75874	IDH1 PICD	414	O	O	Secretion	nonclassical secretion	N	0.547	N
P13645	KRT10 KPP	584	O		Cytoplasmic	others	N	0.123	N
P13646	KRT13	458	O		Cytoplasmic	others	N	0.345	N
P08779	KRT16 KRT16A	473	O		Cytoplasmic	others	N	0.25	N
P35527	KRT9	623	O	O	Secretion	others	N	0.089	N
P04264	KRT1 KRTA	644	O	O	Membrane	others	N	0.099	N
P19013	KRT4 CYK4	534	O		Membrane	membrane integral protein	N	0.18	Y
Q07666	KHDRBS1 SAM68	443	O		Multiple location	others	N	0.387	N
Q86UP2	KTN1 CG1 KIAA0004	1357		O	Organellar	membrane integral protein	N	0.101	Y
Q04760	GLO1	184	O	O	Multiple location	others	N	0.397	N
P20700	LMNB1 LMN2 LMNB	586	O		Nucleus	others	N	0.412	N
Q16363	LAMA4	1823	O		Secretion	classical secretion	Y	0.399	N
P07942	LAMB1	1786	O		Secretion	classical secretion	Y	0.352	N
P55268	LAMB2 LAMS	1798	O		Secretion	classical secretion	Y	0.261	N
P11047	LAMC1 LAMB2	1609	O		Secretion	classical secretion	Y	0.284	N
Q8N2S1	LTBP4	1624	O	O	Secretion	classical secretion	Y	0.28	N
P42704	LRPPRC LRP130	1394	O		Multiple location	others	N	0.35	N
Q96AG4	LRRC59 PRO1855	307	O		Secretion	nonclassical secretion	N	0.655	Y
P09960	LTA4H LTA4	611	O	O	Cytoplasmic	others	N	0.485	N
P00338	LDHA PIG19	332	O	O	Secretion	nonclassical secretion	N	0.549	N
P07195	LDHB	334	O	O	Secretion	nonclassical secretion	N	0.569	N
P01130	LDLR	860	O		Secretion	classical secretion	Y	0.475	Y
P51884	LUM LDC SLRR2D	338	O		Secretion	classical secretion	Y	0.543	N
P05455	SSB	408	O	O	Nucleus	others	N	0.266	N
P38571	LIPA	399	O		Secretion	classical secretion	Y	0.768	N
P09603	CSF1	554	O	O	Secretion	classical secretion	Y	0.488	Y
P40121	CAPG AFCP MCP	348	O		Secretion	others	N	0.439	N
P40925	MDH1 MDHA	334	O	O	Cytoplasmic	others	N	0.455	N
P40926	MDH2	338	O	O	Secretion	nonclassical secretion	N	0.644	N
P01033	TIMP1 CLGI TIMP	207	O	O	Secretion	classical secretion	Y	0.765	N
P16035	TIMP2	220		O	Secretion	classical secretion	Y	0.854	N
Q9NZL9	MSTP045 Nbla02999 UNQ24	334		O	Secretion	nonclassical secretion	N	0.519	N
P56192	MARS	900		O	Secretion	nonclassical secretion	N	0.541	N
P46821	MAP1B	2468	O	O	Cytoplasmic	others	N	0.088	N
P11137	MAP2	1827	O		Cytoplasmic	others	N	0.188	N
P27816	MAP4	1152	O	O	Cytoplasmic	others	N	0.251	N
Q15691	MAPRE1	268		O	Multiple location	others	N	0.356	N
P21741	MDK MK1 NEGF2	143	O	O	Secretion	classical secretion	Y	0.259	Y
P28482	MAPK1 ERK2 PRKM1 PRKM	360	O	O	Multiple location	others	N	0.352	N
Q12851	MAP4K2 GCK RAB8IP	820		O	Multiple location	others	N	0.343	N
P26038	MSN	577	O	O	Secretion	nonclassical secretion	N	0.53	N
Q969H8	MYDGF C19orf10 IL25	173	O		Secretion	classical secretion	Y	0.906	N
P60660	MYL6	151	O		Multiple location	others	N	0.427	N
P19105	MYL12A MLCB MRLC3 RLC	171	O		Secretion	nonclassical secretion	N	0.607	N
P35580	MYH10	1976	O		Cytoplasmic	others	N	0.069	N
P58546	MTPN	118	O	O	Multiple location	others	N	0.325	N
P29966	MARCKS MACS PRKCSL	332	O	O	Multiple location	others	N	0.207	N
O94760	DDAH1 DDAH	285		O	Multiple location	others	N	0.47	N
Q9UJ70	NAGK	344	O		Secretion	nonclassical secretion	N	0.664	N
Q8NCW5	IAXE AIBP APOA1BP YJEFN	288		O	Secretion	classical secretion	Y	0.819	N
P48163	ME1	572	O		Cytoplasmic	others	N	0.378	N
Q13765	NACA HSD48	215	O		Multiple location	others	N	0.301	N
Q9NQS3	NECTIN3 PRR3 PVRL3	549	O		Membrane	membrane integral protein	N	0.464	Y
Q13564	NAE1 APPBP1 HPP1	534		O	Secretion	nonclassical secretion	N	0.56	N
P48681	NES Nbla00170	1621	O	O	Cytoplasmic	others	N	0.256	N
O14594	NCAN CSPG3 NEUR	1321		O	Secretion	classical secretion	Y	0.21	N
P29120	PCSK1 NEC1	753	O		Secretion	classical secretion	Y	0.366	N
Q9BYT8	NLN AGTBP KIAA1226	704	O		Cytoplasmic	others	N	0.45	N
P17677	GAP43	238	O	O	Membrane	others	N	0.412	N
Q92823	NRCAM KIAA0343	1304	O	O	Secretion	classical secretion	Y	0.332	Y
Q15818	NPTX1	432	O		Secretion	classical secretion	Y	0.6	N
Q14697	GANAB G2AN KIAA0088	944	O	O	Secretion	classical secretion	Y	0.64	Y
P43490	NAMPT PBEF PBEF1	491	O	O	Secretion	others	N	0.302	N
P14543	NID1 NID	1247	O		Secretion	classical secretion	Y	0.644	N
Q14112	NID2	1375	O		Secretion	classical secretion	Y	0.558	N
Q9UNZ2	NSFL1C UBXN2C	370		O	Multiple location	others	N	0.359	N
P49321	NASP	788	O	O	Multiple location	others	N	0.089	N
Q9Y266	NUDC	331		O	Multiple location	others	N	0.448	N
P61970	NUTF2 NTF2	127	O	O	Secretion	nonclassical secretion	N	0.625	N
P67809	YBX1 NSEP1 YB1	324		O	Secretion	nonclassical secretion	N	0.733	N
Q02818	NUCB1 NUC	461	O	O	Secretion	classical secretion	Y	0.305	N
P19338	NCL	710	O		Nucleus	others	N	0.386	N
Q6NW34	NEPRO C3orf17	567		O	Secretion	nonclassical secretion	N	0.866	N
P06748	NPM1 NPM	294	O	O	Secretion	nonclassical secretion	N	0.811	N
P22392	NME2 NM23B	152	O		Multiple location	others	N	0.33	N
P55209	NAP1L1 NRP	391	O		Multiple location	others	N	0.042	N
Q9NQR4	NIT2 CUA002	276	O		Secretion	nonclassical secretion	N	0.663	N
P10451	SPP1 BNSP OPN PSEC0156	314	O		Secretion	classical secretion	Y	0.656	N
P50897	PPT1 CLN1 PPT	306	O		Secretion	classical secretion	Y	0.84	N
Q96RD6	PANX2	677	O		Secretion	nonclassical secretion	N	0.544	Y
P62937	PPIA CYPA	165	O	O	Secretion	others	N	0.339	N
P23284	PPIB CYPB	216		O	Secretion	nonclassical secretion	N	0.853	Y
P30405	PPIF CYP3	207	O		Secretion	nonclassical secretion	N	0.683	N
P62942	FKBP1A FKBP1 FKBP12	108	O	O	Cytoplasmic	others	N	0.368	N
Q02790	FKBP4 FKBP52	459	O	O	Multiple location	others	N	0.287	N
O60664	PLIN3 M6PRBP1 TIP47	434	O		Secretion	nonclassical secretion	N	0.559	N
Q06830	PRDX1 PAGA PAGB TDPX2	199	O	O	Secretion	nonclassical secretion	N	0.528	N
P32119	PRDX2 NKEFB TDPX1	198	O	O	Secretion	nonclassical secretion	N	0.522	N
P30041	PRDX6 AOP2 KIAA0106	224	O	O	Secretion	others	N	0.378	N
P30086	PEBP1 PBP PEBP	187	O	O	Secretion	nonclassical secretion	N	0.672	N
P42336	PIK3CA	1068	O		Cytoplasmic	others	N	0.414	N
P36871	PGM1	562	O	O	Cytoplasmic	others	N	0.417	N
P00558	K1 PGKA MIG10 OK/SW-cl.	417	O	O	Cytoplasmic	others	N	0.389	N
P07205	PGK2 PGKB	417	O		Cytoplasmic	others	N	0.361	N
P18669	PGAM1 PGAMA CDABP0006	254	O	O	Nucleus	others	N	0.407	N
Q9Y617	PSAT1 PSA	370	O	O	Cytoplasmic	others	N	0.384	N
P36955	SERPINF1 PEDF PIG35	418		O	Secretion	classical secretion	Y	0.825	N
Q9GZP4	D1 C1orf128 AD039 HT014 F	211		O	Secretion	nonclassical secretion	N	0.68	N

Q9BTY2	CA2 PSEC0151 UNQ227/PRC	467		O	Secretion	classical secretion	Y	0.779	N
P13797	PLS3	630	O		Cytoplasmic	others	N	0.429	N
P43034	AH1B1 LIS1 MDCR MDS PAF	410	O	O	Multiple location	others	N	0.356	N
P68402	PAFAH1B2 PAFAHB	229	O		Cytoplasmic	others	N	0.47	N
Q15365	PCBP1	356	O	O	Secretion	nonclassical secretion	N	0.549	N
P11940	ABPC1 PAB1 PABP1 PABPC	636	O		Multiple location	others	N	0.283	N
Q13310	PABPC4 APP1 PABP4	644		O	Cytoplasmic	others	N	0.151	N
P26599	PTBP1 PTB	531	O		Nucleus	others	N	0.415	N
P0CG48	UBC	685	O		Multiple location	others	N	0.337	N
P0CG39	POTEJ	1038	O		Secretion	others	N	0.355	N
Q9UHV9	PFDN2 PFD2 HSPC231	154		O	Secretion	nonclassical secretion	N	0.583	N
Q99471	PFDN5 MM1 PFD5	154	O	O	Nucleus	others	N	0.296	N
P02545	LMNA LMN1	664	O	O	Nucleus	others	N	0.077	N
Q15113	PCOLCE PCPE1	449	O		Secretion	classical secretion	Y	0.699	N
Q02809	PLOD1 LLH PLOD	727	O	O	Secretion	classical secretion	Y	0.573	N
O00469	PLOD2	737	O		Secretion	classical secretion	Y	0.634	N
O60568	PLOD3	738	O	O	Secretion	classical secretion	Y	0.561	N
P07737	PFN1	140	O	O	Cytoplasmic	others	N	0.469	N
P35080	PFN2	140	O	O	Secretion	nonclassical secretion	N	0.552	N
P12004	PCNA	261		O	Secretion	nonclassical secretion	N	0.566	N
Q9UQ80	PA2G4 EBP1	394	O	O	Multiple location	others	N	0.201	N
Q32P28	H1 GROS1 LEPRE1 PSEC01	736	O		Secretion	classical secretion	Y	0.805	N
P48147	PREP PEP	710	O		Secretion	nonclassical secretion	N	0.543	N
P07602	PSAP GLBA SAP1	524	O		Secretion	classical secretion	Y	0.785	N
Q15185	PTGES3 P23 TEBP	160		O	Secretion	nonclassical secretion	N	0.729	N
Q14914	PTGR1 LTB4DH	329	O		Cytoplasmic	others	N	0.367	N
P41222	PTGDS PDS	190	O	O	Secretion	classical secretion	Y	0.772	N
Q9UL46	PSME2	239	O		Nucleus	others	N	0.363	N
Q9BT73	PSMG3 C7orf48 PAC3	122		O	Secretion	nonclassical secretion	N	0.593	N
P25787	PSMA2 HC3 PSC3	234	O	O	Multiple location	others	N	0.33	N
P25788	PSMA3 HC8 PSC8	255	O		Multiple location	others	N	0.256	N
P25789	PSMA4 HC9 PSC9	261	O	O	Multiple location	others	N	0.209	N
P28066	PSMA5	241	O	O	Multiple location	others	N	0.485	N
O14818	PSMA7 HSPC	248	O	O	Multiple location	others	N	0.248	N
P20618	PSMB1 PSC5	241	O	O	Multiple location	others	N	0.473	N
P40306	PSMB10 LMP10 MECL1	273	O	O	Secretion	nonclassical secretion	N	0.515	N
P49721	PSMB2	201	O	O	Multiple location	others	N	0.398	N
P49720	PSMB3	205	O	O	Multiple location	others	N	0.401	N
P28070	PSMB4 PROS26	264	O		Secretion	nonclassical secretion	N	0.565	N
P28072	PSMB6 LMPY Y	239	O		Secretion	nonclassical secretion	N	0.563	N
P28062	MB8 LMP7 PSMB5i RING10	276	O		Secretion	nonclassical secretion	N	0.517	N
P28065	PSMB9 LMP2 PSMB6i RING1	219	O	O	Secretion	nonclassical secretion	N	0.557	N
Q9UKY7	CDV3 H41	258	O		Cytoplasmic	others	N	0.441	N
Q99497	PARK7	189	O	O	Membrane	others	N	0.493	N
P30101	PDIA3 ERP57 ERP60 GRP58	505	O		Secretion	classical secretion	Y	0.707	N
P13667	PDIA4 ERP70 ERP72	645	O	O	Secretion	classical secretion	Y	0.543	Y
Q15084	PDIA6 ERP5 P5 TXNDC7	440	O		Secretion	classical secretion	Y	0.711	N
P07237	HB ERBA2L PDI PDIA1 PO4	508	O	O	Secretion	classical secretion	Y	0.679	N
P49257	LMAN1 ERGIC53 F5F8D	510		O	Secretion	classical secretion	Y	0.589	Y
Q92520	FAM3C ILEI GS3786	227	O	O	Secretion	nonclassical secretion	N	0.821	Y
P49354	FNTA	379	O		Cytoplasmic	others	N	0.401	N
P78504	JAG1 JAGL1	1218	O	O	Secretion	classical secretion	Y	0.306	Y
P48745	NOV CCN3 IGFBP9 NOVH	357	O		Secretion	classical secretion	Y	0.888	N
Q96C90	PPP1R14B PLCB3N PNG	147	O		Secretion	nonclassical secretion	N	0.763	N
Q15435	PPP1R7 SDS22	360	O		Secretion	nonclassical secretion	N	0.777	N
Q9P258	RCC2 KIAA1470 TD60	522	O	O	Multiple location	others	N	0.343	N
P60903	100A10 ANX2LG CAL1L CLP	97	O		Membrane	membrane integral protein	N	0.121	Y
Q15436	SEC23A	765	O		Secretion	nonclassical secretion	N	0.612	N
O94855	SEC24D KIAA0755	1032	O		Secretion	nonclassical secretion	N	0.545	N
Q9H993	ARMT1 C6orf211	441		O	Secretion	nonclassical secretion	N	0.557	N
Q96JQ0	DH19 CDH25 FIB1 KIAA177	3298		O	Secretion	classical secretion	Y	0.448	Y
P61457	PCBD1 DCOH PCBD	104		O	Multiple location	others	N	0.461	N
P00491	PNP NP	289	O	O	Secretion	nonclassical secretion	N	0.509	N
A6NMY6	XA2P2 ANX2L2 ANX2P2 LPC	339	O	O	Secretion	nonclassical secretion	N	0.732	N
B2RPK0	HMGB1P1 HMG1L1 HMGB1L	211		O	Nucleus	others	N	0.048	N
O60361	NME2P1	137		O	Secretion	nonclassical secretion	N	0.578	N
O00764	21orf124 C21orf97 PKH PNK	312	O		Secretion	nonclassical secretion	N	0.511	N
Q96GD0	PDXP CIN PLP PLPP	296		O	Cytoplasmic	others	N	0.451	N
Q9NVS9	PNPO	261	O		Secretion	nonclassical secretion	N	0.722	N
P14618	PKM OIP3 PK2 PK3 PKM2	531	O	O	Multiple location	others	N	0.42	N
P31150	I1 GDIL OPHN2 RABGDIA X	447	O	O	Cytoplasmic	others	N	0.429	N
P50395	GDI2 RABGDIB	445	O	O	Multiple location	others	N	0.315	N
P35241	RDX	583		O	Membrane	others	N	0.31	N
Q9UN86	G3BP2 KIAA0660	482	O		Cytoplasmic	others	N	0.221	N
P46940	IQGAP1 KIAA0051	1657	O	O	Membrane	others	N	0.24	N
P63244	RACK1 GNB2L1 HLC7 PIG21	317	O	O	Membrane	others	N	0.465	N
P10586	PTPRF LAR	1907		O	Secretion	classical secretion	Y	0.419	Y
P23470	PTPRG PTPG	1445	O		Membrane	membrane integral protein	N	0.152	Y
Q15262	PTPRK PTPK	1439		O	Secretion	classical secretion	Y	0.36	Y
P23471	Z1 HTPZP2 PTPRZ PTPRZ2	2315		O	Secretion	classical secretion	Y	0.404	Y
P35244	RPA3 REPA3 RPA14	121	O	O	Nucleus	others	N	0.351	N
Q15293	RCN1 RCN	331	O	O	Secretion	classical secretion	Y	0.851	N
Q9NQC3	I KIAA0886 NOGO My043 SP	1192		O	Membrane	membrane integral protein	N	0.23	Y
P02753	RBP4 PRO2222	201	O		Secretion	classical secretion	Y	0.858	N
P52565	ARHGDIA GDIA1	204	O	O	Cytoplasmic	others	N	0.43	N
P13489	RNH1 PRI RNH	461	O	O	Secretion	nonclassical secretion	N	0.568	N
P11908	PRPS2	318	O		Secretion	others	N	0.489	N
Q9GZL7	WDR12	423		O	Secretion	nonclassical secretion	N	0.58	N
P16083	NQO2 NMOR2	231	O		Cytoplasmic	others	N	0.366	N
Q96AT9	RPE HUSSY-17	228		O	Secretion	nonclassical secretion	N	0.547	N
P98179	RBM3 RNPL	157	O		Secretion	nonclassical secretion	N	0.845	N
Q9Y230	3L2 INO80J TIP48 TIP49B C	463		O	Multiple location	others	N	0.344	N
P31153	MAT2A AMS2 MATA2	395		O	Secretion	nonclassical secretion	N	0.552	N
Q12765	SCRN1 KIAA0193	414		O	Secretion	nonclassical secretion	N	0.519	N
Q9UHJ6	SHPK CARKL	478		O	Secretion	nonclassical secretion	N	0.68	N
P49903	SEPHS1 SELD SPS SPS1	392	O		Secretion	nonclassical secretion	N	0.535	N
Q9NVA2	SEPT11	429	O		Cytoplasmic	others	N	0.069	N
Q15019	EPT2 DIFF6 KIAA0158 NEDD	361	O	O	Multiple location	others	N	0.129	N
Q16181	SEPT7 CDC10	437		O	Multiple location	others	N	0.379	N
P84103	SRSF3 SFRS3 SRP20	164		O	Multiple location	others	N	0.427	N
P30153	PPP2R1A	589	O	O	Secretion	nonclassical secretion	N	0.562	N
Q15257	PPP2R4 PTPA	358	O		Secretion	nonclassical secretion	N	0.54	N
P62714	PPP2CB	309		O	Multiple location	others	N	0.442	N
P53041	PPP5C PPP5	499	O		Multiple location	others	N	0.287	N
O00743	PPP6C PPP6	305	O		Cytoplasmic	others	N	0.497	N

P36873	PPP1CC	323	O		Multiple location	others	N	0.48	N
P49591	SARS SERS	514		O	Cytoplasmic	others	N	0.229	N
P02787	TF PRO1400	698	O	O	Secretion	classical secretion	Y	0.478	N
P35237	SERPINB6 PI6 PTI	376	O	O	Cytoplasmic	others	N	0.44	N
P50454	. CBP1 CBP2 HSP47 SERPII	418		O	Secretion	classical secretion	Y	0.864	N
P02768	PRO1708 PRO2044 PRO261	609	O	O	Secretion	classical secretion	Y	0.467	N
P10768	ESD	282	O	O	Secretion	nonclassical secretion	N	0.57	N
P55822	SH3BGR	239		O	Multiple location	others	N	0.22	N
O75368	SH3BGRL	114	O		Secretion	nonclassical secretion	N	0.758	N
Q9HAT2	SIAE YSG2	523	O		Secretion	classical secretion	Y	0.786	N
P40763	STAT3 APRF	770	O		Secretion	nonclassical secretion	N	0.521	N
Q04837	SSBP1 SSBP	148	O	O	Secretion	nonclassical secretion	N	0.85	N
P62314	SNRPD1	119		O	Multiple location	others	N	0.391	N
P62318	SNRPD3	126		O	Secretion	nonclassical secretion	N	0.763	N
Q13126	MTAP MSAP	283		O	Multiple location	others	N	0.458	N
P55011	SLC12A2 NKCC1	1212	O		Secretion	nonclassical secretion	N	0.625	Y
P30626	SRI	198	O	O	Secretion	nonclassical secretion	N	0.912	N
Q92673	SORL1 C11orf32	2214	O		Secretion	classical secretion	Y	0.534	Y
P09486	SPARC ON	303	O		Secretion	classical secretion	Y	0.942	N
Q14515	SPARCL1	664		O	Secretion	classical secretion	Y	0.378	N
Q13813	SPTAN1 NEAS SPTA2	2472	O	O	Cytoplasmic	others	N	0.237	N
Q01082	SPTBN1 SPTB2	2364	O	O	Cytoplasmic	others	N	0.177	N
P63208	P1 EMC19 OCP2 SKP1A TCE	163	O		Secretion	nonclassical secretion	N	0.648	N
Q9BWJ5	SF3B5 SF3B10	86		O	Secretion	nonclassical secretion	N	0.624	N
P23246	SFPQ PSF	707	O		Multiple location	others	N	0.318	N
P52823	STC1 STC	247	O		Secretion	classical secretion	Y	0.911	N
P31948	STIP1	543	O	O	Multiple location	others	N	0.344	N
Q15772	SPEG APEG1 KIAA1297	3267	O		Nucleus	others	N	0.315	N
P48061	CXCL12 SDF1 SDF1A SDF1E	93	O		Secretion	classical secretion	Y	0.851	N
P31040	SDHA SDH2 SDHF	664		O	Membrane	others	N	0.478	N
P55809	OXCT1 OXCT SCOT	520	O	O	Secretion	classical secretion	Y	0.496	N
O00391	OX1 QSCN6 UNQ2520/PRO6	747	O		Secretion	classical secretion	Y	0.611	Y
P63279	UBE2I UBC9 UBCE9	158		O	Secretion	nonclassical secretion	N	0.749	N
P00441	SOD1	154	O	O	Secretion	nonclassical secretion	N	0.648	N
P04179	SOD2	222	O		Secretion	nonclassical secretion	N	0.535	N
O60687	SRPX2 SRPUL	465	O		Secretion	classical secretion	Y	0.575	N
Q9Y490	TLN1 KIAA1027 TLN	2541	O	O	Membrane	others	N	0.233	N
P17987	TCP1 CCT1 CCTA	556	O	O	Secretion	nonclassical secretion	N	0.518	N
P78371	CCT2 99D8.1 CCTB	535		O	Cytoplasmic	others	N	0.446	N
P48643	CCT5 CCTE KIAA0098	541	O	O	Cytoplasmic	others	N	0.425	N
Q99832	CCT7 CCTH NIP7-1	543		O	Cytoplasmic	others	N	0.382	N
P50990	CT8 C21orf112 CCTQ KIAA0	548		O	Secretion	nonclassical secretion	N	0.508	N
P24821	TNC HXB	2201	O	O	Secretion	classical secretion	Y	0.468	Y
Q9NT68	ENM2 KIAA1127 ODZ2 TNM	2774	O		Membrane	membrane integral protein	N	0.466	Y
P52888	THOP1	689		O	Cytoplasmic	others	N	0.374	N
Q9BRA2	TXNDC17 TXNL5	123	O		Cytoplasmic	others	N	0.491	N
Q16881	TXNRD1 GRIM12 KDRF	649	O	O	Multiple location	others	N	0.359	N
P30048	PRDX3 AOP1	256	O		Secretion	nonclassical secretion	N	0.756	N
P26639	TARS	723	O	O	Cytoplasmic	others	N	0.337	N
P35442	THBS2 TSP2	1172		O	Secretion	classical secretion	Y	0.526	N
P20062	TCN2 TC2	427	O		Secretion	classical secretion	Y	0.764	N
P20290	BTF3 NACB OK/SW-cl.8	206	O		Multiple location	others	N	0.254	N
P02786	TFRC	760	O		Secretion	nonclassical secretion	N	0.611	Y
Q15582	TGFBI BIGH3	683	O		Secretion	classical secretion	Y	0.454	Y
P37802	AGLN2 KIAA0120 CDABP00	199	O	O	Secretion	nonclassical secretion	N	0.784	N
P55072	VCP	806	O	O	Multiple location	others	N	0.163	N
P29401	TKT	623	O	O	Nucleus	others	N	0.315	N
P13693	TPT1	172	O	O	Secretion	nonclassical secretion	N	0.581	N
Q15631	TSN	228	O	O	Multiple location	others	N	0.229	N
Q99598	TSNAX TRAX	290	O		Cytoplasmic	others	N	0.419	N
P22102	GART PGFT PRGS	1010		O	Secretion	nonclassical secretion	N	0.526	N
P60174	TPI1 TPI	286	O	O	Secretion	nonclassical secretion	N	0.51	N
O14773	P1 CLN2 GIG1 UNQ267/PRO	563	O	O	Secretion	classical secretion	Y	0.746	N
P29144	TPP2	1249	O		Multiple location	others	N	0.413	N
P06753	TPM3	285	O		Secretion	nonclassical secretion	N	0.533	N
P67936	TPM4	248	O		Cytoplasmic	others	N	0.417	N
Q71U36	TUBA1A TUBA3	451		O	Cytoplasmic	others	N	0.469	N
P68363	TUBA1B	451		O	Cytoplasmic	others	N	0.472	N
Q9BQE3	TUBA1C TUBA6	449	O		Cytoplasmic	others	N	0.481	N
P07437	TUBB TUBB5 OK/SW-cl.56	444	O		Cytoplasmic	others	N	0.494	N
Q9BVA1	TUBB2B	445		O	Secretion	nonclassical secretion	N	0.527	N
P68371	TUBB4B TUBB2C	445	O	O	Secretion	nonclassical secretion	N	0.501	N
Q9BUF5	TUBB6	446		O	Secretion	nonclassical secretion	N	0.544	N
Q3ZCM7	TUBB8	444	O		Cytoplasmic	others	N	0.473	N
O75347	TBCA	108	O		Cytoplasmic	others	N	0.455	N
P55327	TPD52	224		O	Secretion	nonclassical secretion	N	0.575	N
Q6IBS0	TWF2 PTK9L MSTP011	349	O		Cytoplasmic	others	N	0.418	N
O15116	LSM1 CASM	133		O	Nucleus	others	N	0.358	N
Q9Y333	LSM2 C6orf28 G7B	95		O	Secretion	nonclassical secretion	N	0.597	N
O95777	LSM8	96	O		Secretion	nonclassical secretion	N	0.532	N
Q96DE0	NUDT16	195	O		Nucleus	others	N	0.445	N
Q9UMX0	UBQLN1 DA41 PLIC1	589	O		Secretion	others	N	0.402	N
P09936	UCHL1	223	O	O	Secretion	nonclassical secretion	N	0.53	N
P62979	RPS27A UBA80 UBCEP1	156		O	Secretion	nonclassical secretion	N	0.88	N
P51668	2D1 SFT UBC5A UBCH5 UB(	147	O		Secretion	nonclassical secretion	N	0.901	N
P68036	UBE2L3 UBCE7 UBCH7	154	O	O	Secretion	nonclassical secretion	N	0.582	N
P61960	UFM1 C13orf20 BM-002	85	O		Multiple location	others	N	0.484	N
P22314	UBA1 A1S9T UBE1	1058	O	O	Secretion	nonclassical secretion	N	0.53	N
Q16222	UAP1 SPAG2	522		O	Secretion	nonclassical secretion	N	0.526	N
A6NCD4	C10orf131	256	O		Secretion	nonclassical secretion	N	0.703	N
Q9NWV4	C1orf123	160		O	Cytoplasmic	others	N	0.332	N
P06132	UROD	367	O		Secretion	nonclassical secretion	N	0.588	N
Q16851	UGP2 UGP1	508	O		Cytoplasmic	others	N	0.34	N
P54727	RAD23B	409	O		Multiple location	others	N	0.148	N
O75436	VPS26A VPS26	327	O		Cytoplasmic	others	N	0.432	N
Q9NP79	TA1 C6orf55 HSPC228 My01	307	O		Cytoplasmic	others	N	0.456	N
Q86WA6	BPHL MCNAA	291		O	Secretion	classical secretion	Y	0.85	N
P19320	VCAM1 L1CAM	739	O		Secretion	classical secretion	Y	0.605	Y
Q6EMK4	SLITL2 UNQ314/PRO357/PR	673	O		Secretion	classical secretion	Y	0.216	Y
P13611	VCAN CSPG2	3396	O	O	Secretion	classical secretion	Y	0.46	N
P08670	VIM	466	O	O	Secretion	nonclassical secretion	N	0.512	N
P18206	VCL	1134	O		Membrane	others	N	0.181	N
P07225	PROS1 PROS	676		O	Secretion	classical secretion	Y	0.5	N
Q9UPU3	SORCS3 KIAA1059	1222	O		Secretion	classical secretion	Y	0.307	Y
Q15904	P1 ATP6IP1 ATP6S1 VATPS	470	O		Secretion	classical secretion	Y	0.637	Y

O75083	WDR1	606	O	O	Secretion	nonclassical secretion	N	0.518	N
Q6UXN9	MEM113 WDR82A UNQ9342/	313		O	Nucleus	others	N	0.463	N
P12955	PEPD PRD	493		O	Nucleus	others	N	0.479	N
P13010	XRCC5 G22P2	732	O		Nucleus	others	N	0.447	N
P12956	XRCC6 G22P1	609		O	Nucleus	others	N	0.372	N
P16989	YBX3 CSDA DBPA	372	O		Secretion	nonclassical secretion	N	0.615	N
Q15942	ZYX	572		O	Secretion	nonclassical secretion	N	0.528	N

Supplementary Table 3. The mRNA expression of MDK (RSEM z-score) and overall survival (OS, months) in TCGA GBM dataset used in Fig. 1d

SAMPLE_ID	MDK_expr(RSEM zscore)	OS(month)
TCGA-02-0047-01	-1.0325	14.72
TCGA-02-0055-01	-0.336	2.5
TCGA-06-0878-01	0.3344	7.16
TCGA-06-0882-01	0.0147	20.76
TCGA-12-0821-01	1.5148	10.61
TCGA-14-0787-01	0.824	2.23
TCGA-14-0789-01	-1.0108	11.24
TCGA-14-0817-01	-0.2958	5.39
TCGA-16-0846-01	-0.6494	3.91
TCGA-06-1804-01	-0.991	13.6
TCGA-06-5408-01	0.019	11.73
TCGA-06-5410-01	-0.385	3.55
TCGA-06-5411-01	-0.609	8.34
TCGA-06-5412-01	-0.2524	4.53
TCGA-06-5413-01	-0.5189	8.8
TCGA-06-5856-01	-1.0857	3.75
TCGA-06-5858-01	-0.9553	6.14
TCGA-06-5859-01	-0.965	4.57
TCGA-14-0781-01	-0.2287	0.95
TCGA-19-5960-01	-0.9613	14.95
TCGA-32-1980-01	-0.8769	1.18
TCGA-41-5651-01	-0.9772	15.11
TCGA-16-1045-01	-1.1065	29.01
TCGA-14-1823-01	-1.2432	17.84
TCGA-14-1825-01	-0.2762	7.62
TCGA-14-1829-01	-0.3664	7.16
TCGA-27-1830-01	-0.8387	5.06
TCGA-27-1832-01	2.5636	9.86
TCGA-27-1834-01	-0.9305	40.51
TCGA-02-2485-01	0.0438	15.44
TCGA-02-2486-01	0.4047	20.3
TCGA-06-2557-01	-0.2306	1.08
TCGA-06-2558-01	-0.9616	12.48
TCGA-06-2559-01	0.0442	4.93
TCGA-06-2561-01	-0.5081	17.64
TCGA-06-2562-01	0.1315	12.55
TCGA-06-2563-01	0.3608	30.62
TCGA-06-2564-01	-0.0305	5.95
TCGA-06-2565-01	-0.4843	16.62

TCGA-06-2567-01	-0.2715	4.37
TCGA-06-2569-01	4.0789	0.43
TCGA-14-0790-01	0.2852	13.76
TCGA-14-2554-01	-0.86	17.48
TCGA-27-1831-01	2.4167	16.59
TCGA-27-1835-01	-0.3548	21.29
TCGA-27-1837-01	-0.1303	14.03
TCGA-27-2519-01	-0.6173	18.07
TCGA-27-2523-01	0.4899	16.06
TCGA-27-2524-01	1.5787	7.59
TCGA-27-2526-01	-0.8123	2.86
TCGA-27-2528-01	-0.84	15.77
TCGA-28-1747-01	-0.974	2.53
TCGA-28-1753-01	-0.3282	1.22
TCGA-28-2509-01	0.1961	4.76
TCGA-28-2513-01	1.3239	7.29
TCGA-28-2514-01	-1.2338	5.26
TCGA-32-1970-01	-0.9834	15.37
TCGA-32-1982-01	-0.3004	4.66
TCGA-06-0130-01	4.3612	12.94
TCGA-06-0141-01	0.5535	10.28
TCGA-12-3650-01	1.5345	10.94
TCGA-12-3652-01	0.8402	34.89
TCGA-12-3653-01	-0.7737	14.52
TCGA-19-1390-01	-0.2636	25.36
TCGA-19-2619-01	-1.1218	9.66
TCGA-19-2620-01	-1.0181	4.86
TCGA-19-2624-01	-0.1528	0.16
TCGA-19-2625-01	0.9599	4.07
TCGA-32-2615-01	-0.6345	15.93
TCGA-32-2632-01	-0.972	8.84
TCGA-32-2634-01	0.913	22.77
TCGA-32-2638-01	0.0676	25.16
TCGA-41-2571-01	0.6556	0.85
TCGA-06-0132-01	-1.1509	25.33
TCGA-06-0157-01	0.9979	3.19
TCGA-06-0158-01	-0.8582	10.81
TCGA-06-0168-01	-0.5847	19.65
TCGA-06-0174-01	-0.1793	3.22
TCGA-06-0178-01	-0.8201	88.07
TCGA-06-0184-01	-0.7286	69.84
TCGA-06-0219-01	1.6902	0.72
TCGA-32-4213-01	1.4928	19.84

TCGA-41-2572-01	0.5059	13.34
TCGA-41-3915-01	-0.544	11.83
TCGA-41-4097-01	-0.2855	0.2
TCGA-06-0238-01	-0.0471	13.3
TCGA-06-0644-01	2.6265	12.61
TCGA-06-0645-01	-0.8177	5.75
TCGA-06-0646-01	-0.3776	5.75
TCGA-08-0386-01	-0.6398	18
TCGA-12-0616-01	0.4504	14.72
TCGA-12-0618-01	0.4272	12.98
TCGA-12-0619-01	0.6641	34.89
TCGA-06-5414-01	-0.3685	8.97
TCGA-06-5415-01	-1.1228	8.54
TCGA-06-5418-01	0.703	2.73
TCGA-12-5295-01	0.4276	14.91
TCGA-12-5299-01	0.9397	3.22
TCGA-26-5132-01	0.0595	9.4
TCGA-26-5133-01	-1.1984	14.85
TCGA-26-5134-01	-0.5232	5.49
TCGA-26-5135-01	-0.3601	8.87
TCGA-26-5136-01	0.0355	18.96
TCGA-26-5139-01	-0.1824	1.58
TCGA-28-5204-01	0.0985	14.91
TCGA-28-5207-01	-0.3138	11.27
TCGA-28-5208-01	0.1082	17.87
TCGA-28-5209-01	-0.2803	14.52
TCGA-28-5213-01	-0.7172	31.24
TCGA-28-5215-01	-0.2062	11.01
TCGA-28-5216-01	-1.002	13.63
TCGA-28-5218-01	1.3513	5.16
TCGA-28-5220-01	-0.5255	12.75
TCGA-32-5222-01	-0.2086	19.22
TCGA-76-4925-01	0.2666	4.8
TCGA-76-4926-01	0.3499	4.53
TCGA-76-4928-01	2.1622	3.09
TCGA-76-4929-01	-0.5894	3.65
TCGA-76-4931-01	-0.4671	9.17
TCGA-06-0649-01	-0.5463	2.1
TCGA-06-0686-01	0.1357	14.19
TCGA-06-0743-01	0.4991	26.38
TCGA-06-0744-01	0.6158	46.85
TCGA-06-0745-01	2.268	7.85
TCGA-06-0747-01	2.2778	2.69

TCGA-06-0749-01	-0.5897	2.69
TCGA-06-0750-01	0.9458	0.92
TCGA-15-0742-01	0.1724	13.76

Supplementary Table 4. Statistical analysis for LDA shown on Figure 2D using ELDA software (http://bioinf.wehi.edu.au/software/elda/)

NCI827

Group 1	Group 2	Chi-sq	DF	Pr (> Chi-sq)
NT	shMDK-1	22.8	1	1.82e-06
NT	sh MDK-2	22.8	1	1.82e-06
sh MDK-1	shMDK-2	0	1	1

NCI131

Group 1	Group 2	Chi-sq	DF	Pr (> Chi-sq)
NT	shMDK-1	27.5	1	1.55e-07
NT	shMDK-2	9.5	1	2.06e-03
shMDK-1	shMDK-2	5.88	1	1.53e-02

Supplementary Table 5. List of genes used in DEG analysis in Fig. 3a

Category	Gene symbol	log2(Fold_change)	*p-value*	Benjamini
up	GDF15	3.596455717	2.2569E-135	2.5078E-131
up	FTL	0.787675007	9.0577E-116	5.0324E-112
up	B2M	0.525579511	2.6079E-107	9.6598E-104
up	FTH1	0.584209458	2.8331E-50	5.2469E-47
up	DDIT4	1.532769516	1.77411E-30	1.31426E-27
up	CHI3L1	0.620671942	1.02061E-28	6.67121E-26
up	GADD45A	0.972577219	6.49365E-20	2.32766E-17
up	CRYAB	0.65833518	3.14859E-19	1.06022E-16
up	DDIT3	1.190323408	7.47492E-19	2.30726E-16
up	RPL12	0.552068473	5.66029E-17	1.57243E-14
up	NUPR1	1.376765551	2.51033E-16	6.48715E-14
up	SDCBP	0.75113177	7.30275E-16	1.80329E-13
up	SPP1	0.512467392	2.52121E-13	4.59273E-11
up	CDKN1A	1.179502464	1.56818E-12	2.64024E-10
up	WBP2	0.916807034	3.46884E-12	5.35358E-10
up	UPP1	0.647243341	7.86111E-12	1.14938E-09
up	TRIB3	1.063013841	1.17274E-10	1.49788E-08
up	MYC	0.638019815	2.07569E-10	2.59158E-08
up	ZNFX1-AS1	0.501786497	2.26506E-10	2.79659E-08
up	ADM	1.261742147	4.70157E-10	5.33101E-08
up	SLC3A2	0.590152266	9.50334E-10	1.0353E-07
up	COX7A2L	0.512644355	3.66192E-09	3.50786E-07
up	REN	4.107949868	4.02821E-09	3.82576E-07
up	SOD2	0.766910646	1.07501E-08	9.71176E-07
up	CNBP	0.515600541	1.48634E-08	1.30049E-06
up	PCNP	0.711071097	1.58124E-08	1.37272E-06
up	RABGGTB	0.661276177	2.90263E-08	2.40702E-06
up	ZFP36L1	0.955534858	3.07361E-08	2.51132E-06
up	RPL13A	0.537484914	9.85641E-08	7.4003E-06
up	ASNS	0.85224763	1.36196E-07	1.00226E-05
up	SERPINE1	1.420671177	1.88638E-07	1.35235E-05
up	CYSTM1	0.544393427	2.77418E-06	0.000152607
up	PPP1CB	0.519179091	2.95154E-06	0.000159988
up	MSMO1	0.580405817	3.80375E-06	0.000201273
up	HNRNPC	0.551362516	8.48926E-06	0.000419256
up	SRXN1	0.714033381	1.05605E-05	0.000508002
up	DIRAS3	0.783997114	1.26322E-05	0.000599868
up	HEY1	0.675954583	1.28697E-05	0.000605969
up	IVNS1ABP	0.861737921	1.38213E-05	0.000645303
up	BHLHE41	1.079887757	2.13021E-05	0.000935608
up	EGFR	1.098834231	2.17809E-05	0.000952873
up	RGS16	1.512013043	2.63232E-05	0.001120704
up	PHGDH	0.629148841	3.29881E-05	0.001362691
up	GEM	1.090973136	4.34809E-05	0.001737984
up	DDX17	0.588135698	4.94798E-05	0.001929192
up	FOS	1.137178434	5.031E-05	0.001941127
up	BOD1	0.636536185	6.31362E-05	0.002346386
up	CTGF	1.253530042	8.75323E-05	0.003058676
up	MAP1LC3B	0.602023737	0.000140347	0.004573422
up	ATP6V1G1	0.510550562	0.00014129	0.004590691
up	STAG3L4	1.227639089	0.000178753	0.005579504

up	SAT2	0.556100128	0.000229987	0.006944621
up	DKK1	0.522935971	0.000270961	0.008029124
up	PPP1R15A	0.776135057	0.00035026	0.010005372
up	SNORD68	1.290576148	0.000398247	0.01109104
up	DHX9	0.52023389	0.000419255	0.011503104
up	MTHFD2	0.59172233	0.000501241	0.013388918
up	GNPDA1	0.592016877	0.000553275	0.014465862
up	RND3	1.303527413	0.000573874	0.014899261
up	FAM102A	1.288404595	0.000594009	0.0153503
up	RFK	0.622466956	0.000826318	0.020224762
up	RRAGD	0.632031471	0.000916935	0.021817944
up	EPB41L4A-AS1	0.853184594	0.000936971	0.022058526
up	CCNB1IP1	0.594875571	0.001008416	0.023344829
up	SF1	0.550560462	0.001086628	0.024947546
up	XBP1	0.53002795	0.001237235	0.027441427
up	LITAF	0.711535504	0.001319585	0.028639122
up	ARL4C	0.56659611	0.001369182	0.029202203
up	ETNK2	0.857667959	0.001410476	0.030025306
up	PCP4	0.62213214	0.001556363	0.032508086
up	CAMLG	0.545703414	0.001835798	0.037022475
up	UBE2G2	0.721721101	0.001945801	0.03861025
up	DUSP5	0.600070043	0.001992425	0.03925502
up	PMAIP1	0.848499034	0.002269919	0.043264734
down	HSPA8	-0.56812526	1.09353E-56	2.43027E-53
down	CCNB1	-1.468150209	1.31837E-26	6.97606E-24
down	DDX1	-0.662079779	2.12543E-24	1.02686E-21
down	ID1	-1.692992017	1.87642E-22	7.44671E-20
down	HSPA1A	-1.425500753	5.62175E-19	1.78482E-16
down	METRN	-0.673735787	6.37424E-16	1.60979E-13
down	KPNA2	-0.855723613	1.15781E-15	2.79687E-13
down	UBE2S	-0.680168881	2.71776E-15	6.29161E-13
down	ID3	-0.758356195	4.001E-15	9.07328E-13
down	FLNA	-0.953472669	1.08165E-14	2.31139E-12
down	TST	-1.987295287	1.51269E-14	3.17151E-12
down	HNRNPD	-0.762711687	2.56642E-14	5.28113E-12
down	DYNLT1	-0.713538357	2.58744E-14	5.22758E-12
down	PPP1R35	-1.193946178	1.53317E-13	2.93733E-11
down	UBE2C	-0.691637684	1.6446E-13	3.09742E-11
down	MARCKSL1	-0.646776624	1.70091E-13	3.15008E-11
down	PLK1	-2.036613496	5.45059E-13	9.6138E-11
down	NES	-0.965522551	1.80778E-12	2.99822E-10
down	FASTK	-1.562358199	1.93399E-12	3.16036E-10
down	TUBB6	-0.814290121	3.20512E-12	5.0879E-10
down	WNT7B	-1.577371418	3.28529E-12	5.1417E-10
down	AURKA	-1.506101344	1.03108E-11	1.46889E-09
down	TUBB4B	-0.523146014	1.33181E-11	1.87329E-09
down	MRPL27	-0.595410202	2.6137E-11	3.63042E-09
down	TUBB3	-0.693976032	2.72271E-11	3.73516E-09
down	H2AFX	-0.562563798	4.8237E-11	6.38107E-09
down	FAM173A	-0.918305795	1.32481E-10	1.67288E-08
down	MRPS16	-1.018260983	3.20606E-10	3.83072E-08
down	DTYMK	-0.64780297	3.29208E-10	3.89166E-08
down	RFC2	-1.183291798	3.47799E-10	4.06815E-08
down	SIVA1	-0.557749936	4.20228E-10	4.814E-08
down	UBA1	-0.773013723	4.90821E-10	5.454E-08
down	MRPL24	-0.57435338	5.28195E-10	5.81119E-08

down	REEP4	-1.527791043	1.04809E-09	1.13072E-07
down	CDC20	-1.364566571	1.3442E-09	1.42255E-07
down	SRSF1	-0.662922931	1.66926E-09	1.70173E-07
down	NDC80	-1.635274356	4.33912E-09	4.08612E-07
down	PSMG4	-1.369903218	4.62372E-09	4.31755E-07
down	PAQR4	-1.915156226	5.07679E-09	4.70111E-07
down	MBD3	-0.738797677	1.3558E-08	1.20526E-06
down	CEND1	-1.100897837	1.37621E-08	1.21369E-06
down	H1FX	-0.596384558	1.86302E-08	1.59245E-06
down	CD82	-1.02213415	2.73237E-08	2.30016E-06
down	RANGAP1	-0.711934096	3.0441E-08	2.50564E-06
down	ARHGDIA	-0.518680292	4.79041E-08	3.80222E-06
down	TRAPPC4	-0.576480199	4.82398E-08	3.8017E-06
down	CCL2	-0.730776894	5.02917E-08	3.9355E-06
down	DBNL	-0.906936355	5.43023E-08	4.21963E-06
down	PSRC1	-1.049387509	7.82919E-08	6.04153E-06
down	CCNB2	-0.952873991	8.21821E-08	6.29798E-06
down	POP5	-0.966786702	8.45789E-08	6.39347E-06
down	TOP2A	-1.38503518	1.01637E-07	7.57977E-06
down	RBMX	-0.719965233	1.42879E-07	1.04452E-05
down	CSRP1	-0.685849153	1.81372E-07	1.30871E-05
down	GAP43	-0.816531204	2.3485E-07	1.64129E-05
down	TPX2	-0.944233642	2.486E-07	1.7158E-05
down	PPM1G	-0.654936617	2.80833E-07	1.91449E-05
down	HSPA1B	-0.522719709	2.84451E-07	1.92733E-05
down	ACTN4	-1.272755429	4.43649E-07	2.8999E-05
down	MYH9	-0.941844225	4.48308E-07	2.91322E-05
down	MVD	-0.944421198	4.64015E-07	2.99775E-05
down	DUS1L	-1.356393755	4.89252E-07	3.14253E-05
down	LMNB1	-0.79521887	5.74978E-07	3.65095E-05
down	FAM129B	-0.669355762	6.10015E-07	3.85141E-05
down	SRSF6	-0.916620996	6.12553E-07	3.84558E-05
down	FARSA	-0.877149023	6.74918E-07	4.18977E-05
down	ASF1B	-1.179826276	7.12544E-07	4.37447E-05
down	CDC37	-1.099657453	7.75985E-07	4.71188E-05
down	TACC3	-1.148549767	8.74922E-07	5.22695E-05
down	SLC4A2	-1.007942189	9.74833E-07	5.76189E-05
down	CD97	-1.042491544	1.27933E-06	7.48203E-05
down	U2AF2	-1.127264157	1.36381E-06	7.89306E-05
down	MAD1L1	-1.728409079	1.40038E-06	8.06273E-05
down	TMEM101	-1.257661669	1.52507E-06	8.69058E-05
down	PVRL1	-0.966759596	1.64412E-06	9.32117E-05
down	XPO1	-0.807827861	1.67537E-06	9.45012E-05
down	PLXNA1	-1.589131438	2.36285E-06	0.00013128
down	EPB49	-0.51015539	2.92552E-06	0.000159355
down	PLEC	-1.322898541	3.29165E-06	0.000177557
down	OGDH	-1.212940536	3.53786E-06	0.000189916
down	FADS2	-0.644274194	3.7321E-06	0.00019938
down	TRIM65	-2.159929979	3.79926E-06	0.000201997
down	OLIG1	-1.144616698	4.05473E-06	0.000213536
down	SF3B3	-1.50134903	4.24086E-06	0.000222285
down	PORCN	-0.896222363	4.53173E-06	0.000236416
down	PSMD1	-0.798609299	4.64538E-06	0.000241212
down	NPDC1	-0.63421029	4.68684E-06	0.000242233
down	CBR3	-0.734775173	6.33553E-06	0.000321463
down	KIF4A	-1.285258796	7.53183E-06	0.000380426

down	PDCD6	-0.542074508	8.45798E-06	0.000419576
down	TTYH3	-1.127948152	9.42622E-06	0.000461428
down	PPP2R4	-0.697231682	1.01641E-05	0.000491057
down	CDCA3	-1.070119618	1.07336E-05	0.000511895
down	NOP58	-0.630482522	1.3263E-05	0.000621851
down	AGPAT1	-0.919459383	1.41611E-05	0.000658402
down	RBM4B	-1.711104599	1.51457E-05	0.00069545
down	RMI2	-0.910678068	1.65469E-05	0.00075356
down	METTL7B	-0.744104419	1.84745E-05	0.000837912
down	FAM83D	-1.224027032	1.85044E-05	0.000835856
down	HES6	-0.591965216	1.86107E-05	0.000837254
down	FAM20C	-1.143286179	1.94615E-05	0.000868499
down	GNL2	-1.558591858	2.1175E-05	0.000933715
down	KIF22	-0.957951314	2.22444E-05	0.000965545
down	SLC12A9	-1.424466276	2.23717E-05	0.000963542
down	WDR13	-0.917297373	2.37199E-05	0.001017666
down	KIF2C	-1.113653857	2.7289E-05	0.001157387
down	MAP7D1	-0.781510996	3.23581E-05	0.001341655
down	ATXN10	-0.612355864	3.74985E-05	0.001537578
down	TMEM129	-1.406262739	3.95602E-05	0.001604354
down	SLC2A6	-1.768490083	3.99279E-05	0.001613377
down	MCM4	-0.692848585	4.31768E-05	0.001732062
down	COQ9	-0.835080454	4.37718E-05	0.001743342
down	NR2F6	-0.777825682	4.50292E-05	0.001787015
down	C1QTNF1	-0.882885843	4.56504E-05	0.00180522
down	FAM64A	-0.863913979	4.74673E-05	0.001863803
down	CCAR1	-1.213350023	4.79921E-05	0.001877777
down	RHOB	-0.758402861	5.01888E-05	0.001943198
down	THOP1	-1.03649577	5.44549E-05	0.002079393
down	USP5	-0.818436115	5.66591E-05	0.002148793
down	PARP12	-1.590569969	5.98865E-05	0.002240601
down	MRPL38	-0.667739536	6.35131E-05	0.002352524
down	FTSJ1	-0.546942961	7.12185E-05	0.002620463
down	OXLD1	-0.91884636	7.14939E-05	0.002621916
down	MRPL1	-0.690838771	7.29778E-05	0.002658786
down	SMOC1	-0.692896135	7.82654E-05	0.002823654
down	TWF2	-0.521752779	7.99142E-05	0.002873807
down	NCAPD2	-1.424861076	8.10962E-05	0.002906905
down	TXNIP	-0.626225898	8.38462E-05	0.002976673
down	CHORDC1	-1.195024142	8.49776E-05	0.003007232
down	TMEM165	-1.053752485	8.52197E-05	0.003006226
down	SPAG5	-1.376622118	8.55171E-05	0.003007173
down	OBSL1	-1.314220489	8.81803E-05	0.00307166
down	NTHL1	-0.597128266	8.89677E-05	0.003089404
down	AKT1	-0.811838437	8.9397E-05	0.003094641
down	ATIC	-0.551594537	0.000102483	0.00351478
down	MEPCE	-0.829852551	0.000102793	0.003514576
down	CDKN2C	-0.834397554	0.000104473	0.003561055
down	APOBEC3C	-1.413623576	0.000108951	0.003679844
down	CENPA	-1.624567917	0.000110889	0.003733919
down	SAFB	-0.862830156	0.000112107	0.003763539
down	CHD4	-0.886621002	0.000117724	0.003940195
down	NPTX2	-1.954372768	0.000122658	0.004093035
down	HNRPDL	-0.754151393	0.000122935	0.004077756
down	MAGED1	-0.505174707	0.000132897	0.004382046
down	MPG	-0.632900852	0.000134367	0.00441743

down	SIGMAR1	-1.163270153	0.00014444	0.004679355
down	LRRC45	-1.606101979	0.000159108	0.005080492
down	ITPA	-0.500580507	0.000169002	0.005365559
down	DAB2IP	-1.131037632	0.000171002	0.00539822
down	CCNF	-1.603637226	0.000177678	0.005577294
down	SDSL	-0.917702092	0.00017869	0.005593261
down	CHMP6	-1.186298846	0.000185064	0.005760309
down	ANAPC2	-1.850292644	0.000203131	0.006287428
down	POLD4	-0.729968004	0.000209795	0.006457743
down	B3GAT3	-0.62722063	0.000212957	0.006536945
down	SAFB2	-1.830668896	0.000215422	0.006594408
down	TCF25	-0.820735988	0.000215693	0.00658457
down	LRRC17	-1.089364313	0.000228096	0.006925154
down	OCIAD1	-0.6419677	0.000228299	0.006912417
down	SLC38A5	-0.504929247	0.000230982	0.006955746
down	NUDT22	-0.775978952	0.00023394	0.007025796
down	UNC50	-0.68222831	0.000235088	0.007041245
down	PYCR2	-0.558539968	0.000269406	0.008004369
down	SREBF1	-1.396771319	0.000283649	0.008360486
down	SLC27A4	-1.046551434	0.000312737	0.009097214
down	MAVS	-1.271194751	0.00032261	0.00931127
down	FAM165B	-0.570318446	0.000330348	0.00950991
down	VARS	-1.030412068	0.000366379	0.010385732
down	IRF3	-1.175488215	0.000370269	0.010469272
down	HEATR2	-1.12633273	0.000372707	0.010484849
down	RACGAP1	-0.771959071	0.000375686	0.01054197
down	DNMT1	-1.335857596	0.000394692	0.011019643
down	SRSF11	-0.731798034	0.000421829	0.011545235
down	SDHA	-0.711833277	0.00042421	0.011581858
down	NGEF	-0.93097441	0.000426821	0.011624595
down	HYOU1	-0.813744803	0.000464283	0.012583191
down	VASP	-0.64571036	0.000465903	0.01259639
down	CDH2	-0.616290765	0.000466957	0.012594237
down	RUVBL1	-0.5027617	0.000467468	0.012577505
down	RRP36	-0.654621956	0.000484548	0.013005558
down	NCLN	-0.838196478	0.000501199	0.013420066
down	AURKB	-0.677508233	0.000506758	0.013503833
down	ZDHHC16	-1.293690562	0.000510827	0.013579692
down	MUS81	-1.711955059	0.000511675	0.013569759
down	DGCR2	-1.247213066	0.00052113	0.013754864
down	TMEM43	-0.769484065	0.000522811	0.013766543
down	DLGAP5	-1.505646566	0.00060617	0.015628205
down	PKMYT1	-1.296128498	0.000611166	0.015720544
down	ABCB6	-1.475659493	0.00061892	0.01584663
down	SMC4	-0.769522796	0.000620552	0.015851895
down	KIFC1	-1.3054727	0.000636014	0.016209617
down	CDC45	-1.355000963	0.000667002	0.016921747
down	CRMP1	-0.987588787	0.000676191	0.017115804
down	TYRO3	-1.077288348	0.000677096	0.017099745
down	ANAPC7	-0.986255205	0.000714822	0.017970811
down	SLC25A1	-0.577109445	0.000719619	0.01800993
down	STOML1	-1.470443039	0.000721416	0.018014336
down	DBN1	-0.747488862	0.000757397	0.018828178
down	CCNA2	-0.793454753	0.0007657	0.018992101
down	NT5DC2	-0.731505533	0.000808826	0.019928323
down	SYMPK	-1.409339822	0.000820402	0.020168821

down	IDH1	-0.576294126	0.000861145	0.020938818
down	TSPAN18	-1.165879118	0.000865632	0.021001968
down	ARRDC1	-1.391531588	0.000872958	0.021087638
down	AP1B1	-0.906315942	0.00089968	0.021545783
down	MRPL9	-0.525387097	0.000915011	0.021818885
down	TRADD	-1.774633534	0.000930377	0.022043388
down	MRM1	-1.850981124	0.000952542	0.022377685
down	LIMK1	-1.090713817	0.000956462	0.022375181
down	SND1	-0.610503886	0.000960859	0.022430802
down	SAAL1	-0.924471224	0.000998612	0.023214591
down	ACTN1	-0.601201998	0.001020885	0.023584365
down	TPGS1	-0.743621775	0.001090614	0.02498743
down	DEPDC1	-1.332681738	0.001101758	0.025139076
down	PYCRL	-2.419139435	0.001130673	0.025745976
down	FASN	-0.950375361	0.00116871	0.026395752
down	GTF3C5	-0.572487228	0.001196286	0.026854798
down	ZFYVE19	-1.018736937	0.001197101	0.026818928
down	CDKN2AIPNL	-0.591398866	0.001201456	0.026862326
down	ARF3	-0.614276164	0.001246172	0.027584581
down	SHMT1	-1.29009219	0.001246973	0.027547448
down	LIPG	-1.191569421	0.001280727	0.028069904
down	NCOA5	-1.192684308	0.001285996	0.028129893
down	CHTF8	-0.816908797	0.001297079	0.028316577
down	ANKRD52	-1.595416887	0.001299509	0.028314018
down	IVD	-1.18468546	0.001302465	0.028322886
down	NDOR1	-1.772147697	0.001319951	0.028591215
down	PRR11	-1.135115689	0.001341425	0.028943521
down	APEH	-0.625195921	0.001347612	0.028964532
down	POLA2	-1.27379517	0.001349019	0.028938791
down	H1F0	-0.543447805	0.001358492	0.02902993
down	OIP5	-0.774269797	0.001420759	0.030186373
down	TMEM42	-0.963143451	0.001490399	0.031425636
down	SCRIB	-0.796445552	0.001509737	0.031773106
down	KIF20A	-2.048411436	0.001556948	0.032459289
down	KIF11	-0.976629957	0.001611862	0.033541218
down	ZWINT	-0.62188351	0.001617899	0.033603916
down	TELO2	-1.112518331	0.001644245	0.03402392
down	NUF2	-0.928334483	0.001651511	0.034047477
down	STAT6	-1.63177822	0.001698123	0.034879015
down	TH1L	-0.81353976	0.001765409	0.035928993
down	KPNB1	-0.650623769	0.001771373	0.035984461
down	ARL16	-0.544653985	0.001810417	0.036643637
down	MATR3	-0.526521689	0.001823554	0.03684243
down	LSS	-0.949157416	0.001854277	0.037327407
down	FLYWCH2	-0.83747957	0.001864999	0.037475358
down	GPS2	-0.837117645	0.001870354	0.037515106
down	CDCA8	-1.001850219	0.00188897	0.037752215
down	SLC25A13	-1.01884016	0.001902082	0.037946018
down	GYS1	-1.203594684	0.001907661	0.037989118
down	MYO1C	-1.515928914	0.001938558	0.038535352
down	ZNF771	-1.258413232	0.001951184	0.038648043
down	DDX19A	-0.929166909	0.001984642	0.039171132
down	GAS2L1	-0.92231321	0.002020766	0.039742931
down	SLC9A3R2	-0.553482079	0.002090179	0.041035463
down	GCN1L1	-1.35657563	0.002102165	0.041197987
down	PARP10	-1.274293884	0.002161106	0.042130195

down	LRP11	-0.730847266	0.002193104	0.042679103
down	AIF1L	-0.552572122	0.002196554	0.042597048
down	HK1	-0.575494215	0.002196827	0.042528112
down	CCDC22	-1.076150362	0.002203253	0.042578344
down	RPRML	-0.79207035	0.002203572	0.042510573
down	NOS2	-2.419015464	0.002205114	0.042466598
down	PRPF31	-0.743246563	0.002216049	0.042603347
down	ALG8	-0.911745931	0.002282222	0.043424736
down	NOG	-1.036275966	0.002316715	0.043930616
down	BUB1	-0.9278213	0.002428384	0.045735945
down	PRPF6	-0.637556852	0.002437376	0.04567305
down	TNKS1BP1	-1.219942012	0.002485964	0.046505112
down	IPO4	-1.21710291	0.002516056	0.046988938
down	LFNG	-1.013956058	0.002644423	0.049056482
down	FAM216A	-0.79607012	0.002693851	0.04989012

(Benjamini et al. 1995)

Supplementary Table 6. Information of selected gene-set demonstrated in Fig. 3b

Category	Accession	Description	Count	*p-value*	Genes	List Total	Pop Hits	Fold Enrichment	Benjamini	FDR
Up-regulated	GO:0006979	Response to oxidative stress	7	9.3.E-05	EGFR, FOS, CRYAB, SERPINE1, DDIT3, SRXN1, SOD2	62	164	9.3.E+00	2.8.E-02	1.4.E-01
Up-regulated	GO:0007568	Aging	6	1.3.E-04	FOS, ADM, CRYAB, SERPINE1, DDIT3, SOD2	62	110	1.2.E+01	2.4.E-02	2.1.E-01
Up-regulated	GO:0007050	Cell cycle arrest	5	1.2.E-03	CDKN1A, PPP1R15A, GADD45A, MYC, DDIT3	62	103	1.1.E+01	9.4.E-02	1.8.E+00
Up-regulated	GO:0042127	Regulation of cell proliferation	11	2.5.E-03	EGFR, CDKN1A, CNBP, NUPR1, ADM, SERPINE1, SF1, PPP1CB, MYC, FTH1, SOD2	62	787	3.0.E+00	1.4.E-01	3.9.E+00
Up-regulated	GO:0006974	Response to DNA damage stimulus	7	6.5.E-03	CDKN1A, NUPR1, PPP1CB, PPP1R15A, GADD45A, DDIT3, SOD2	62	373	4.1.E+00	2.7.E-01	9.7.E+00
Up-regulated	GO:0008637	Apoptotic mitochondrial changes	3	8.6.E-03	PMAIP1, MYC, SOD2	62	31	2.1.E+01	3.2.E-01	1.3.E+01
Up-regulated	GO:0006915	Apoptosis	8	1.8.E-02	LITAF, TRIB3, PMAIP1, PPP1R15A, GADD45A, MYC, SOD2, DDIT4	62	602	2.9.E+00	4.5.E-01	2.5.E+01
Down-regulated	GO:0000280	Nuclear division	32	1.7.E-21	MAD1L1, KIFC1, KIF22, PKMYT1, AURKA, AURKB, FAM83D, KIF2C, CDCA8, OIP5, BUB1, CCNA2, TUBB3, CDCA3, ANAPC2, KIF11, DLGAP5, CCNF, TPX2, NUF2, CDC20, NDC80, UBE2C, SMC4, NCAPD2, CCNB1, CCNB2, SPAG5, PLK1, ZWINT, RUVBL1, ANAPC7	206	220	9.6.E+00	2.7.E-18	2.9.E-18
Down-regulated	GO:0007067	Mitosis	32	1.7.E-21	MAD1L1, KIFC1, KIF22, PKMYT1, AURKA, AURKB, FAM83D, KIF2C, CDCA8, OIP5, BUB1, CCNA2, TUBB3, CDCA3, ANAPC2, KIF11, DLGAP5, CCNF, TPX2, NUF2, CDC20, NDC80, UBE2C, SMC4, NCAPD2, CCNB1, CCNB2, SPAG5, PLK1, ZWINT, RUVBL1, ANAPC7	206	220	9.6.E+00	2.7.E-18	2.9.E-18
Down-regulated	GO:0000087	M phase of mitotic cell cycle	32	3.0.E-21	MAD1L1, KIFC1, KIF22, PKMYT1, AURKA, AURKB, FAM83D, KIF2C, CDCA8, OIP5, BUB1, CCNA2, TUBB3, CDCA3, ANAPC2, KIF11, DLGAP5, CCNF, TPX2, NUF2, CDC20, NDC80, UBE2C, SMC4, NCAPD2, CCNB1, CCNB2, SPAG5, PLK1, ZWINT, RUVBL1, ANAPC7	206	224	9.4.E+00	2.3.E-18	5.0.E-18
Down-regulated	GO:0022402	Cell cycle process	44	6.0.E-19	MAD1L1, KIF22, KIFC1, PKMYT1, AURKA, AURKB, AKT1, FAM83D, KIF2C, CDCA8, OIP5, CDKN2C, CENPA, PSMD1, BUB1, H2AFX, LFNG, CCNA2, TUBB3, CDCA3, ANAPC2, KIF11, DLGAP5, CCNF, TPX2, NUF2, CDC20, NDC80, MYH9, RACGAP1, UBE2C, TACC3, SMC4, NCAPD2, CCNB1, PPM1G, CCNB2, SPAG5, PLK1, ZWINT, GAS2L1, ANAPC7, RUVBL1, KPNA2	206	565	5.1.E+00	1.3.E-16	1.0.E-15
Down-regulated	GO:0051301	Cell division	27	3.9.E-13	MAD1L1, KIFC1, AURKB, FAM83D, CDCA8, OIP5, BUB1, CCNA2, CDCA3, ANAPC2, KIF11, CCNF, NUF2, CDC20, NDC80, MYH9, UBE2C, RACGAP1, SMC4, NCAPD2, CCNB1, CCNB2, SPAG5, PLK1, ZWINT, RUVBL1, ANAPC7	206	295	6.0.E+00	6.7.E-11	6.5.E-10
Down-regulated	GO:0051726	Regulation of cell cycle	20	6.8.E-07	XPO1, MAD1L1, ANAPC2, DLGAP5, PKMYT1, TACC3, UBE2C, CDC37, SCRIB, AKT1, CCNB1, CDC45, CDKN2C, ZWINT, BUB1, RHOB, H2AFX, ID3, CCNA2, HSPA8	206	331	4.0.E+00	9.6.E-05	1.1.E-03
Down-regulated	GO:0007059	Chromosome segregation	8	2.2.E-04	KIFC1, ZWINT, DLGAP5, NUF2, NDC80, TOP2A, NCAPD2, SMC4	206	81	6.5.E+00	2.1.E-02	3.7.E-01

Supplementary Table 7. Information of each gene-set used in ssGSEA on Fig. 3c

Description	Category	Gene list
KEGG_BASE_EXCISION_REPAIR	DNA repair	NEIL2, MPG, SMUG1, XRCC1, POLE4, HMGB1, POLE3, POLD4, MBD4, OGG1, UNG, POLD3, PCNA, NEIL1, POLE2, PARP4, PARP3, PARP2, POLB, APEX1, POLL, POLD1, POLD2, POLE, NEIL3, TDG, APEX2, LIG3, HMGB1P1, NTHL1, HMGB1P40, FEN1, LIG1, MUTYH, PARP1
REACTOME_DNA_REPAIR	DNA repair	RAD50, CDK7, CCNO, MAD2L2, POLD3, ERCC8, ALKBH2, DDB1, DDB2, ERCC1, ERCC2, ERCC3, ERCC4, ERCC5, ERCC6, FANCA, FANCC, FANCD2, FANCE, FANCB, FANCF, FANCG, ALKBH3, FEN1, SMUG1, XRCC6, ZBTB32, UBE2T, GTF2H1, GTF2H2, GTF2H3, GTF2H4, H2AFX, APEX1, LOC389901, LIG1, LIG3, LIG4, MGMT, MNAT1, MPG, MRE11A, MUTYH, NBN, ATM, NTHL1, OGG1, PCNA, REV1, POLB, POLD1, POLD2, POLE, POLE2, POLH, POLR2A, POLR2B, POLR2C, POLR2D, POLR2E, POLR2F, POLR2G, POLR2H, POLR2I, POLR2J, POLR2K, POLR2L, ATR, FANCL, TDP1, PRKDC, XAB2, FANCM, POLD4, RAD23B, RAD51, RAD52, REV3L, RFC2, RFC3, RFC4, RFC5, RPA1, RPA2, RPA3, RPS27A, LOC648152, LOC651610, LOC651921, LOC652672, LOC652857, GTF2H2B, BRCA1, BRCA2, TCEA1, TDG, TP53BP1, RPS27AP11, UBA52, USP1, XPA, XPC, XRCC1, XRCC4, XRCC5, PALB2, C17orf70, BRIP1, MBD4, CCNH, C19orf40, MDC1
SANSOM_APC_TARGETS_REQUIRE_MYC	Proliferation	NRF1, CSDA, GTPBP6, NHP2, MVK, METTL2B, DRG2, QTRTD1, DUS3L, WDR34, ADAT1, BCL11A, RRP9, RPS10, HOMER1, RPS13, CITED1, FHOD1, NFS1, GIPC1, VARS, TP53RK, PHGDH, C16orf88, SIVA1, ZMYND19, CCDC94, WDR4, KAT2A, MBD3, BUB1B, C4orf43, RNASEH2C, CAD, ATMIN, QTRT1, BIVM, TIMELESS, DDAH1, PABPC4, C7orf36, NEUROG3, SOX4, ZNF295, CENPP, D2HGDH, WDR62, ZFP2, ZNF444, HOXA9, CCDC163P, SNHG3, SLC29A2, SRM, OGG1, STOML1, ALKBH2, WRB, HES6, RAD23A, CCDC86, TMEM209, SMYD5, WHSC1, PPP1R14B, C17orf49, SIGMAR1, MED22, OGFOD1, SETD8, MYC, COX4NB, ATG9B, SEMA3B, BMP7, ZNF213, HAGHL, THOP1, ERCC1, FLYWCH2, MYEF2, RANGRF, RAI14, SLC35B2, GAR1, IFFO2, SRRD, MRPL38, GALNT10, CINP, TMEM186, WDR48, SHMT2, SYTL1, HIRA, KRTAP21-1, CDK4, DTL, CTBS, TRDN, DHX58, ZSWIM3, C10orf107, SHPRH, FADS2, SNRPF, DDX49, ARHGEF12, PTOV1, NSUN5, SNRPG, AAAS, DEPTOR, RNASEH2A, CDC42BPA, PTHLH, METTL13, ROR2, EXOSC1, NBEAL2, MUTYH, NME1, GPSM1, SRGAP2, FZD6, AXIN2, TMPO, TSEN2, EXOSC4, RAD54L, LRP8, FAM116B, NFKB2, TP53, SLCO3A1, USP20, LANCL2, SLC12A6, POLE, TNFRSF12A, CCDC166, TRIB1, WDR54, RASSF4, VEZT, PPAN, DPH2, CREBBP, ACER2, ARX, MCM7, RGS12, ZBTB48, PCBP4, TK1, FADS3, DTYMK, TXNRD2, LFNG, GAS8, GNPNAT1, POLR1A, C9orf140, METTL1, HSPBAP1, FARSB, TELO2, TTC27, TGIF2, SNX24, SHMT1, TXNRD3, TMEM180, AQR, EPHB2, DNMT3B, GTSE1, MDN1, PMF1, BOD1L, KIAA1456, RABEP2, ZNF800, NOP56, TRMT61A, FANCA, LMNB2, LHPP, OSR2, SOX17, ERCC4, ZBED3, ASCL2, POMT1, BCL11B, FBL, SETD4, MFSD3, LDB1, C16orf5, POFUT1, GTPBP3, MUS81, MAPK7, PRSS41, SKA1, CCT7, METTL11A, PMM1, BID
DANG_REGULATED_BY_MYC	Proliferation	SMN1, TRAF2, SNRPD3, SREBF1, TFRC, TOP1, UBE2C, TIMM10, VARS, TIMM23B, TLE4, CLPB, TFDP1, TDP1, SURF6, UNC119, TXNRD1, SRPK1, SNRNP70, ZNF239, EFTUD2, TXN, SLC20A1, SYNGR1, SEPHS2, UMPS, UXT, TXNL4A, STAT5B, SRM, SRSF1, WDR3, SRSF2, SFXN1, TK1, ZNF532, UCHL1, SLC7A5, THRA, VDAC1, THOP1, RRP9, SLC25A3, SHMT1, CCNC, SNRPB, UCK2, UAP1, ZNF330, SUMO2, FEZ2, SRSF7, WDR12, SLC25A4, SLC16A1, SLC6A1, TCF3, VRK1, TBL3, TERT, TARBP1, TYMS, SNRPD2, UCN2, SERPINE1, GGH, RUVBL2, YWHAE, HOMER1, VARS1, SLC2A1, TP53
ROSTY_CERVICAL_CANCER_PROLIFERATION_CLUSTER	Proliferation	AURKA, ECT2, HELLS, FOXM1, ESPL1, PAFAH1B3, PLEK2, KIF15, ASF1B, DNMT3B, TMPO, EZH2, C18orf10, BIRC5, COMMD8, MCM4, GINS4, TOP2A, NUSAP1, POLQ, DTL, RPA3, RRM2, TTK, RACGAP1, CCNA2, HN1, CDK1, DBF4, CDC20, HMGA1, MKI67, CENPF, CCNB2, TPX2, FANCI, FEN1, KIF20B, NETO2, CDCA8, MAPK13, TACC3, KPNA2, CCDC99, KIF11, KIF4A, LMNB1, BUB1B, NCAPH, GGH, EIF4EBP1, BRCA1, TYMS, STIL, SAC3D1, BUB1, CHEK1, CELSR3, CDKN2A, PLK1, TRIP13, CENPE, SLMO2, PAQR4, MLF1IP, BID, PRC1, POLA2, ATAD2, CA2, CENPA, NEK2, SHCBP1, APOBEC3B, MCM2, LRP8, CCNE2, RAD51AP1, NDC80, KIF20A, E2F8, CKS2, HSPB11, NCAPG, CENPM, PCNA, CDC6, ACACA, KIAA0101, H2AFZ, TK1, TMSB10, ANP32E, OIP5, ZWINT, DHFR, CDCA3, H2AFX, MELK, GINS1, EBP, KIF18B, MRPS15, DLGAP5, KIF23, SLC38A1, HMMR, SMC2, DNA2, SMC4, UBE2S, PBK, MYBL2, CCNB1, GMNN, PTTG1, GTSE1, DSG2, E2F1, DTYMK, MCM10, SPAG5, KIF2C, HJURP, ERCC6L, AURKB, CCDC109B, FBXO5, MAD2L1, KIF14, CEP55, UBE2C, ASPM, CCNF, HMGB2, DPP3, KIFC1, CHAF1B, SLC25A15, LSM4
KEGG_PHOSPHATIDYLINOSITOL_SIGNALING_SYSTEM	Proliferation	PLCB2, CALM2, INPP1, PLCB1, PLCD1, PRKCB, PRKCA, PLCB3, CALM1, PLCB4, DGKZ, INPP5A, DGKE, INPP5B, DGKD, INPP4A, PRKCG, INPPL1, DGKH, INPP5D, SYNJ2, PIP4K2B, ITPR3, PLCD3, PIKFYVE, IMPA1, IMPA2, PIP5K1A, PIP5K1B, INPP5J, CDS1, INPP5K, SYNJ1, PLCD4, ITPKA, PIK3R5, ITPKB, ITPK1, CDIPT, ITPR1, ITPR2, DGKQ, CDS2, INPP4B, DGKB, DGKG, DGKA, DGKI, PLCE1, CALML5, PIP5K1C, PIK3C3, PIK3C2B, PIK3C2G, PIP4K2A, PIK3C2A, PTEN, PIK3R3, PIP4K2C, IPPK, CALML3, PIK3CA, PIK3CB, PIK3CD, PLCG1, PLCG2, PLCZ1, PI4KB, CALM3, INPP5E, PIK3CG, PIK3R1, OCRL, PIK3R2, CALML6, PI4KA
ZEMBUTSU_SENSITIVITY_TO_MITOMYCIN	Proliferation	CPXM1, SHC2, PCDH1, COX6A2, TBC1D7, TRAF2, RND3, ZNF831, MMP3, OTC, PGLYRP1, S100A4, KRCC1, C7orf62, ARHGAP26, TNFRSF14, IL32, AMPD1, TRMT11
CHIANG_LIVER_CANCER_SUBCLASS_PROLIFERATION_UP	Proliferation	AURKA, CCNE1, DEPDC1B, C4orf7, CD24, SLC16A3, CDC7, SLAMF8, FOXM1, MTMR2, ELOVL7, SMC4, LDLRAD3, CDK1, SLC7A7, BUB1B, CTBP2, SLC39A10, GALNT7, TPX2, KIF14, OIP5, PRR11, MCM2, SGOL2, RAD51AP1, BIRC5, CDCA7L, DBN1, G6PD, CDCA5, CENPF, PTP4A3, MAD2L1, TMEM51, PAPLN, PAFAH1B3, FEN1, DDR1, TTF2, SKA1, FBXO5, SLC38A1, WASF1, KIF20A, LRRC1, PRKCD, BACE2, UGCG, DLGAP5, KIF2C, SLC1A5, ARHGAP18, SALL2, ANLN, SOX9, ZNF532, NCK2, LEPREL4, FMNL2, B3GNT5, PKM2, CCNB1, MEP1A, LHFPL2, TUBA4A, H19, KIF4A, MARCKS, ASRGL1, KIF11, TRIP13, MARCH3, DTL, CENPE, NUF2, CSDA, CDKN3, SOX4, CMTM3, RMI2, DSCC1, TTK, CCNA2, KIF18B, FLVCR1, ZWINT, PNMA1, PKDCC, SHCBP1, HIST1H4C, NCEH1, PAG1, CCNB2, MMP12, ABCC1, PM20D2, ASPM, FAM164A, SEL1L3, MKI67, NEK2, CKAP4, BCAT1, TMED3, PLP2, AFP, LAMB1, B4GALT5, EZH2, MAPK13, CHST11, C11orf93, WSB1, NCAPG, S100P, ORC6, TMEM65, HJURP, TNFRSF21, CDC20, MARCKSL1, SYNJ2, SLC12A3, CKAP2L, PTTG1, DUSP9, HDAC2, PIGAP1, CDCA7, GPD1L, ECT2, FAM118A, FHOD3, BARD1, DEPDC1, PBK, KIF23, MMD, RACGAP1, HN1, SASS6, NUSAP1, NDC80, SELM, PRC1, HMGB2, UBE2C, SPHK1, ELF4, NT5DC2, CDC6, FUNDC1, ZNFX1-AS1, PELI1, CCDC109B, CYBA, ATP1A1, GLIS2, LMNB1, SALL4, CEP55, RFC4, PLBD1, ARID3A, TOP2A, MECOM, TRNP1, FANCI, AURKB, HK2, ETV4, E2F8, MMP9, VEGFB, IGF2BP3, HELLS, PDE9A
EGUCHI_CELL_CYCLE_RB1_TARGETS	Cell cycle	CDC25C, ECT2, SMC4, BUB1, CCNA2, MCM5, KIF18A, MCM7, CENPE, MCM2, MCM4, NDC80, MCM6, MCM3, HMGB2, FEN1, CDCA8, NEK2, CCNE2, NCAPG, KIF20A, SMC2, KIF11
PID_AURORA_B_PATHWAY	Cell cycle	PPP1CC, KIF23, NCAPH, SGOL1, STMN1, AURKC, MYLK, SMC2, RHOA, BIRC5, KIF2C, NCL, CBX5, CDCA8, SEPT1, VIM, DES, KLHL9, RACGAP1, AURKA, INCENP, PSMA3, NSUN2, NCAPD2, RASA1, KLHL13, BUB1, NDC80, SMC4, NCAPG, AURKB, KIF20A, TACC1, PPP2R5D, PEBP1, CENPA, EVI5, CUL3, NPM1
WHITFIELD_CELL_CYCLE_G2	Cell cycle	H2AFX, TOP2A, KBTBD2, KIF22, SAP30, CCNA2, TNPO2, GPR126, TRIM69, CDKN2C, NFIC, ANLN, VPS25, DET1, CEP350, GABPB1, MND1, MUC1, CIITA, NNMT, LIX1L, BRD8, SKA3, ARMC1, BUB3, STIL, HMGB2, FAM72B, NCAPH, HJURP, ASXL1, CXCL14, DCAF7, CDK1, STAT1, SRGAP2P1, NLRP2, HIST3H2A, NR3C1, GAS1, C9orf100, C2orf69, RANGAP1, C5orf49, MAD2L1, FAM83D, MELK, NUCKS1, IQGAP3, G2E3, CCDC107, KIF11, FAN1, RGS3, SV2B, NMB, KIFC1, CASP3, CYTH2, MIS18BP1, WSB1, KIAA1524, HLA-DRA, ENTPD5, TUBB, KDM4A, KPNA2, KLF6, KIF5B, UBXN11, FZR1, FAM113A, ZNF587, MGAT2, AURKB, TFAP2A, WDR62, CFLAR, HSPA2, LMNB1, CBX5, KIF20B, SORL1, ARL4A, MALAT1, UBE2C, MET, PSRC1, NDC80, AP3D1, ATL2, NBPF10, MID1, CKAP2, WISP1, KATNA1, SGCD, PSMD11, EMP1, SMC4, BTNL9, TTF2, SUCLG2, TUBB4B, TUBD1, TRAIP, TRMT2A, HINT3, NIPBL, HP1BP3, NCOA5, CDC16, CCNF, GAS2L3, HN1, CENPL, IFNAR1, ESPL1, POLQ, PIF1, HIPK2, FAM110A, ARHGAP19, DHX8, C12orf32, STK17B, C15orf29, CDC25C, BORA, NEIL3, CKAP2L, LOC645739, CDCA3, CDR2, ARHGAP11B, TUBB2A, KIF23, LOC441052, ALKBH1, FANCD2, PKNOX1, RDH11, LTBP3, TUBA4A, CDKN1B, CDCA8, CDCA2, NUSAP1, PPP1R2, HAUS8, TYSND1, UNC5CL, UACA, FADD, CCDC165, C6orf103, HRSP12, DNAJB1, KCTD9, RCCD1, NUMA1, TMPO, LBR, VTA1, TUBA1A, RNF141, NCAPD3, CDKL5, TMEM99, ZNHIT2, EBI3, CFD, CHEK2, TRIM59, C14orf80, STAT5B, CDC42EP4, CYB5R2, TIMP1, TTC38, BCLAF1, MEPCE
ZHOU_CELL_CYCLE_GENES_IN_IR_RESPONSE_6HR	Cell cycle	PAICS, E2F8, CDT1, MAGOHB, GINS3, CENPK, HAUS8, HMGB3, RTKN2, UHRF1, LBR, RFC4, E2F1, MCM7, DCTPP1, MCM3, TPRKB, GPN3, SMC2, DNAJC9, POC1A, CCNA2, KIF22, FABP5, DEK, PSMC3IP, RPA2, USP1, LMNB1, FBXO5, MCM5, F10, GMNN, CENPA, CHAF1B, KIAA0101, KIF11, RACGAP1, KIFC1, RFC5, FAM64A, TTK, SULF2, TRIP13, MAD2L1, DTL, TOPBP1, C12orf48, KIF20A, NCAPH2, C16orf61, UBE2T, KNTC1, EXO1, WDHD1, RAD51, NUP88, CDCA5, SAP30, NEK2, BRCA1, DLGAP5, FAM54A, NUSAP1, HMGB2, QSER1, CDK2, MYBL2, MCM2, EZH2, GINS2, MCM6, HAUS3, CEP55, RAD54L, DTYMK, DCLRE1A, SEMA3B, RANBP1, H2AFX, FAM198B, TIMELESS, DSN1, SOD3, CDCA8
ZHOU_CELL_CYCLE_GENES_IN_IR_RESPONSE_24HR	Cell cycle	HAUS3, CLN6, KPNA2, NUP88, CKAP2, PAICS, THOC4, SULF2, TPRKB, TOP2A, FABP5, NEK2, SQRDL, PTTG1, LBR, MCM7, FBLN1, EIF2S1, F10, DCTPP1, SEMA3B, RPA2, LMNB1, CENPN, ASPM, C12orf48, TTK, PTTG3P, PTTG2, FGFR1OP, WIPI1, ANLN, LAMB1, C15orf23, RACGAP1, TDP1, TOPBP1, BORA, MAD2L1, CHAC2, CDC20, GPN3, HMGB2, CENPA, POLD1, H2AFV, DCN, CCNB1, USP1, ODF2, SPAG5, CDCA8, KIF20A, CACYBP, RAD54L, POC1A, NUP107, SOD3, MAGOHB, SPC25, CEP55, DEPDC1, COLEC12, CCNB2, UHRF1, C16orf61, UBE2C, KNTC1, DLGAP5, FAM64A, H2AFX, E2F8, RANBP1, EZH2, NUF2, PLEK2, NCAPH2, CCNA2, MXRA5, SMPD4, MCM3, MELK, DNAJC9, MCM8, CDKN3, FBXO5, LRRN4CL, CDK2, CDK1, MCM2, RBBP8, HAUS8, CENPF, KIFC1, QSER1, NASP, TRIP13, IFT20, CSE1L, KIF23, FAM198B, NEIL3, MDC1, HMMR, PCNA, BUB1, HMGB3, NUSAP1, MYBL2, NUP35, GINS3, EXO1, BIRC5, EXOSC8, FAM54A, TIMELESS, RFC5, PRC1, SMC2, KIF22, E2F1, CENPK, HN1, KIF11, DTYMK, LXN, RFC4, UBE2T
WHITFIELD_CELL_CYCLE_G2_M	Cell cycle	ZNF521, HMGB3, CDC27, PRR11, TOMM34, RBM8A, RCAN1, MIS18BP1, C9orf140, WSB1, ZMYM1, TGIF1, ATXN1L, CNTROB, C15orf23, CCDC99, CKS2, IGHA1, HPS4, FOXM1, CDC25B, MKI67, QRICH1, MLLT4, RASGEF1A, LRRC17, HMMR, NEK2, GTSE1, CCDC90B, ERN2, OIT3, RNF126, SS18, NCOA5, NUF2, GAS2L3, SPAG5, C19orf76, BIRC2, GAS6, G2E3, RAD51C, KIF20B, NCAPD2, TLE3, TXNRD1, POC1A, ZFX, TPX2, SGOL2, ORAOV1, HSPA13, CADM1, KIAA0182, TACC3, FRZB, KIF5B, PTP4A1, PRPSAP1, BUB1, DUSP4, CNOT10, DLGAP5, FYN, TXNDC9, C5orf41, THRAP3, USP13, MZT1, SUV420H1, PPP1R10, PBK, DR1, BMP2, CDC20, IDI2, ODF2, ANP32B, CFLAR, CENPA, HSPA8, FAM64A, DNAJB1, INADL, CKAP5, MATN2, LMNA, TNPO1, UBE2D3, ITPR1, ZC3HC1, CCNB2, RANGAP1, E2F5, SRF, TRIP13, TSN, ANP32E, KLHDC9, KIF14, ARL6IP1, MAPK13, NR5A2, PLAG1, MCM4, VCAM1, YWHAH, AHI1, SMARCB1, CDKN2D, OLR1, PLK1, SRSF3, B4GALT1, USP16, GOT1, PAK6, CENPF, DZIP3, AURKA, TFF3, DEPDC1, CEP55, PTGER3, CD97, CTNNA1, SHCBP1, CSGALNACT1, DIAPH3, CCNA2, SFPQ, PHF15, KLF9, INSM1, TMEM138, TTK, HS2ST1, C6, RERE, PIK3CD, SRD5A1, CTNND1, ANLN, ATF7IP, NUSAP1, WIBG, CCDC88A, INPP5K, CENPE, HMG20B, PCF11, ANKRD40, ME3, NDE1, CKAP2, GLI1, ZNF207, ARHGDIB, CDC42EP1, SMARCD1, BIRC5, PTPN9, SLC44A2, SETD8, MDC1, SEPHS1, TNFAIP8L1, KIAA0889, CDCA3, ECT2, PRR5, STAT5B, PSMG3, KCTD2, HP1BP3, AKIRIN2, GRK6, CTCF, DNAJA1, NUP98, SEPN1, KIF2C, SMTN, FGA, PWP1, LBR, RCBTB2, DEPDC1B, ASPHD2, CKS1B, VANGL1, RRP1, TUBB4B, HSPA1L, NUP35, SPTBN1, C1orf96, ASXL1, HN1, TMCO4, ARHGAP19, HERPUD2, TSKU, SLC17A2, POM121, ADH4, LPP, HCFC1, IDO1, RNF141, CNN2, CIT, TSG101, RNPS1, GADD45A

REACTOME_CELL_CYCLE_MITOTIC	Cell cycle	KIF20A, ACTR1A, AKAP9, CDK2, CDK4, CDK6, PSMD14, CDK7, CDKN1A, CDKN1B, STAG1, CDKN2A, CDKN2B, CDKN2C, CDKN2D, TUBB4A, TUBB4B, ANAPC10, NDC80, TUBGCP3, DCTN2, CENPA, CENPC1, CETN2, POLD3, NUDC, PLK4, STAG2, SDCCAG8, TUBGCP2, DBF4, KIF2C, CNTRL, UBE2C, FGFR1OP, ZWINT, CEP250, PMF1, DCTN3, TUBGCP5, CKS1B, NEDD1, LIN54, PSMA8, CSNK1D, CSNK1E, SPC24, SGOL2, SGOL1, DCTN1, DHFR, DNA2, DYNC1H1, DYNC1I2, DYRK1A, E2F1, E2F2, E2F3, E2F4, E2F5, TUBB, SKA1, FEN1, CEP164, MAPRE1, NINL, AZI1, PSME4, CLASP1, ITGB3BP, ORC6, ORC3, POLA2, CDC26, CENPI, AHCTF1, NSL1, FBXO5, TUBG2, PPP2R3B, LIN9, ANAPC2, RPA4, ANAPC4, HDAC1, BIRC5, HSP90AA1, SKA2, NUP43, INCENP, APITD1, KIF2A, LIG1, CENPP, MAD2L1, MAX, MCM2, MCM3, MCM4, MCM5, MCM6, MCM7, MNAT1, LOC440917, LOC441488, MYBL2, MYC, NEK2, NUMA1, ORC1, ORC2, ORC4, ORC5, PAFAH1B1, GMNN, PCM1, PCNA, PCNT, ANAPC5, ANAPC7, ANAPC11, GINS2, PLK1, POLA1, POLD1, POLD2, POLE, POLE2, ERCC6L, CCDC99, PPP1CC, ZWILCH, CEP192, HAUS2, CDCA8, PPP2CA, PPP2CB, CENPQ, PPP2R1A, PPP2R1B, PPP2R2A, PPP2R5A, PPP2R5B, PPP2R5C, PPP2R5D, PPP2R5E, MCM10, PRIM1, PRIM2, PRKACA, CEP72, NUP133, CDK5RAP2, PRKAR2B, CENPJ, CENPN, RCC2, LIN37, PSMA1, PSMA2, PSMA3, PSMA4, PSMA5, PSMA6, PSMA7, PSMB1, PSMB2, PSMB3, PSMB4, PSMB5, PSMB6, PSMB7, PSMB8, PSMB9, PSMB10, PSMC1, PSMC2, PSMC3, PSMC4, PSMC5, PSMC6, PSMD1, PSMD2, CASC5, PSMD3, PSMD4, PSMD5, NUP107, PSMD7, PSMD8, PSMD9, PSMD10, PSMD11, PSMD12, PSMD13, PSME1, PSME2, SPC25, TAOK1, POLD4, RAD21, RANBP2, RANGAP1, RB1, RBBP4, RBL1, RBL2, CCND1, RFC2, RFC3, RFC4, RFC5, RPA1, RPA2, RPA3, RPS27, RPS27A, RRM2, CLIP1, SEC13, CENPK, RBBP4P1, DHFRP1, LOC645084, ANAPC1, GORASP1, TUBBP2, LOC647654, CENPH, LOC649620, SKP1, SKP2, LOC650621, LOC651763, LOC652826, AURKA, BUB1, BUB1B, TFDP1, TK2, TUBA4A, TUBG1, RPS27AP11, SKA2L, TYMS, CDC26P1, LOC729964, LOC730418, LOC730594, UBA52, UBE2D1, UBE2E1, WEE1, XPO1, YWHAE, YWHAG, ALMS1, TUBA1A, MIS12, CENPM, NUP37, CENPO, MLF1IP, NUP85, CEP76, DSN1, CENPT, CEP290, CEP63, CEP70, B9D2, NDEL1, CDT1, SEH1L, KIF18A, SMC1A, CDC7, CDC45, NUF2, MAD1L1, GINS4, MCM8, CUL1, KIF2B, OFD1, TUBGCP6, CDC14A, SSNA1, DYNLL1, CDC23, CDC16, CCNA2, CCNA1, CCNB1, CCND2, BTRC, CCND3, CCNE1, CCNH, PKMYT1, SMC3, CCNB2, CCNE2, CENPL, LIN52, ZW10, BUB3, AURKB, PTTG1, PSMF1, KIF23, CEP41, CEP135, CEP57, KNTC1, CKAP5, CDK1, GINS1, PSMD6, CDC6, CDC20, CDC25A, CDC25B, CDC25C, CDC27
WHITFIELD_CELL_CYCLE_LITERATURE	Cell cycle	CDKN3, RACGAP1, E2F1, PCNA, BIRC5, CCNE2, CDC25C, PSRC1, MCM2, CENPA, MCM6, CDKN1A, CCNB1, RRM2, CCNB2, BRCA2, KIF20A, CDC20, AURKA, CDC45, NASP, CCNG2, CDK1, TYMS, CDKN2D, BRCA1, TOP2A, NPAT, E2F5, CKS2, BUB1, CCNE1, BUB1B, CDC6, CCNF, SLBP, MSH2, CDKN2C, CCNA2, RRM1, CDC25A, PLK1, DHFR, CENPF
PID_AURORA_A_PATHWAY	Cell cycle	GSK3B, GIT1, ARHGEF7, CKAP5, TDRD7, BRCA1, BIRC5, RAN, AURKB, NDEL1, PAK1, AKT1, TPX2, DLGAP5, TACC1, PPP2R5D, OAZ1, TACC3, PRKACA, NFKBIA, GADD45A, CDC25B, FZR1, AURKA, CENPA, RASA1, MDM2, CPEB1, JUB, TP53, AURKAIP1
BROCKE_APOPTOSIS_REVERSED_BY_IL6	Apoptosis	SOX2, STAT3, SH2D2A, HNRNPU, STAT1, SBNO2, ANAPC10, WIPI1, DUSP3, HBEGF, RCN2, MCL1, RB1, LITAF, CADPS, PTP4A3, RALA, JUNB, DPM1, MT1X, RNF13, GADD45A, FLOT1, ZFP36, IRF4, IL6ST, SLC2A3, ID3, ST3GAL6, IER2, GATM, DUSP5, SGK1, MUC1, HSPA13, PNO1, EPB41L2, MAP3K8, CD8B, SLA, DAPK1, CD44, MAP1B, ACPP, PRDM1, MYLK, LRMP, TXNDC9, MX1, FOXO3, LTBP1, PIM1, TNFSF10, AMPD1, PHTF2, HSPA1A, SREK1IP1, MAPKAPK2, PIM2, ICAM1, SPP1, ID1, EEF1E1, NAMPT, POLR3F, POU2AF1, CEBPB, DYRK3, CHST15, DNAJA2, ARFGAP3, BCL6, PTPRG, GRB2, MSMO1, IRF9, ZBTB11, ELL2, SRPK2, TGIF1, RGS16, PDGFRA, LDLR, GADD45B, PLSCR1, OAS1, C10orf10, BHLHE40, IFNGR1, AP1S2, POU2F2, TWF1, NMI, CCNC, NCBP2, SOCS1, ZNF101, HHLA1, SOCS3, DNAJB9, HCK, CCL2, PLEKHB2, PTP4A1, PTPN11, GNA13, MAZ, SLC16A6, HEG1, KIAA0125, MARCKS, MT1H, ATP2B4, TMF1, EVI2B, TMED7, FAM3C, VWA5A, EVI2A, CDK17, GAS6, DOHH, TPM4, PAN2, IRF1, RAD1, CXCR4, CAV2, SF1, SIK1, ZFP36L2, RHOBTB3, LPCAT4, EIF4E, CD180, MAFF, BCL2L11, ADIPOR2, JUN, STK17B, RAPGEF4, BCL3, EIF5, TMEM184B
CONCANNON_APOPTOSIS_BY_EPOXOMICIN_UP	Apoptosis	EIF5, GDF15, RHBDD3, LRIG1, DNAJB6, ALDH1A2, PLK2, PLAT, SLC7A5, IL15, C18orf25, DUSP1, ZFP36, ORC3, IL6R, ABHD3, THBS4, TGFBR2, GTF2A1, CAP2, MICB, FOS, DUSP3, COL16A1, CLTB, PCSK1, GLS, KLHL21, ZFR, SLC7A1, SMG1, ATF3, TTC39A, ARHGEF2, LPXN, CASP7, PHLDA2, SSX1, SPP1, KIAA0284, HEG1, SLC16A3, CREM, DHRS3, POLR3C, SLC38A6, PRSS12, SYT2, YKT6, ACHE, PLD3, SYNJ2, NEAT1, SYCP2, GOSR2, LMNA, TMEM159, LMO2, HMOX1, CAMK2G, LAMP3, SERPINH1, PSMA5, C6orf145, STK17A, GEM, SPATA2, HSPA1B, DGKE, DNAJC2, TNPO2, MXD1, SLC3A2, PSMD12, NACC2, SSX3, MAGEB1, ZYX, SAT1, CTH, MAP2, ANXA2, CAPN7, GLRX3, PPP1R15A, SEL1L3, NQO1, DUSP4, TUBA4A, CYP4F2, STX6, C1S, DAP3, PSMD1, RRAD, MIR22HG, ELL2, MDM2, PCYT1A, TOP3B, SLCO3A1, UBFD1, AGTPBP1, SMOX, SERPINE1, PSMD11, F2RL1, TMF1, JUN, KCTD20, LIN37, ABCG1, STX16, CREB5, ATP2B4, TSPAN9, MAFF, P4HA2, EPCAM, NQO2, TAF13, AKAP6, NSFL1C, DNAJB1, SNRK, CYB5R1, SERPINB8, AQP3, GCLM, NTS, CDKN1A, ABLIM3, DTNA, SKIL, RAB36, RIT1, ME1, TAC1, BLVRB, CHM, DOK1, KIAA0319, WWTR1, IFRD1, WARS, MAP1A, FAS, GSR, MSX2, HSPA4, HSPA6, HSPB1, CDK11A, DYNC1I1, BSCL2, ACADVL, CDR1, EPB41, DDIT3, CD44, CALCA, RAB21, MEST, BCL2L11, CEBPB, ITGA7, ADRM1, KLF6, FLNC, HSPA9, PALLD, HSPA4L, DNAJB4, PRKCZ, N4BP2L2, SLC12A4, GPX3, TNFSF9, DNAJB2, SLC7A11, KIAA0930, PRDM2, TNKS, MAPK10, BBC3, PHTF1, ZNF10, TOM1, NUCB1, SQSTM1, HSPH1, BAK1, PDLIM3, LGALS8, HSPA1A, TSPYL2, GADD45G, AKR1C3, CEBPG, FAT1, IDE, CLU, PTPRN, UBE2H, ZNF185, PSMD13, MAP1B, JMJD6, MAP1LC3B, TTC1, PHKG2, GADD45A, PLA2G16, TNFRSF10B, STIP1, ZRSR2, IL11, ACTA2, ICAM2, ZRSR1, CCPG1, FILIP1L, GLA, UPP1, RRN3, INPP5D, UBR4, C11orf80, KITLG, MBOAT7, UFD1L, PCYT1B, KIF1B, DYNC1H1, PGF, SUSD5, MAP3K14, CCNT2, GABARAPL1
GARGALOVIC_RESPONSE_TO_OXIDIZED_PHOSPHOLIPIDS_GREY_UP	Cellular stress	KIAA1522, EMP1, HERPUD2, RORA, SOS1, NAV3, DUSP5, ADAMTS1, LBH, ARRDC4, ZNF323, TFEC, CCDC68, FNIP1, IRAK2, ADAMTS9, TRIM16, C5orf41
GARGALOVIC_RESPONSE_TO_OXIDIZED_PHOSPHOLIPIDS_BLACK_UP	Cellular stress	PLAGL1, AKIRIN2, CLK1, TFEC, ZNF44, CD55, SQLE, TRIB3, SPRY4, CCDC93, GCH1, EIF1, SNX16, OSGIN2, MOB2, ATP11B, ERRFI1, F2RL2, RGMB, PRNP, SRC, LATS2, ZNF295, CDKN1A, DUSP14, ZNF654, CCDC82, NFE2L2, UBB, ETV5, LOC284023, KLF2, ABHD4, CDC42EP2, RHPN2
GARGALOVIC_RESPONSE_TO_OXIDIZED_PHOSPHOLIPIDS_LIGHTYELLOW_UP	Cellular stress	CSGALNACT2, GNA13, ZDHHC18, PNPLA8, RBBP6, UGDH, ABHD3, IER5, USP12, PIM1, SIRT1
GARGALOVIC_RESPONSE_TO_OXIDIZED_PHOSPHOLIPIDS_BLUE_UP	Cellular stress	INHBA, FRS2, N4BP2L2, SOS1, TRIB1, DEDD2, WDR20, TNFSF9, IL8, ZC3H12C, ZNF264, CPEB4, KITLG, CXCL3, ZNRF3, PCYT1A, KLHL28, RHEBL1, KIF1B, PPP3CC, SPIRE1, STIP1, MIR22HG, HSPH1, LOC100133660, DNAJB1, SAV1, SESN2, SAMSN1, RELB, PHF1, SERTAD1, AKIRIN2, CWC25, HSPB8, LOC92659, ATF3, NFATC2, YRDC, SMCR8, C16orf72, RBBP6, CASP3, KLF5, VCPIP1, RNF24, BRF2, HERPUD1, JUND, C20orf111, RYBP, DNAJB9, PMAIP1, MXD1, MAFG, IFRD1, CBLL1, ZNF222, CREM, MTHFD2L, DDIT3, FAM100B, TMEM57, ZNF468, AGPAT9, EIF1, AHSA1, ANKRD57, C10orf88, SLC3A2, HBEGF, MAFK, C16orf52, KBTBD8, JMJD6, ZNF473, RGMB, E2F6, RCAN1, CNST, BANP, LOC100302650, C9orf150, DNAJA4, RORA, RRP7A, JHDM1D, EID3, RELN, RTCD1, NFIL3, KCTD5, FGFR1OP2, RBM33, SRXN1, SLC19A2, DNAJA1, EIF5, C11orf84, TMEM217, HSPA1A, AIFM2, KIF21A, ZNF394, PLAUR, PNPLA8, CHORDC1, SMURF1, ESM1, VPS37A, ETV5, F3, ZNF295, DNAJB6, C6orf145, STK17A, ZBTB43, CCNG2, HSPA4L, FOSL1, GNA13, PCF11, C2orf67, TSC22D3, HSPA6, KLHL21, BACH1, LOC100505728, AMMECR1L, RSL1D1, GLA, CREBBP, EIF2C2, ZFAND2A, PNP, BAG3
KAN_RESPONSE_TO_ARSENIC_TRIOXIDE	Cellular stress	MT1X, EIF4G1, VCAN, DKK1, IGF2BP3, SOX2, AKR1C2, ARHGAP6, HSPA6, DPYSL3, ID2B, MGP, IL11, IL7R, FAM64A, ENO2, SCD, MT1E, SPC25, DDIT3, PTN, FABP7, KIF20A, JAG1, RGS4, PSAT1, MAP1LC3B, SCG2, TOP2A, IL8, IGFBP5, GABARAPL1, GSTM3, LMNB1, NDRG1, CLEC2B, NUPR1, SERPINE1, SLC7A11, MYBL1, PTPRO, ARL4C, GRN, BOP1, TRIB3, MT1H, STC2, PTHLH, AK4, FAM162A, MT1F, ADM, MT2A, GDF15, AKR1C1, HEY1, TRIM16, BNIP3L, CBS, DLGAP5, ATF3, SLC30A1, JUN, PIR, DIRAS3, MCM7, C6orf15, TSC22D3, VAT1, ATP6V0E1, AKR1C3, THBS1, GPM6B, PHLDA1, MYCNOS, SEL1L3, CRYAB, AKR1B10, CAV2, CCPG1, DCBLD2, GFPT2, DDIT4, PDGFRA, GLRX, FBXO5, CTH, EFNB2, WIPI1, ID2, SLC12A1, ASNS, ANKRD1, SACS, PHGDH, LDLR, TMEM158, CXCL14, STC1, LGALS8, FABP4, PGK1, PODXL, RIT1, MT1P2, INSIG1, PLK2, KLF12, ARHGAP29, PPP1R15A, GPR65, ZNF512B, RRM2, PTPRZ1, FEZF2, SOCS2, HMOX1, KCTD12, NES, NOX1, NDP, GPNMB, MT1G
WEIGEL_OXIDATIVE_STRESS_BY_TBH_AND_H2O2	Cellular stress	RPA1, ITGB5, CAPN2, CDK10, ACTB, ITGB4, SLC7A5, TCEA1, ZYX, DYNLL1, PPP1R8, TP53, SF1, TPP1, CTNNA1, SPINT2, VCAM1, HDAC1, FMO1, CDKN2B, RPL13A, RB1, ITGAL, TUBA1B, SRF, BAK1, HLA-C, CDH11, ERVW-1, CDC25C, QSOX1, BMP4, MMP11, RNH1, HGFAC, HSPA13
GARGALOVIC_RESPONSE_TO_OXIDIZED_PHOSPHOLIPIDS_TURQUOISE_UP	Cellular stress	ARHGAP5, CCNG2, FAM126B, JUN, DUSP1, LOC344887, MGC11082, CCDC117, GABARAPL1, TNFSF18, KLF6, PRNP, NCOA3, NAB2, RIT1, SLC4A7, CCDC82, SLC7A11, FOSL2, AKAP12, ANKRD12, RRAGC, AHR, PTP4A1, ARRDC3, ZNF627, PTGR1, CDC42EP2, C15orf39, RICTOR, EGR1, ZBTB34, MYC, CLIC2, SLC25A25, RORA, GCLM, SQSTM1, EIF2C2, DIDO1, ACVR1, SAMD8, TNPO1, ZNF264, MERTK, TIPARP, SMG1, NRG1, CNKSR3, C5orf41, EMP1, GPCPD1, PIM3, DUSP3, KLF10, MAP1B, AFF4, CLDND1, PANK3, HIPK3, MYCT1, SPIRE1, CLDN15, SPOPL, CFLAR, ZNF697, PGF, HS6ST1, PVRL3, SPRY2, FGFR1OP2, CLIP2, GDF15, SGK1, LINC00304, ZCCHC2, IPMK, ZFP36, NIPA1
GARGALOVIC_RESPONSE_TO_OXIDIZED_PHOSPHOLIPIDS_YELLOW_UP	Cellular stress	C5orf30, C16orf52, KLF4, LCOR, NUP153, PPP3R1, C16orf72, OSGIN1, HMOX1, GK, TNPO1, KITLG, UNKL, JHDM1D, LDLR, PHLPP2, TULP3, ENTPD7, NPC1, GCLC, ATXN7, C10orf88, CHIC2, CNST, NAV3, C3orf58, RAB8B, JOSD1, GCLM, SGMS2, XPO1, SLC7A1

Supplementary Table 8. Statistical analysis for LDA shown on Supplementary Fig. S14B (up) and 5E (bottom) using ELDA software.

Group 1	Group 2	Chi-sq	DF	Pr (> Chi-sq)
IgG	Anti-MDK	3.19	1	0.0742
IgG	AZD7762	4.2	1	0.0403
IgG	Anti-MDK/ AZD7762	27.6	1	0.000000149
Anti-MDK	AZD7762	0.0499	1	0.823
Anti-MDK	Anti-MDK/ AZD7762	12.1	1	0.000492
AZD7762	Anti-MDK/ AZD7762	11.2	1	0.0008

Group 1	Group 2	Chi-sq	DF	Pr (> Chi-sq)
IgG	Anti-MDK	3.19	1	0.0742
IgG	CHIR-124	24.3	1	8.41E-07
IgG	Anti-MDK/ CHIR-124	72.4	1	1.79E-17
Anti-MDK	CHIR-124	9.86	1	0.00169
Anti-MDK	Anti-MDK/ CHIR-124	47.5	1	5.50E-12
CHIR-124	Anti-MDK/ CHIR-124	16.9	1	3.88E-05
